# Imine-Based Transient Supramolecular Polymers

**DOI:** 10.1021/jacs.5c00274

**Published:** 2025-03-19

**Authors:** Gabriele Melchiorre, Lucia Visieri, Matteo Valentini, Roberta Cacciapaglia, Alessandro Casnati, Laura Baldini, José Augusto Berrocal, Stefano Di Stefano

**Affiliations:** † Dipartimento di Chimica and Istituto CNR per i Sistemi Biologici (ISB-CNR), Sede Secondaria di Roma—Meccanismi di Reazione, c/o Dipartimento di Chimica, Università di Roma “La Sapienza”, P.le A. Moro, 5, Rome I-00185, Italy; ‡ Dipartimento di Scienze Chimiche, della Vita e della Sostenibilità Ambientale, Università degli Studi di Parma, Parco Area delle Scienze 17/A, Parma 43124, Italy; § Barcelona Institute of Science and Technology (BIST), Institute of Chemical Research of Catalonia (ICIQ), Avda. Països Catalans, 16, Tarragona E-43007, Spain

## Abstract

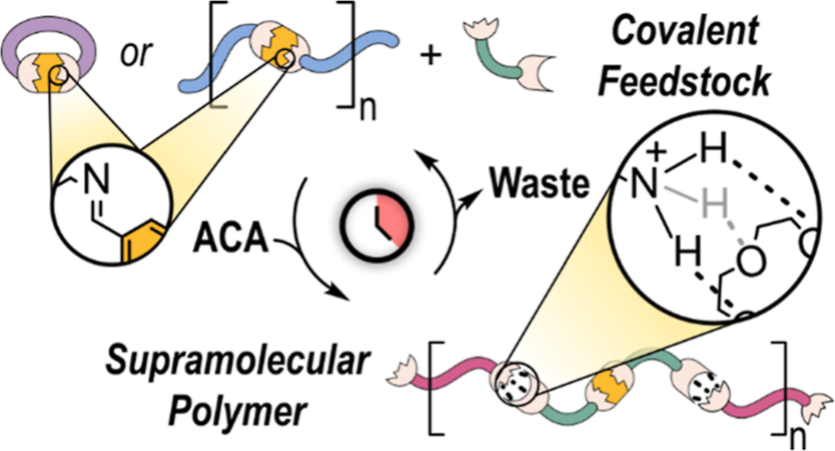

Systems that change properties upon exposure to chemical stimuli offer the interesting prospect of (partially) mimicking the functions of living systems. Over the past decade, numerous supramolecular systems whose chemical composition and properties are regulated by the dissipation of chemical fuels have been reported. These systems are typically based on the transient transformation of a “dormant” species into an active, self-assembling supramolecular monomer. The process is powered by fuel consumption and terminates upon fuel depletion, restoring the initial dormant state. Previously reported out-of-equilibrium supramolecular polymerizations relied on the activation of the dormant species by adding or removing small structural units to enable supramolecular polymerization. Here, we present an approach that combines the reversibility of dynamic covalent chemistry and supramolecular chemistry to trigger transient supramolecular polymerizations by “recycling” the components of a dynamic combinatorial library (DCL). Treatment of an equilibrated DCL of aliphatic imines and aromatic amines with an activated carboxylic acid (ACA) generates a dissipative dynamic combinatorial library of aromatic imines and protonated aliphatic amines. The transient acidic conditions enable the creation of a supramolecular polymer held together by interactions between the protonated aliphatic amines and the crown ether moieties embedded in the scaffold of the aromatic imines. Thus, fuel dissipation reshuffles the chemical connectivity and enables the temporary transformation of a purely covalent (polymeric) system into a supramolecular polymer. We demonstrate the strategy using two different covalent dormant feedstocks consisting of a diimine macrocycle involving a calix[4]arene scaffold and a distribution of imine (cyclo)oligomers derived from an isophthalaldehyde skeleton.

## Introduction

Supramolecular polymers and materials represent an increasingly attractive research topic, mainly due to their reversible nature. In fact, being based on reversible interactions such as hydrogen bonding, π–π stacking, metal–ligand coordination, hydrophobic/hydrophilic interaction, and the like,^1^ they are particularly prone to rapidly respond to external stimuli, leading to, for example, (polymer) materials with adaptive and self-healing behavior.^2^

A great interest has been devoted to dissipative systems in the past decade^3^ due to the aspiration of researchers to artificially reproduce, at least in part, some of the characteristic features of living systems. The physicochemical properties of a dissipative system can be transitorily varied by the addition of a stimulus, which can be radiative or chemical in nature. When a chemical species is used as the stimulus (sometimes termed fuel), it is consumed (or dissipated) during a cycle in which the system acts as a catalyst for such a dissipation. Such a process transitorily changes the chemical nature of the system, consequently altering its physicochemical properties as well. When the stimulus is exhausted, the system and related properties go back to the initial state, closing the cycle. Many dissipative systems reported in the literature are based on supramolecular polymers or supramolecular assemblies.^4^ Such studies are clearly inspired by processes occurring in cells, as, for example, the reversible formation of actin filaments^5^ or microtubules.^6^ In these cases, the chemical stimulus is used to chemically activate a precursor into an activated monomer^7^ that is able to transitorily self-assemble into a complex supramolecular structure (the polymer). This is followed by an autonomous deactivating reaction that restores the precursor, with the consequent disruption of the polymer.^8^ This general idea has been leveraged to trigger time-controlled self-assembly resorting to methylation,^9^ acylation,^10^ imination^11^ reagents, disulfide exchange,^12^ olefin metathesis,^13^ and acid–base^14^ or redox reagents.^15^

Here, we describe a novel strategy for the activation of precursors for dissipative supramolecular polymers that builds on the reversibility of dynamic covalent bonds and supramolecular interactions. One molecular unit, initially present in the covalent skeleton of a precursor, is transiently activated by a chemical stimulus that gives rise to a transient supramolecular polymer that will persist as long as the stimulus is present. When the stimulus is exhausted, the covalent precursor can be reversibly restored.

**Figure 1:**
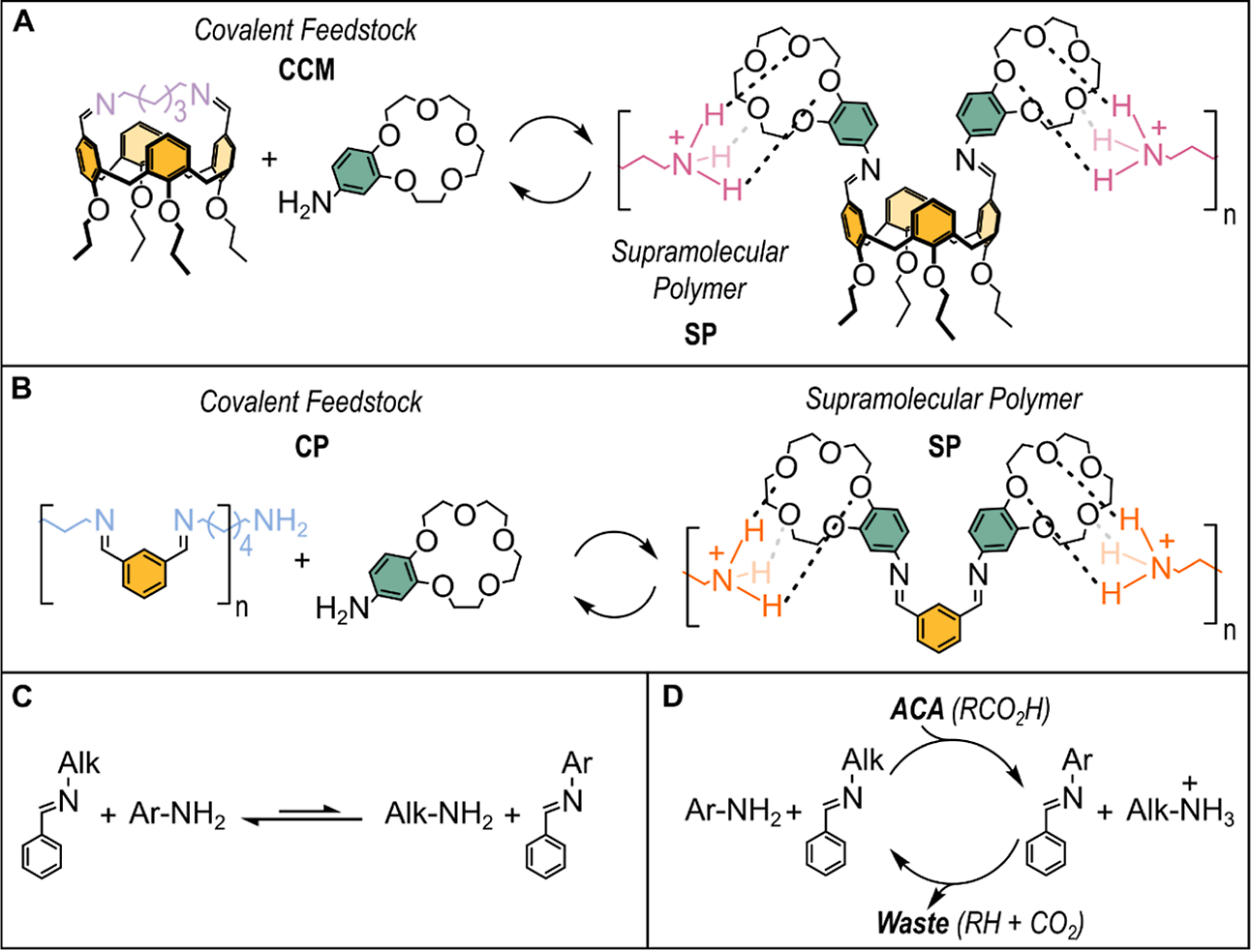
(A) A transient supramolecular polymer obtained from a covalent cyclic monomer (CCM) by activation of one of the structural units present in the monomer. (B) A transient supramolecular polymer obtained from a covalent polymer (CP) by activation of one of the structural units present in the covalent feedstocks. (C) The equilibrium among *N*-aromatic (Ar-) and *N*-aliphatic (Alk-) imines and related amines is naturally shifted to the *N*-aliphatic imine side. (D) Transient protonation of the aliphatic amine by the ACA temporarily shifts the equilibrium in panel C toward the *N*-aromatic imine side. Such shift persists as long as the ACA is present.

Due to its reversibility and capability to respond to external stimuli such as the addition of acids and variations of temperature, imine chemistry seemed to be a good candidate to design a transient supramolecular polymer. In particular, we planned to employ imines in the form of a covalent cyclic monomer (CCM) or a covalent polymer (CP) as the main material reservoirs (Figure 1A,B, respectively). The envisioned CCM and CP imine systems feature a calix[4]arene dialdehyde and isophthalaldehyde skeleton, respectively. We anticipate that these two scaffolds yield imines with different compositions when reacted with small, aliphatic diamines. The covalent feedstocks are completed by the aromatic amine that accompany the CCM and CP (Figure 1A,B). The simultaneous presence of aliphatic and aromatic amines in the material reservoirs should not yield complex mixtures of *N*-aliphatic or *N*-aromatic imines as the equilibrium involving *N*-aromatic and *N*-aliphatic imines and related amines is shifted toward the *N*-aliphatic imines (Figure 1C).^16^ However, such an equilibrium can be overturned by the addition of a stoichiometric amount of an acid that quantitatively protonates the strongest base, i.e., the aliphatic amine. The latter is subtracted and transformed into the corresponding ammonium ion.^17^ It should be noted that besides dramatically changing the equilibrium between the two imines, the acid also catalyzes the transimination reaction. Similarly to “conventional” acids, activated carboxylic acids (ACAs, RCO_2_H)^18,19^ can also be used to drive the variation of the composition of a dynamic library of imines. The main difference is that the use of ACAs leads to a dissipative cycle in which the perturbation of the composition of the library persists as long as the ACA is present (Figure 1D). The decarboxylation of ACA is, in fact, promoted by the bases present in solution and occurs smoothly, i.e., with no side products apart from the decarboxylated one (RH, the waste product).

Combining all of these elements, here we show that an ACA could be employed to transiently transform the feedstocks of Figure 1A,B into supramolecular polymers (SPs). The transient protonation of the strongest base in solution (the alkyldiamine) leads to a supramolecular polymer in which one of the two building blocks (the alkyldiammonium) is the protonated (activated) form of one of the structural units initially present in the covalent backbones of CCM or CP. At the end of ACA decarboxylation, the system returns to the initial state, closing a dissipative cycle of the kind CCM → SP → CCM or CP → SP → CP.

## Results and Discussion

### Characterization of the Covalent Feedstocks

**Figure 2:**
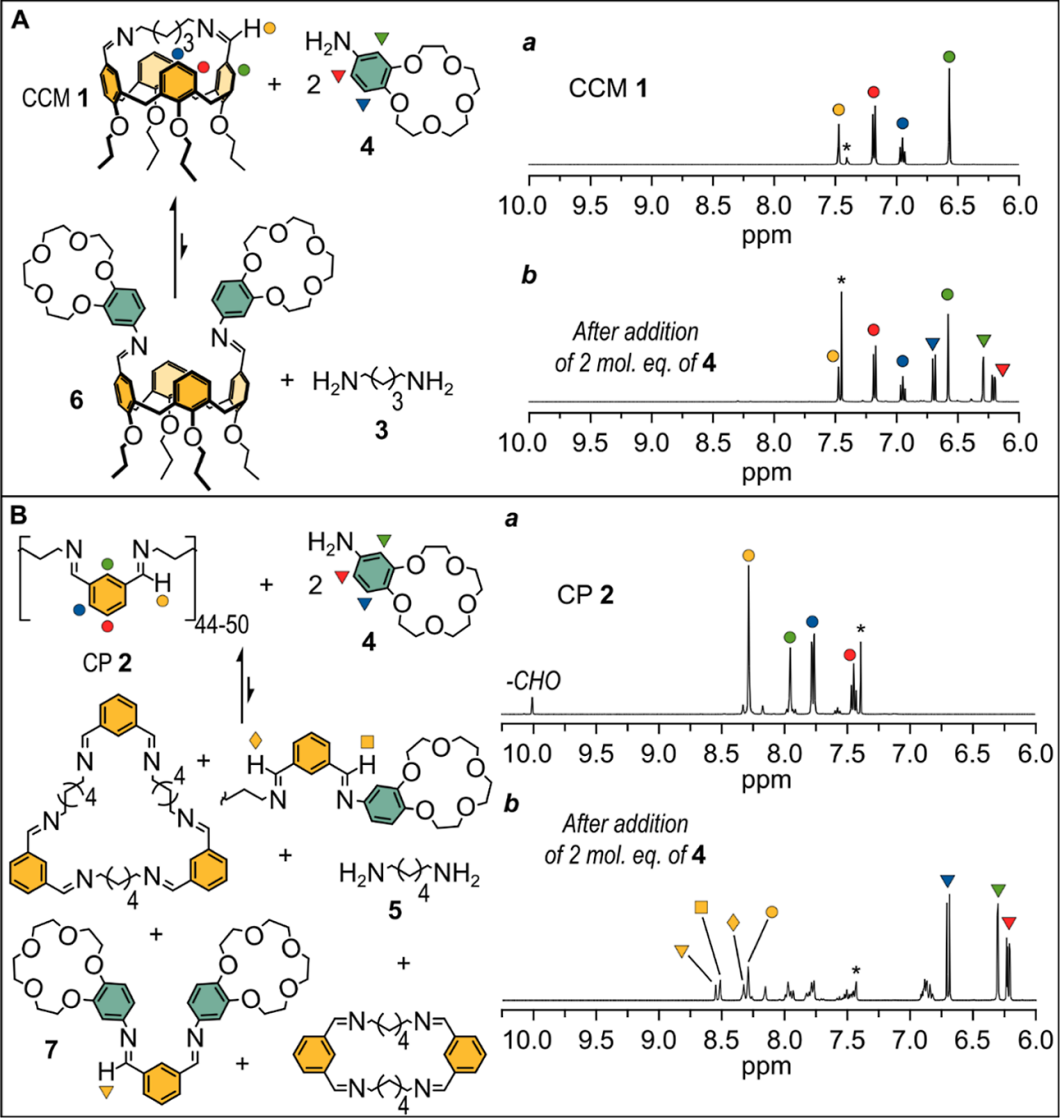
(A) Partial ^1^H NMR spectra of CCM **1** before (trace *a*) and after (trace *b*) the addition of **4** (CDCl_3_/CD_3_OD 3:1). Trace *b* is related to a 200 mM **1** + 400 mM **4** solution warmed at 50 °C for 5 days. Prolonged warming and consequent change of medium composition are responsible for the downfield shift of the chloroform signal, which is marked with an asterisk. (B) Partial ^1^H NMR spectra of CP **2** before (trace *a*) and after (trace *b*) the addition of **4** (CDCl_3_/CD_3_OD 3:1). Trace *b* is related to a 100 mM CP **2** + 200 mM **4** solution warmed at 50 °C for 1 day. Species present in the mixture of trace *b* were identified by a combination of ^1^H NMR, ESI–MS, and APCI–MS spectra, see Supporting Information pages S18–S20 for details (complete NMR spectra are given in Figures S2, S8, S17, and S18 of the Supporting Information).

CCM **1** (Figure 2A) was prepared by reacting equimolar amounts of the parent *cone*-calix[4]arene 1,3-diformyl derivative and 1,5-pentamethylendiamine **3** (cadaverine) in toluene (see Supporting Information, page S6). Proton and carbon nuclear magnetic resonance (^1^H NMR and ^13^C NMR) spectroscopy, electrospray ionization–mass spectrometry (ESI–MS) and atmospheric pressure chemical ionization (APCI)–MS confirmed the nature of **1**. This chemical design featuring a calix[4]arene in which the distal positions at the upper rim are spanned by a short aliphatic spacer was chosen as a source of material due to its high stability. Indeed, previous reports estimated an effective molarity (EM) as high as 1.5 M^20^ for a very similar structure with four ethoxyethoxy groups (−OCH_2_CH_2_OCH_2_CH_3_) at the lower rim instead of the four propoxy groups of **1**. The high stability of CCM **1** was confirmed by ^1^H NMR experiments in 3:1 v/v CDCl_3_/CD_3_OD, in which 400 mM 4′-aminobenzo-15-crown-5 **4**(21,22) was added to a 200 mM solution of **1**.^23^ CCM **1** remained substantially untouched even after prolonged warming at 50 °C (5 days), with negligible traces of transimination or hydrolysis products (compare trace *a* to trace *b* in Figure 2A).

CP **2** was obtained by reacting equimolar amounts of isophthalaldehyde and 1,6-hexamethylendiamine **5** in toluene at reflux at three different concentrations (100 mM, 500 mM, and 1.00 M) (see Supporting Information, pages S9–S16). The size exclusion chromatography (SEC) traces of the 100 and 500 mM reaction mixtures suggested that these polymers had a comparable polymerization degree (DP) of about 44–50 (see Supporting Information Figures S9 and S13), while the DP of the polymer prepared at 1.00 M was about 110 (Figure S16). We note that these DP values are only approximate as they derive from a calibration curve built on polystyrene standards, whose chemical structure differs significantly from the polyimines investigated here. While SEC suggested successful polymerizations, the ^1^H NMR spectra (3:1 v/v CDCl_3_/CD_3_OD) of the materials prepared at 100 and 500 mM showed that partial hydrolysis had taken place, with the appearance of a non-negligible aldehyde signal, corresponding to ca. 10% of the whole feedstock (see trace *a* in Figure 2B). Furthermore, when such polymers were dissolved in the same solvent mixture (3:1 v/v CDCl_3_/CD_3_OD) at an equivalent monomer concentration of 100 mM together with 200 mM **4**, we observed partial transimination upon warming at 50 °C for 1 day (see trace *b* in Figure 2B). This led to the equilibrated^24^ library of cyclic and linear oligomers depicted in Figure 2B, which were detected by means of ^1^H NMR, ESI–MS, and APCI–MS. Thus, it has to be stressed that when CP **2** is mixed with **4**, a mixture of covalent oligomers and cyclo-oligomers is formed rather than a covalent polymer.

Overall, CCM **1** and CP **2** displayed starkly contrasting behavior upon the addition of 2 mol equiv of **4**. While CCM **1** remained almost untouched, CP **2** was transformed into a mixture of linear and cyclic oligomers via transimination. The behavior of these two imine systems can be rationalized considering the EMs of cyclic species comprising *para*- and *meta*-xylylene spacers (the last one is reminiscent of isophthalaldehyde) vis-à-vis other cyclic species featuring the calixarene scaffold. Cyclic oligomers derived from *para*- and *meta*-xylylene spacers^25^ displayed much lower EMs, and hence much lower stability, whereas cyclic monomers bearing the *cone*-calix[4]arene spacer possess high EM values and stability.^20^

### Characterization of the Supramolecular Polymers

**Figure 3:**
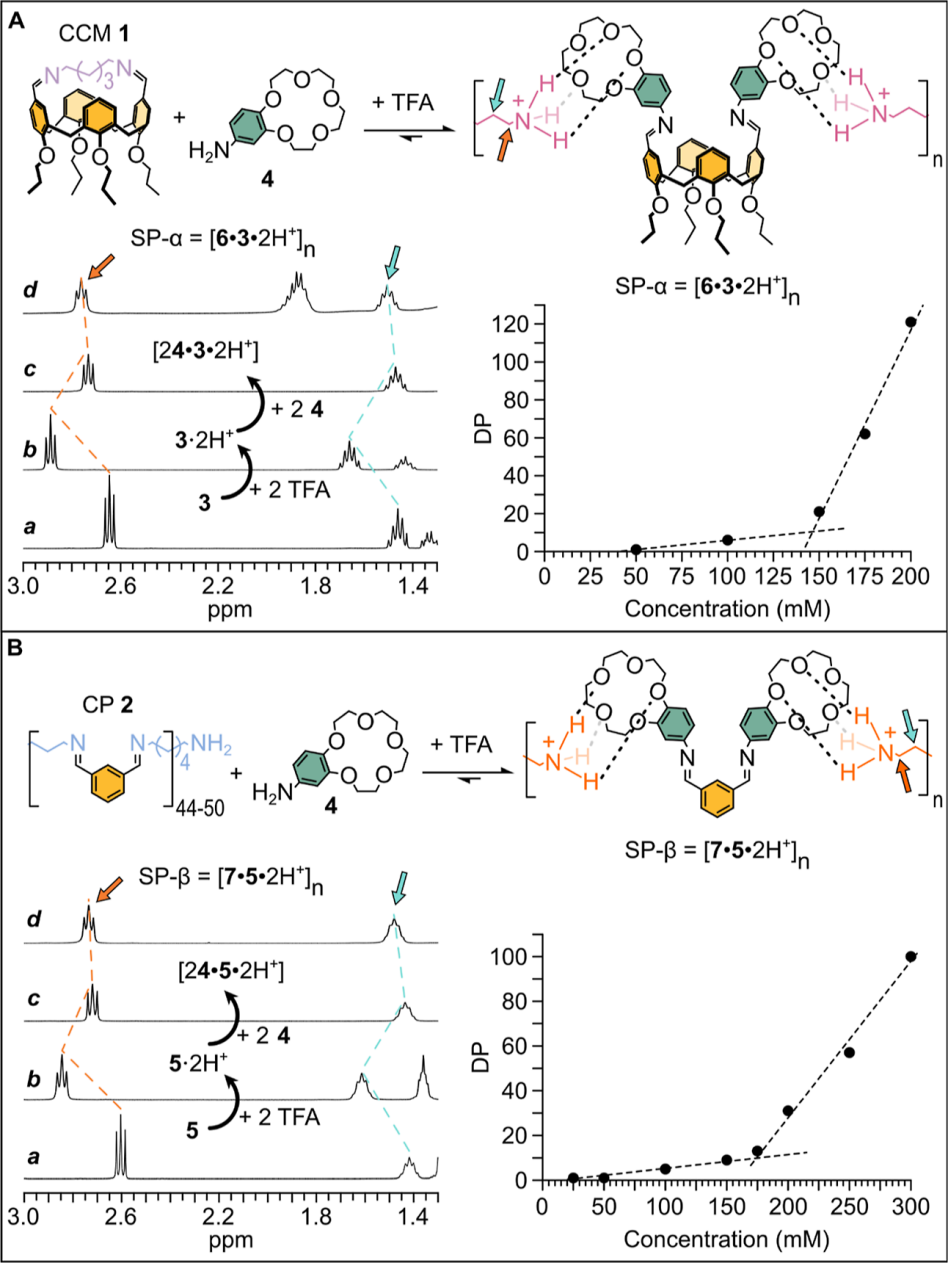
(A) Partial ^1^H NMR spectra (CDCl_3_/CD_3_OD 3:1) of **3** (trace *a*), **3**·2H^+^ (trace *b*), 1:2 **3**·2H^+^ + **4** (trace *c*), and 1:2:2 CCM **1** + H^+^ + **4** (trace *d*) (see Figure S41 for complete spectra). The plot shows the dependence of DP in SP-α as a function of concentration, calculated from DOSY experiments (see Supporting Information, pages S33 and S34). (B) Partial ^1^H NMR spectra (CDCl_3_/CD_3_OD 3:1) of **5** (trace *a*), **5**·2H^+^ (trace *b*), 1:2 **5**·2H^+^ + **4** (trace *c*), and 1:2:2 CP **2** + H^+^ + **4** (trace *d*) (see Figure S70 for complete spectra). The plot shows the dependence of DP in SP-β as a function of concentration calculated from DOSY experiments (see Supporting Information, pages S34 and S53–S54).

We added trifluoroacetic acid (TFA) to mixtures of CCM **1** and **4** of increasing concentration of **1** (from 50 to 200 mM, in CDCl_3_/CD_3_OD 3:1 v/v). CCM **1** and **4** were mixed in a 1:2 ratio, and TFA was added in equimolar amounts with respect to **4**, which corresponds to equimolar amounts of the imine functions (Figure 3A). Protonation (to a large extent) of the strongest base **3** to **3**·2H^+^ caused the overturning of the equilibrium, which was shifted from the *N*-aliphatic imines present in CCM **1** to the *N*-aromatic imines in the SP. The SP built in this specific case is hereafter referred to as SP-α = [**6**·**3**·2H^+^]_*n*_ as the transiently formed dication **3**·2H^+^ acts as a bridge between the crown-endowed difunctional calixarene **6** (Figure 3A). The rearrangement of the components of the system was visible by ^1^H NMR spectroscopy (Figure 3A). The ^1^H NMR trace *d* in Figure 3A shows that, after the addition of 400 mM TFA to 200 mM **1** and 400 mM **4**, CCM **1** is likely transformed into SP-α, thanks to the extensive coordination of the crown-ether moieties by the ammonium heads of **3**·2H^+^.

**Figure 4:**
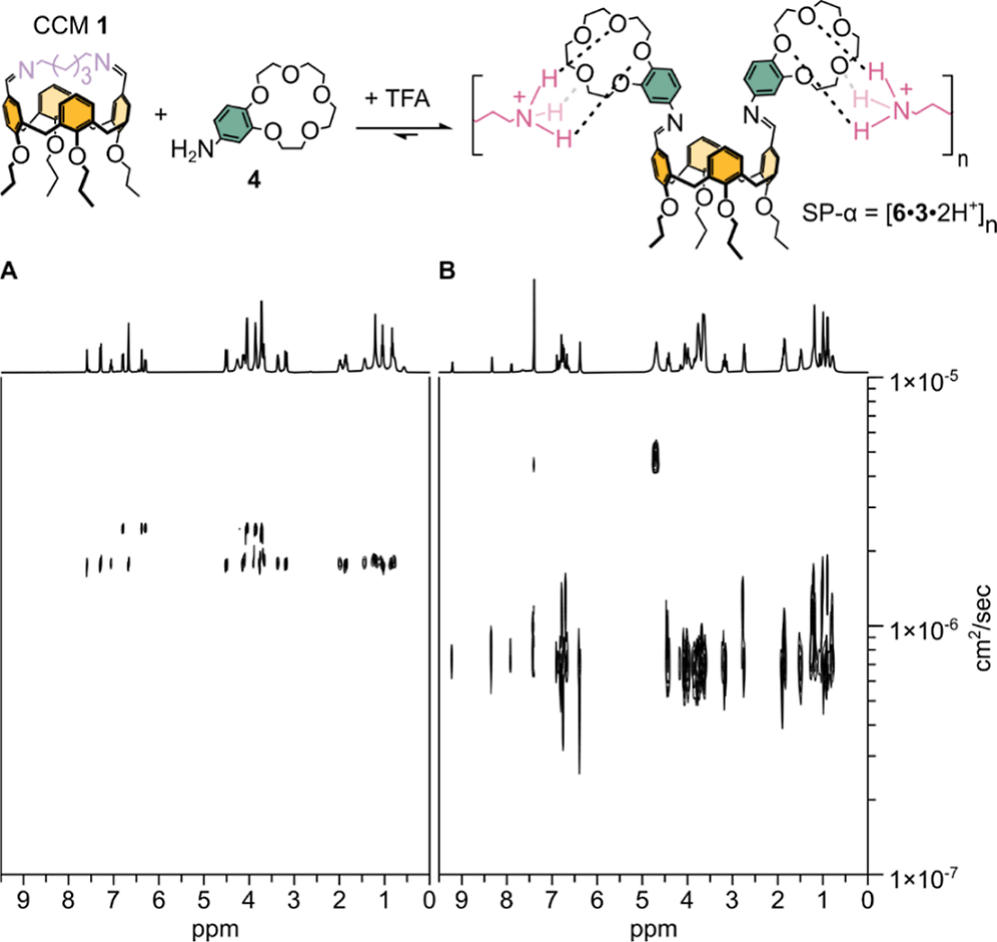
2D DOSY spectra (400 MHz, CDCl_3_/CD_3_OD 3:1) of CCM **1** and **4** mixed in a 1:2 molar ratio (200 mM **1** and 400 mM **4**) before (A) and after (B) the addition of TFA (400 mM). The addition of TFA results in the formation of SP-α.

A corroborative and more convincing evidence for the formation of SP-α comes from a series of two-dimensional diffusion-ordered spectroscopy (2D-DOSY) NMR experiments carried out on solutions in which **1**, **4**, and TFA were mixed in a 1:2:2 ratio and that were prepared at different concentrations of **1** (Figure 4 shows the case of 200 mM **1**). The 2D-DOSY experiments allowed us to measure the diffusion coefficients (*D*) of all of the species present in solution, including the *N*-aromatic imines. Prior to the addition of TFA, we detected two, noninteracting species, i.e., **1** and **4**, with *D* equal to 1.8 × 10^–6^ (**1**) and 2.5 × 10^–6^ cm^2^/s (**4**) (Figure 4A). The addition of TFA to the 1:2 mixture of **1** and **4** resulted in a dramatic change, affording a more slowly diffusing species with a monomodal distribution centered at 7 × 10^–7^ cm^2^/s (Figure 4B). Importantly, the D related to all of the signals of **3·**2H^+^ exactly coincides with that of the above *N*-aromatic imine functions (related to **6**), strongly suggesting the formation of SP-α (see Figure S24). The species with *D* 4.2 × 10^–6^ and 4.9 × 10^–6^ cm^2^/s in Figure 4B are assigned, respectively, to CDCl_3_ and CD_3_OD, instead.

The *D* determined by 2D-DOSY enabled the calculation of the DP of the supramolecular polymers using eq 1 derived from the Stokes–Einstein equation
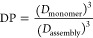
1

We point out that the DP determined with this approach is limited by the assumption of the Stokes–Einstein equation of dealing with spherical particles, a model that is likely not accurate for **1**, **4**, and SP-α in solution. Nevertheless, eq 1 is useful to compare DPs determined across a concentration series, as investigated here (50–200 mM **1**, Figure 3A and Table S4). The plot of Figure 3A clearly shows that DP increases with an increase in the concentration of **1** (see Table S4 for the calculated DP values). We anticipate that this plot will be further discussed later (vide infra).

Next, we carried out similar experiments using CP **2** as the covalent feedstock. Mixtures of CP **2** and **4** mixed in a 1:2 ratio and prepared at increasing concentrations were treated with TFA. Also in this case, the addition of the acid caused an overturning from the *N*-aliphatic imines present in the cyclic and linear oligomers depicted in Figure 2B into the *N*-aromatic imines found in the SP hereafter referred to as SP-β = [**7**·**5**·2H^+^]_*n*_, which features the extensive coordination of the ammonium heads of **5**·2H^+^ into the crown-ether moieties of monomer **7** (see trace *d* in Figure 3B). Also with this system, 2D-DOSY experiments gave convincing evidence of the formation of SP-β. The diffusion coefficient related to all the signals of **5**·2H^+^ exactly coincides with *N*-aromatic imine functions (related to **7**) installed into the isophthalaldehyde backbone, further supporting the formation of SP-β (see Figure S58). Gratifyingly, the DP of SP-β increased with increasing concentration of **2** (Figure 3B; see Table S5 for the calculated DP values).

**Figure 5:**
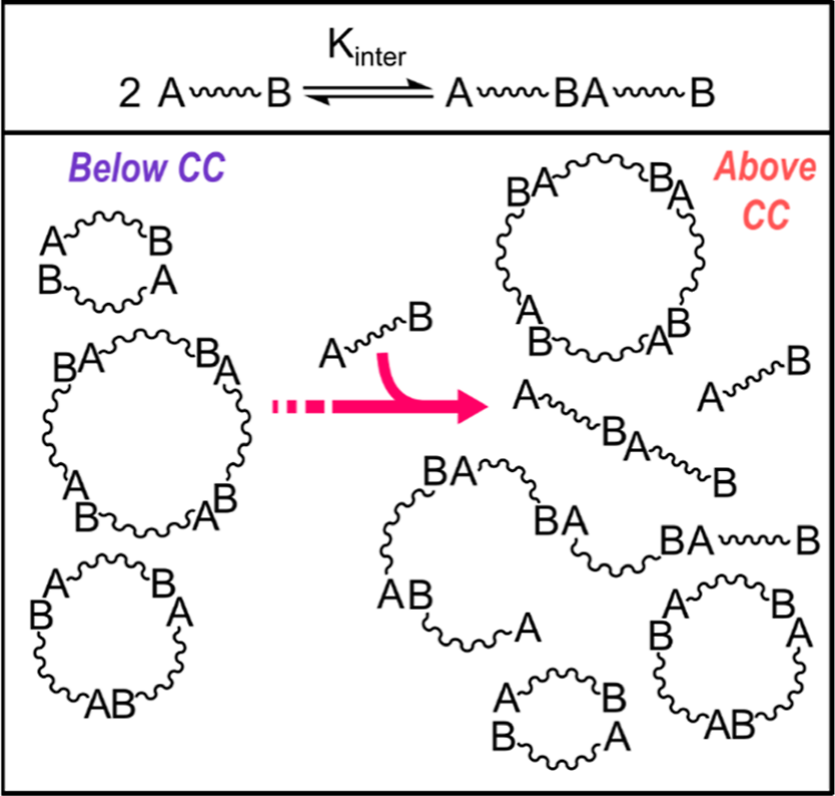
Behavior of a covalent or supramolecular polymer based on reversible interactions between monomers below and above the CC.

Prior to further discussing the profiles of the evolution of DP as a function of monomer concentration shown in Figure 3A (for SP-α) and Figure 3B (for SP-β), it is necessary to digress briefly into the behavior of polymerization systems in which monomers are held together by reversible (covalent or supramolecular) interactions. Previous literature^26,27^ revealed that the existence of a critical concentration (CC) of monomer units below which only cyclic species are present at equilibrium and above which only the linear fraction of the system will increase on increasing monomer concentration is a characteristic feature of such systems, which can be covalent or supramolecular in nature. This behavior is graphically presented in Figure 5. The higher the association constant between two monomers (*K*_inter_ in Figure 5), the narrower and more definite the value of the CC.^27^ Furthermore, the higher the strain energy present in the smaller cyclo-oligomers, the lower the value of CC.^27^ Above CC, the concentration of the cyclic fraction of the system reaches saturation; thus, only the concentration of the linear components will increase at higher monomer concentrations. Building on this explanation, we expect a rapid increase in the DP of SP-α and SP-β above the CC.

Looking back at the profile shown in Figure 3A, we estimated a CC^26,27^ of 145 mM for SP-α by considering the interpolation of the straight lines representing the two concentration domains^27^ (i.e., below and above CC) of the polymerization (dashed straight lines in Figure 3A). In the rocket domain where the linear polymer is expected to largely increase on increasing the monomer concentration,^26^ a DP of 120 is reached at 200 mM **1**; processing of the 2D-DOSY results provided a CC^26^ of 175 mM for the SP-β system (see the plot in Figure 3B), which is higher than that of SP-α. A DP of 100 is reached for the SP-β system when the monomer concentration is 300 mM to be compared with the DP of 120 obtained at a monomer concentration as low as 200 mM in the case of the previous system. Thus, the addition of TFA to “CCM **1** + **4**” and “CP **2** + **4**” revealed another interesting difference between the two systems. In the covalent assemblies, i.e., prior to the addition of TFA, the calixarene-based monomer displayed a much higher EM than the isophthalaldehyde-based monomer and related cyclo-oligomers, meaning that all of the material is engaged in the formation of CCM **1**. In stark contrast, the lower CC observed in the supramolecular analogues of the calixarene-based cyclo-oligomers compared to that of their isophthalaldehyde-based counterparts strongly suggests that the former have lower EMs than the latter.^26,27^ In other words, while in the covalent arrangement (in the absence of any added acid), the isophthalaldehyde-based system has a higher tendency toward polymerization than the calixarene-based one; in the supramolecular scenario (after addition of TFA), the opposite holds, with the calixarene-based system generating much longer polymers than the isophthalaldehyde-based one.

### Transient Supramolecular Polymers

Next, we explored the possibility of obtaining the supramolecular polymers described in the previous section in a transient fashion. Among the possible ACAs, tribromoacetic acid (TBA) seemed to be a convenient choice in terms of reactivity (reaction times) and waste production. The decarboxylation reaction is, in fact, carried out in CD_3_Cl/CD_3_OD 3:1, and the generation of CDBr_3_ as a waste product in large amounts is not expected to affect the reversibility of the system. Using trichloroacetic acid (TCA) as ACA would imply a number of drawbacks, including low reactivity and high risks of imine hydrolysis due to the highly hygroscopic nature of this reagent. The choice of TBA is also more practical as TCA is a deliquescent, hard-to-weight solid.

**Figure 6:**
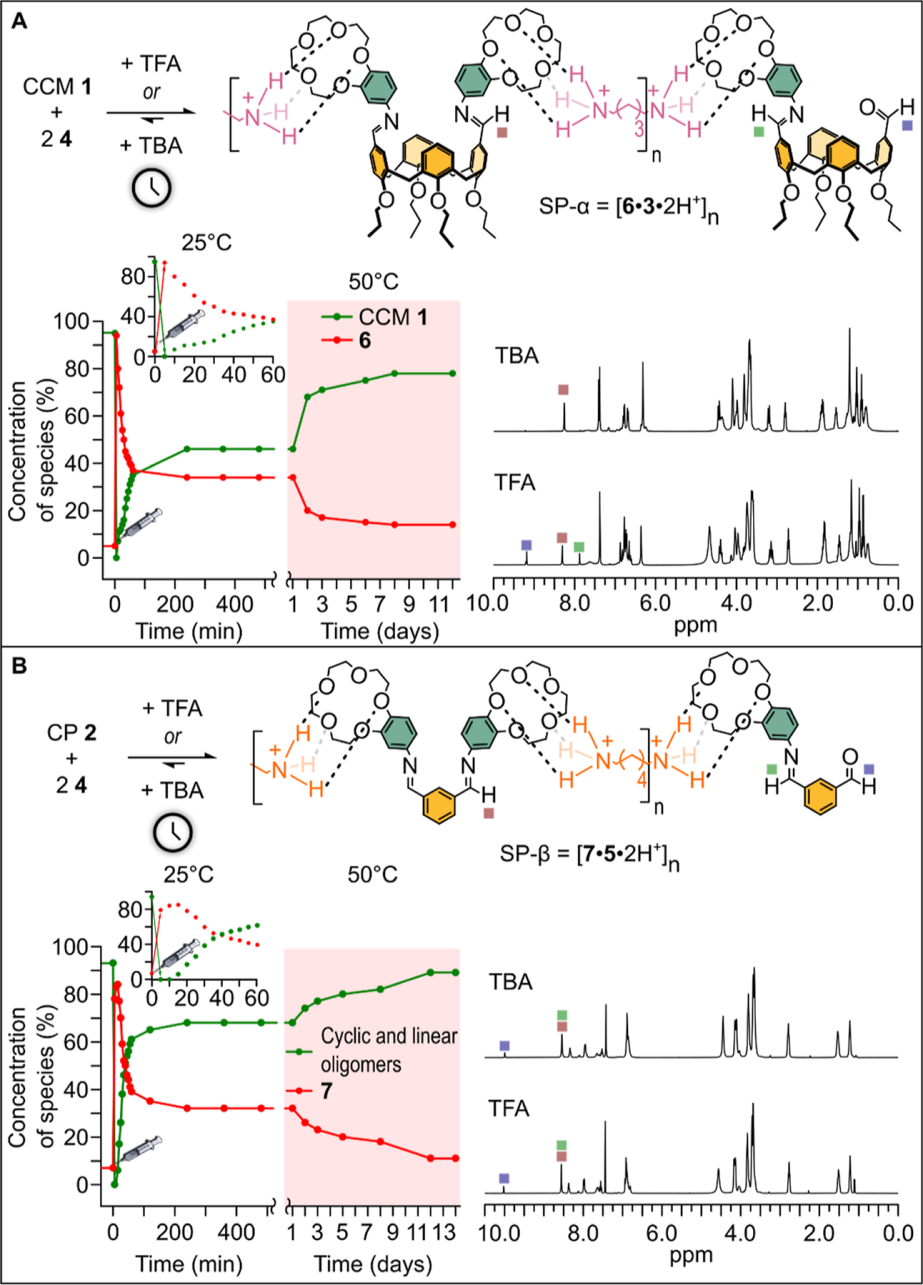
(A) ^1^H NMR spectra of the 1:2 mixtures of CCM **1** and **4** after addition of TFA (bottom trace) and TBA (top trace, the spectrum has been recorded immediately after the addition of TBA) and monitoring of CCM **1** and monomer **6** over the time (% data have been obtained from the relative integration of diagnostic imine signals). After 500 min, the temperature was raised to 50 °C (pink area) to allow reversion to the initial covalent material (the inset shows the initial phase of the reaction after addition of TBA at *t* = 0). (B) ^1^H NMR spectra of the 1:2 mixtures of CP **2** and **4** after addition of TFA (bottom trace) and TBA (top trace, the spectrum has been recorded immediately after the addition of TBA) and monitoring of CP **2** and **7** over the time (% data have been obtained from the relative integration of diagnostic imine signals). After 500 min, the temperature was raised to 50 °C (pink area) to allow reversion to the initial covalent material (the inset shows the initial phase of the reaction after addition of TBA at *t* = 0). The experimental error on % data for both (A,B) amounts to ca. 10%, the intrinsic error associated with the integration of NMR signals.

Thus, mixtures of CCM **1** and **4**, or CP **2** and **4**, in a 1:2 molar ratio were treated with TBA at 25 °C. After the addition of TBA, the starting covalently based materials **1** and **2** were mainly converted into the two supramolecular polymers SP-α and SP-β, respectively. This is demonstrated by the closely perfect matching of the ^1^H NMR spectra obtained after the addition of TFA and immediately after the addition of TBA (see Figure 6A,B). Although a direct characterization of the supramolecular polymer is made unfeasible by its transient character, a comparison of its ^1^H NMR spectral data with those obtained by using TFA does not leave any doubt about its formation. In fact, the main difference between the use of TFA and TBA is that CBr_3_CO_2_^–^, which is the conjugate base of TBA, is not stable and smoothly loses CO_2_ to be transformed into the anion CBr_3_^–^. The latter is a strong base that abstracts the proton (or, better said, deuteron) from the ammonium heads of the dications (conjugate diacids) of **3** and **5** to give CDBr_3_, inducing the system to reinstate the initial, equilibrated covalently based materials. Nevertheless, in both cases, the initial materials are not completely restored due to a kinetic issue. The concentration profiles shown in Figure 6A,B reveal that the concentrations of the interconverting species reach a plateau after ca. 220 min. The decarboxylation of TBA should be over at this time point since the back-transimination reaction^28,29^ becomes dramatically slow, a condition which is compatible with the absence of any acid. Under these conditions, dications of **3** or **5** must not be present anymore, and SP-α and SP-β must be disassembled. As a matter of fact, our experimental observation is that the starting covalent materials were not restored after 220 min. This means that the two imine-based dynamic libraries are found in a kinetic trap from which they cannot escape under the adopted conditions. In other words, the libraries are in a nonequilibrium metastable state (see Figures S71 and S72 for related ^1^H NMR monitoring). However, thermodynamic equilibrium, i.e., the initial covalently based material states, can be reached by warming up both libraries to 50 °C. This treatment restored CCM **1** after 8 days (Figure S73), while 12 days were needed to reobtain the mixture of cyclic and linear oligomers based on the isophthalaldehyde scaffold (Figure S74). Interestingly, an energy ratchet is realized in which, before warming, part of the free energy released from decarboxylation of CBr_3_CO_2_H is transiently diverted into the metastable state of the two nonequilibrium dynamic libraries.^17a,30^ However, the fact that the decarboxylation reaction is not coupled with back-transimination represents an unexpected complication for the system that cannot be easily solved by solvent changes. As a matter of fact, the choice of the 3:1 v/v CDCl_3_/CD_3_OD mixture was found to be the best compromise between reactivity/solubility^23^ of the library components and their propensity to associate through hydrogen bonding during the dissipative state. Finally, we point out that despite a potential competing hydrolysis of the imine, the system supports a second fueling cycle (shown with SP-β in Figure S75).

## Conclusions

To summarize, we have reported a new approach to trigger transient supramolecular polymerizations by combining the reversibility of dynamic covalent bonds and supramolecular interactions. Our strategy builds on the transformation of equilibrated dynamic combinatorial libraries of *N*-aliphatic imines and aromatic amines into dissipative dynamic combinatorial libraries of *N*-aromatic imines and protonated aliphatic amines, which is powered by the consumption of an activated carboxylic acid that serves as a chemical fuel. The higher basicity of aliphatic amines supports substitution of the amine component in the imine linkages under dissipative conditions. Treatment with the activated carboxylic acid produces aliphatic ammonium species that can interact with crown ether moieties embedded in the skeleton of the *N*-aromatic imines. The overall result is that the reservoir of covalent material of the dynamic combinatorial libraries is completely converted and temporarily stored in the form of a supramolecular polymer as long as the chemical fuel is present in solution. Fuel depletion reinstates the initial dynamic combinatorial libraries. Interestingly, the system behaves well both in the case of initial dynamic libraries composed mainly by one only imine component (case of CCM **1**) and in the case of initial dynamic libraries composed by a plethora of imine members (case of CP **2**). In particular, when the ACA is exhausted and the supramolecular polymers are disassembled, the high stability of CCM **1** drives a more rapid restoring of the initial composition than in the case of CP **2**.

Previously reported out-of-equilibrium supramolecular polymers relied on the activation of a “dormant” monomer via chemical modification that was induced by an external agent (the fuel). Here, the addition of the fuel brings about the selective activation of one component only (i.e., the aliphatic amines) of a feedstock comprising several building blocks and transiently triggers the exchange of the chemical connectivity of such building blocks. In other words, except for the external addition (or removal) of protons, the entire feedstock can be recycled and stored in either covalent or supramolecular form.

## Supporting Information Available

Experimental section, synthetic procedures and characterization (^1^H- and ^13^C NMR, ESI- and APCI–MS) of synthesized compounds, monitoring of transamination experiments at different concentrations by ^1^H NMR, SEC traces and analysis of polyimines obtained at different concentrations, and ^1^H NMR and 2D DOSY monitoring of acid- and fuel-induced transiminations (PDF)

## Abbreviations

DCL: dynamic combinatorial library
ACA: activated carboxylic acid
CCM: covalent cyclic monomer
CP: covalent polymer
SP: supramolecular polymer
^1^H NMR: proton nuclear magnetic resonance
^13^C NMR: carbon nuclear magnetic resonance
ESI–MS: electrospray ionization–mass spectrometry
APCI–MS: atmospheric pressure chemical ionization–mass spectrometry
EM: effective molarity
SEC: size exclusion chromatography
DP: polymerization degree
TFA: trifluoroacetic acid
2D-DOSY: two-dimensional diffusion-ordered spectroscopy
CC: critical concentration
TBA: tribromoacetic acid
TCA: trichloroacetic acid.
