# Supplementary material for: Imine-Based Transient Supramolecular Polymers
Source: J Am Chem Soc. 2025 Mar 19;147(13):11327–35. doi: 10.1021/jacs.5c00274 (PMC11969532; doi:10.1021/jacs.5c00274)
Supplement: Supplementary file 1 — ja5c00274_si_001.pdf [file ja5c00274_si_001.pdf]

Supporting Information

Imine-Based Transient Supramolecular Polymers

Gabriele Melchiorre, Lucia Visieri, Matteo Valentini, Roberta Cacciapaglia, Alessandro Casnati, Laura Baldini, José Augusto Berrocal,* and Stefano Di Stefano*

Experimental Section……………..………………………………………………………………S5
Instruments, methods and materials…………………………………………………………S5
Synthesis of CCM **1**………………………………..…………………………………………S6
^{1}H-NMR spectrum of CCM **1**……………………....…………………………………………S7
^{13}C-NMR spectrum of CCM **1**…………………….…………………..............................S7
ESI-MS analysis of CCM **1**……………………………..……………….................................S8
APCI-MS analysis of CCM **1**…………………………...……………….............................S9
Synthesis of CP **2** obtained at 100 mM monomer concentration……..…………..……………………S9
^{1}H-NMR ($CDCl_3$) of CP **2** obtained at 100 mM monomer concentration……..……...…...S10
^{1}H-NMR ($CDCl_3$ / CD_3OD 3:1) of CP **2** obtained at 100 mM monomer concentration….…S10
SEC analysis of CP **2** obtained at 100 mM monomer concentration……….………...……S11
ESI-MS analysis of CP **2** obtained at 100 mM monomer concentration……...…...………..S12
Synthesis of CP **2** at 500 mM monomer concentration…………………...………...…………………S13
^{1}H-NMR spectrum of CP **2** obtained at 500 mM monomer concentration……....…………S13
SEC analysis of CP **2** obtained at 500 mM monomer concentration…………………..………S14
ESI-MS analysis of CP **2** obtained at 500 mM monomer concentration………...……...…..S15
Synthesis of CP **2** at 1 M monomer concentration………..………………………...……………S15
^{1}H-NMR spectrum of CP **2** obtained at 1 M monomer concentration……...........................S16
SEC analysis of CP **2** obtained at 1 M monomer concentration……………...……………..S16
Transimination experiment between CCM **1** and **4**………………………..……………………S17
^{1}H-NMR spectrum of the transimination mixture of CCM **1** and **4**…….………..……..…...S17
Transimination experiment between CP **2** and **4**…………………………………..……………S18
^{1}H-NMR spectrum of the transimination mixture of CP **2** and **4**………....……………………..S18
ESI-MS analysis of the transimination mixture of CP **2** and **4**…………...….………..........S19
APCI-MS analysis of the transimination mixture of CP **2** and **4**……..…...........................S19
ESI-MS analysis of **4**………………...S20
SEC analysis of the transimination mixture of CP **2** and **4**…………….……..………………S21
Acid-induced transimination of 200 mM CCM **1** and 400 mM **4**, in a 400 mM TFA solution……...S22
^{1}H-NMR spectrum of a 200 mM CCM **1**, 400 mM **4**, and 400 mM TFA solution……......…S22
DOSY spectrum (Bayesian mode) of a 200 mM CCM **1**, 400 mM **4**, and 400 mM TFA solution……………………..……………………...………………………………S23

DOSY spectrum (Peak fit mode) of a 200 mM CCM **1**, 400 mM 4, and 400 mM TFA solution ……………………………………………………..………………………………S23

Acid-induced transimination of 175 mM CCM **1** and 350 mM **4**, in a 350 mM TFA solution……..S24

^{1}H-NMR spectrum of a 175 mM CCM **1**, 350 mM **4**, and 350 mM TFA solution…………..S24

DOSY spectrum (Bayesian mode) of a 175 mM CCM **1**, 350 mM **4**, and 350 mM TFA solution ……………………………………………………………..……………………S25

DOSY spectrum (Peak fit mode) of a 175 mM CCM **1**, 350 mM 4, and 350 mM TFA solution ……………………………………………………………………………………..S25

Acid-induced transimination of 150 mM CCM **1** and 300 mM **4**, in a 300 mM TFA solution……...S26

^{1}H-NMR spectrum of a 150 mM CCM **1**, 300 mM **4**, and 300 mM TFA solution…………..S26

DOSY spectrum (Bayesian mode) of a 150 mM CCM **1**, 300 mM **4**, and 300 mM TFA solution………………………………………………………………………………S27

DOSY spectrum (Peak fit mode) of a 150 mM CCM **1**, 300 mM 4, and 300 mM TFA solution………………………………………………………………………………S27

Acid-induced transimination of 100 mM CCM **1** and 200 mM **4**, in a 200 mM TFA solution……...S28

^{1}H-NMR spectrum of a 100 mM CCM **1**, 200 mM **4**, and 200 mM TFA solution…………..S28

DOSY spectrum (Bayesian mode) of a 100 mM CCM **1**, 200 mM **4**, and 200 mM TFA solution ……………………………………………………………………………………..S29

DOSY spectrum (Peak fit mode) of a 100 mM CCM **1**, 200 mM 4, and 200 mM TFA solution……………………………………………………………………...…..S29

Acid-induced transimination of 50 mM CCM **1** and 100 mM **4**, in a 100 mM TFA solution……….S30

^{1}H-NMR spectrum of a 50 mM CCM **1**, 100 mM **4**, and 100 mM TFA solution……………S30

DOSY spectrum (Bayesian mode) of a 50 mM CCM **1**, 100 mM **4**, and 100 mM TFA solution ……………………………………………………………………………………..S31

DOSY spectrum (Peak fit mode) of a 50 mM CCM **1**, 100 mM 4, and 100 mM TFA solution ……………………………………………………………………………………..S31

Synthesis of monomer **6**…………………………………………………………………………S32

^{1}H-NMR spectrum of monomer **6**…………………………………………………………S32

DOSY spectrum (Bayesian mode) of 50 mM monomer **6**…………………….………………S33

DOSY spectrum (Peak fit mode) of 50 mM monomer **6**……………………..……………..S33

Degree of polymerization (DP) of SP-α……………………………….……………………….S34

^{1}H-NMR spectra of **3**, **3**•2H^{+}, **3**•2H^{+} with **4**, and of a 1:2:2 mixture of CCM **1**, **4**, and TFA………...S35

Acid-induced transimination of 300 mM CP **2** and 600 mM **4**, in a 600 mM TFA solution………...S36

^{1}H-NMR spectrum of a 300 mM CP **2**, 600 mM **4**, and 600 mM TFA solution…………….S36

DOSY spectrum (Bayesian mode) of a 300 mM CP **2**, 600 mM **4**, and 600 mM TFA solution ……………………………………………………………………………………..S37

DOSY spectrum (Peak fit mode) of a 300 mM CP **2**, 600 mM **4**, and 600 mM TFA solution ……………………………………………………………………………………..S37

Acid-induced transimination of 250 mM CP **2** and 500 mM **4**, in a 500 mM TFA solution………...S38

^{1}H-NMR spectrum of a 250 mM CP **2**, 500 mM **4**, and 500 mM TFA solution……..………S38

DOSY spectrum (Bayesian mode) of a 250 mM CP **2**, 500 mM **4**, and 500 mM TFA solution ……………………………………………………………………………………..S39

DOSY spectrum (Peak fit mode) of a 250 mM CP **2**, 500 mM 4, and 500 mM TFA solution ……………………………………………………………………………………..S39

Acid-induced transimination of 200 mM CP **2** and 400 mM **4**, in a 400 mM TFA solution………...S40

^{1}H-NMR spectrum of a 200 mM CP **2**, 400 mM **4**, and 400 mM TFA solution……………..S40

DOSY spectrum (Bayesian mode) of a 200 mM CP **2**, 400 mM **4**, and 400 mM TFA solution ……………………………………………………………………………………..S41

DOSY spectrum (Peak fit mode) of a 200 mM CP **2**, 400 mM 4, and 400 mM TFA solution ……………………………………………………………………………………..S41

Acid-induced transimination of 175 mM CP **2** and 350 mM **4**, in a 350 mM TFA solution………...S42

^{1}H-NMR spectrum of a 175 mM CP **2**, 350 mM **4**, and 350 mM TFA solution……………..S42

DOSY spectrum (Bayesian mode) of a 175 mM CP **2**, 350 mM **4**, and 350 mM TFA solution ……………………………………………………………………………………..S43

DOSY spectrum (Peak fit mode) of a 175 mM CP **2**, 350 mM 4, and 350 mM TFA solution ……………………………………………………………………………………..S43

Acid-induced transimination of 150 mM CP **2** and 300 mM **4**, in a 300 mM TFA solution………...S44

^{1}H-NMR spectrum of a 150 mM CP **2**, 300 mM **4**, and 300 mM TFA solution…………..…S44

DOSY spectrum (Bayesian mode) of a 150 mM CP **2**, 300 mM **4**, and 300 mM TFA solution ……………………………………………………………………………………..S45

DOSY spectrum (Peak fit mode) of a 150 mM CP **2**, 300 mM 4, and 300 mM TFA solution ……………………………………………………………………………………..S45

Acid-induced transimination of 100 mM CP **2** and 200 mM **4**, in a 200 mM TFA solution………...S46

^{1}H-NMR spectrum of a 100 mM CP **2**, 200 mM **4**, and 200 mM TFA solution…………..…S46

DOSY spectrum (Bayesian mode) of a 100 mM CP **2**, 200 mM **4**, and 200 mM TFA solution……………………………………………………………………………S47

DOSY spectrum (Peak fit mode) of a 100 mM CP **2**, 200 mM 4, and 200 mM TFA solution …………………………………………………………………………………..……S47

Acid-induced transimination of 50 mM CP **2** and 100 mM **4**, in a 100 mM TFA solution…………S48

^{1}H-NMR spectrum of a 50 mM CP **2**, 100 mM **4**, and 100 mM TFA solution…………….....S48

DOSY spectrum (Bayesian mode) of a 50 mM CP **2**, 100 mM **4**, and 100 mM TFA solution …………………………………………………………………………………..S49

DOSY spectrum (Peak fit mode) of a 50 mM CP **2**, 100 mM 4, and 100 mM TFA solution...S49

Acid-induced transimination of 25 mM CP **2** and 50 mM **4**, in a 50 mM TFA solution…………….S50

^{1}H-NMR spectrum of a 25 mM CP **2**, 50 mM **4**, and 50 mM TFA solution…………………S50

DOSY spectrum (Bayesian mode) of a 25 mM CP **2**, 50 mM **4**, and 50 mM TFA solution…S51

DOSY spectrum (Peak fit mode) of a 25 mM CP **2**, 50 mM 4, and 50 mM TFA solution…...S51

Synthesis of Monomer **7**……………………………..………………………………S52

^{1}H-NMR spectrum of monomer **7**…………………………………………………S52

Degree of polymerization (DP) of SP-β……………………………………………….…...S53

Sample preparation of monomer **7** DOSY experiment…....…………….……….…………S53

DOSY spectrum (Bayesian mode) of monomer **7**……………………………………………S53

DOSY spectrum (Peak fit mode) of monomer **7**………………………….……………..…..S54

^{1}H-NMR spectra of **5**, **5**•2H^{+}, **5**•2H^{+} with **4**, and a 1:2:2 mixture of CP **2**, **4**. and TFA…………….....S55

^{1}H-NMR monitoring of a 1:2 mixture of CCM **1** and **4** before and after TBA addition…………….S56

^{1}H-NMR monitoring of a 1:2 mixture of CP **2** and **4** before and after TBA addition……………….S57

Composition comparison for a CCM **1** / **4** mixture 1 : 2 before and after TBA addition…………..S58

Composition comparison for a CP **2** / **4** mixture 1 : 2 before and after TBA addition……………..S59

^{1}H-NMR monitoring of a 1:2 mixture of CP **2** and **4** before and after refuelling experiment……….S60

Bibliography………...…………………………..……………………………………S61

Experimental section

Instruments, methods and materials

^{1}H-NMR and ^{13}C-NMR monodimensional spectra were recorded at room temperature on either a Bruker Avance II 400 MHz or a Bruker Avance III 400 MHz spectrometer. DOSY spectra were recorded on a Bruker Avance II 400 MHz at 25 °C. The ^{1}H-NMR spectra were internally referenced to the residual proton signal of the solvent at 7.26 ppm in $CDCl_3$ and at 7.41 ppm in the $CDCl_3$ / CD_3OD 3:1 mixture. ^{13}C-NMR spectra were internally referenced to the ^{13}C-signal of $CDCl_3$, set at 77.36 ppm in $CDCl_3$ / CD_3OD 3:1 mixture.

Mass spectrometric analyses were performed on an Agilent 1200 series HPLC system equipped with a diode array detector (DAD) and a simple quadrupole LC/MS 6130 mass detector. The output signal was monitored and processed using the Agilent Chemstation software Open Lab. For sample preparation, 1 mg of sample was dissolved in 1 mL of chloroform.

Size exclusion chromatography (SEC) measurements on poly(imine) substrates were carried out using an Agilent 1200 series HPLC system, equipped with PSS SDV Analytical linear M SEC column (8 × 300 mm; 5 μm particle size) in tetrahydrofuran (THF, HPLC grade) at 25 °C, at a flow rate of 1 mL/min. Samples were analysed at a concentration of 1 mg/mL after filtration through a 0.45 μm pore-size membrane. Mn, Mw, and Đ (Mw/Mn) values were derived from the RI signal by a calibration curve based on polystyrene standards (PS from Polymer Standards Service) for polymer analysis. SEC samples were prepared by dissolving 2 mg of substrate in 2 mL of THF and filtering the solution through a 0.45 μm pore-size membrane.

All reagents and solvents were purchased from Sigma Aldrich or TCI-chemicals, and 5,17-diformyl-25,26,27,28-tetrapropoxycalix[4]arene was synthesized as previously described.[1] Deuterated chloroform and deuterated methanol were dried on 3 Å molecular sieves.

Synthesis of CCM 1

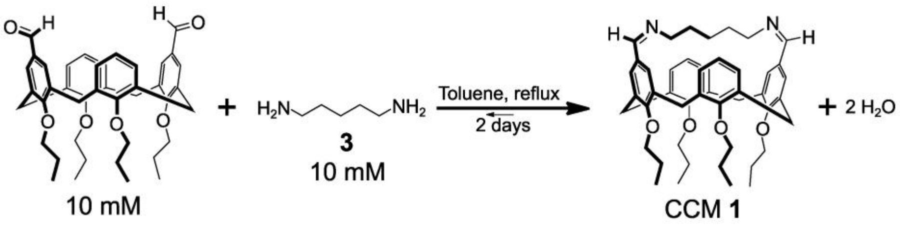

Figure S1. Synthesis of CCM **1**.

Following a procedure adopted for the synthesis of an analogous derivative,[2] in a 100 mL round bottom flask 285 mg of 5,17-diformyl-25,26,27,28-tetrapropoxycalix[4]arene (0.44 mmol) were dissolved in 44 mL of anhydrous toluene. Then, 52 μL (0.44 mmol) of **3** were added to the solution, which was refluxed and stirred for 2 days. After cooling the mixture to room temperature, the solvent was removed and CCM **1** was obtained with quantitative yield. CCM **1** was then characterized by ^{1}H-NMR, ^{13}C-NMR, and ESI- and APCI-MS. **^{1}H-NMR** (400 MHz, $CDCl_3$ / CD_3OD 3:1) δ: 7.47 (s, 2H), 7.18 (dd, J = 7.4, 1.8 Hz, 4H), 6.95 (td, J = 7.4, 1.9 Hz, 2H), 6.56 (d, J = 1.7 Hz, 4H), 4.46 (dd, J = 13.1, 1.8 Hz, 4H), 4.09 (ddd, J = 10.4, 6.9, 3.1 Hz, 4H), 3.66 (td, J = 6.9, 1.8 Hz, 4H), 3.38 – 3.32 (m, 4H), 3.18 (d, J = 13.2 Hz, 4H), 2.07 – 1.82 (m, 8H), 1.49 (dt, J = 11.4, 5.6 Hz, 4H), 1.13 – 1.06 (m, 6H), 0.89 (td, J = 7.5, 1.8 Hz, 6H), 0.62 (dd, J = 14.5, 7.5 Hz, 2H). **^{13}C-NMR** (100 MHz, $CDCl_3$ / CD_3OD 3:1) δ: 163.14, 158.31, 157.72, 136.56, 133.97, 129.44, 129.35, 128.03, 122.75, 77.60, 76.75, 60.60, 31.13, 29.80, 28.73, 23.72, 23.39, 23.08, 10.90, 9.88. **(ESI)**: $[M + H]^+$ (M = $H_{47}C_{58}N_2O_4$): m/z calculated: 715.4; found: 715.3; $[M + Na]^+$ = 738.4; found: 737.3 **(APCI)**: $[M + H]^+$ (M = $H_{47}C_{58}N_2O_4$): m/z calculated: 715.4; found: 715.3.

^{1}H-NMR spectrum of CCM 1

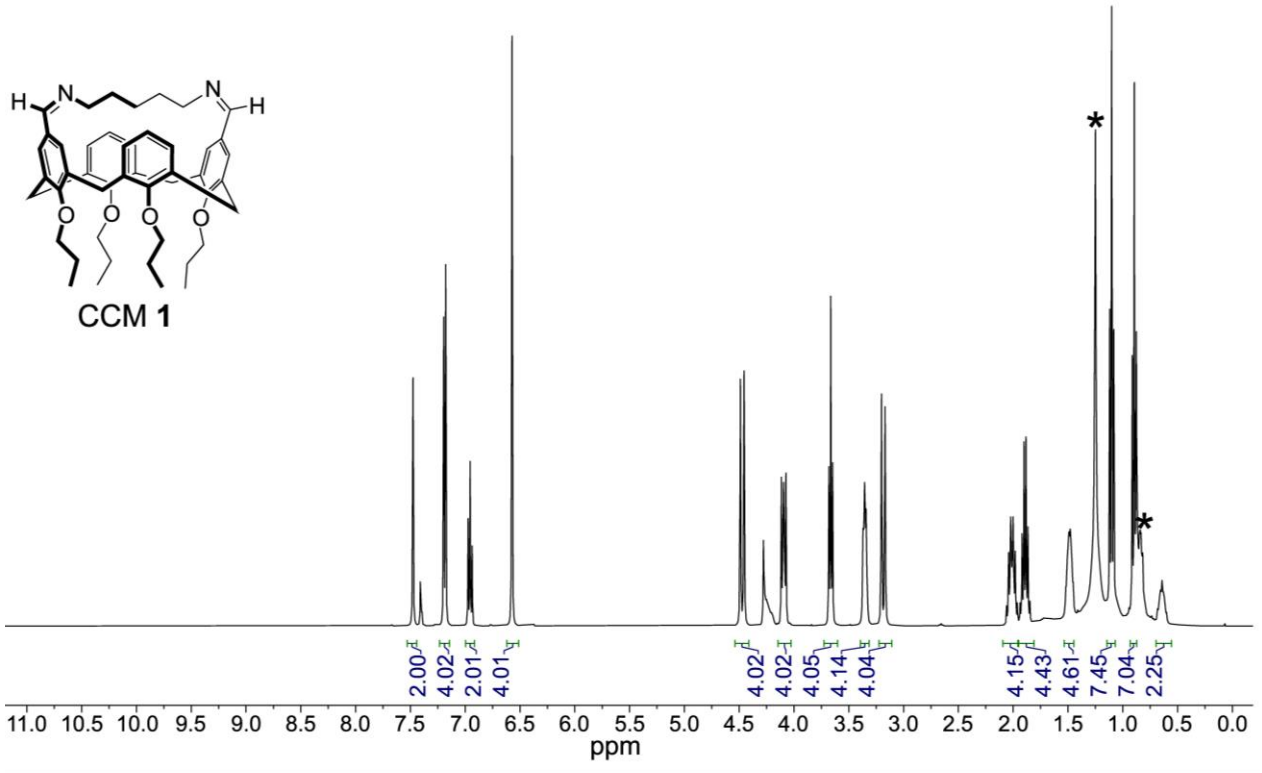

Figure S2. ^{1}H-NMR ($CDCl_3$ / CD_3OD 3:1) of **1**, (grease impurities marked with an asterisk).

^{13}C-NMR spectrum of CCM 1

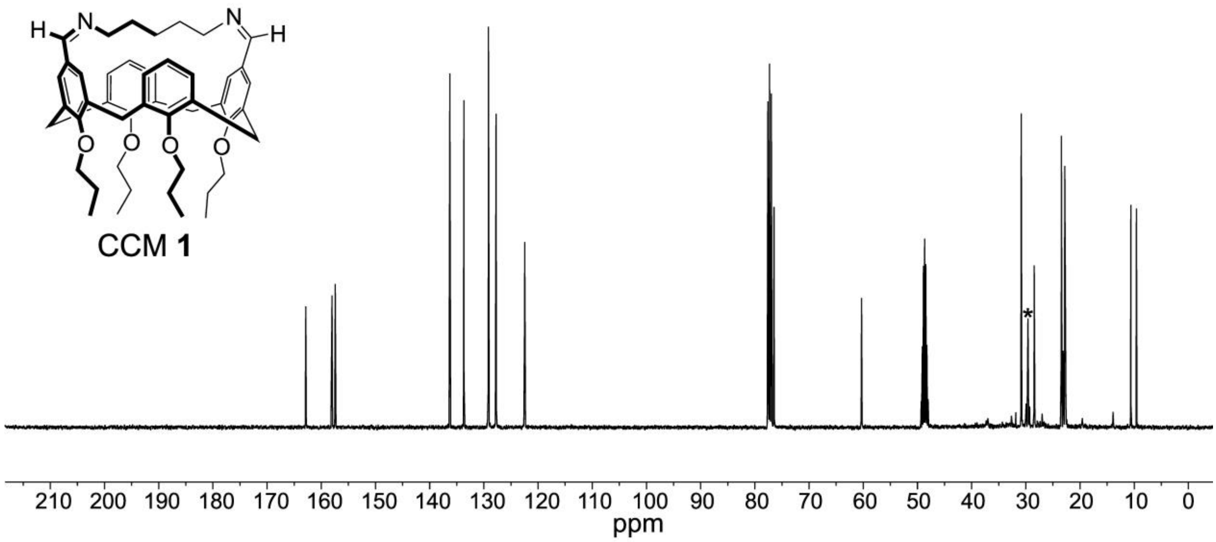

Figure S3. ^{13}C-NMR ($CDCl_3$ / CD_3OD 3:1) of CCM **1**, (grease impurities marked with an asterisk).

ESI-MS analysis of CCM 1

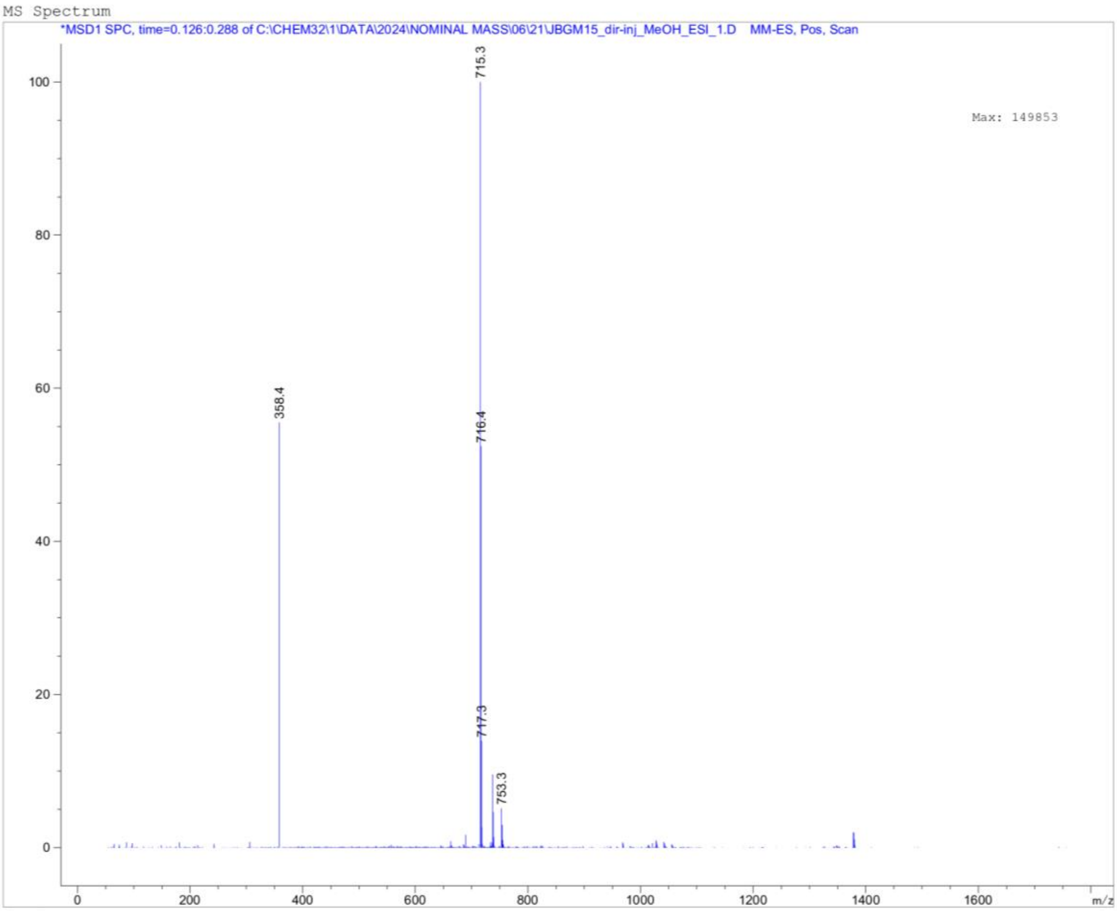

Figure S4. ESI-MS analysis of CCM **1**.

APCI-MS analysis of CCM 1

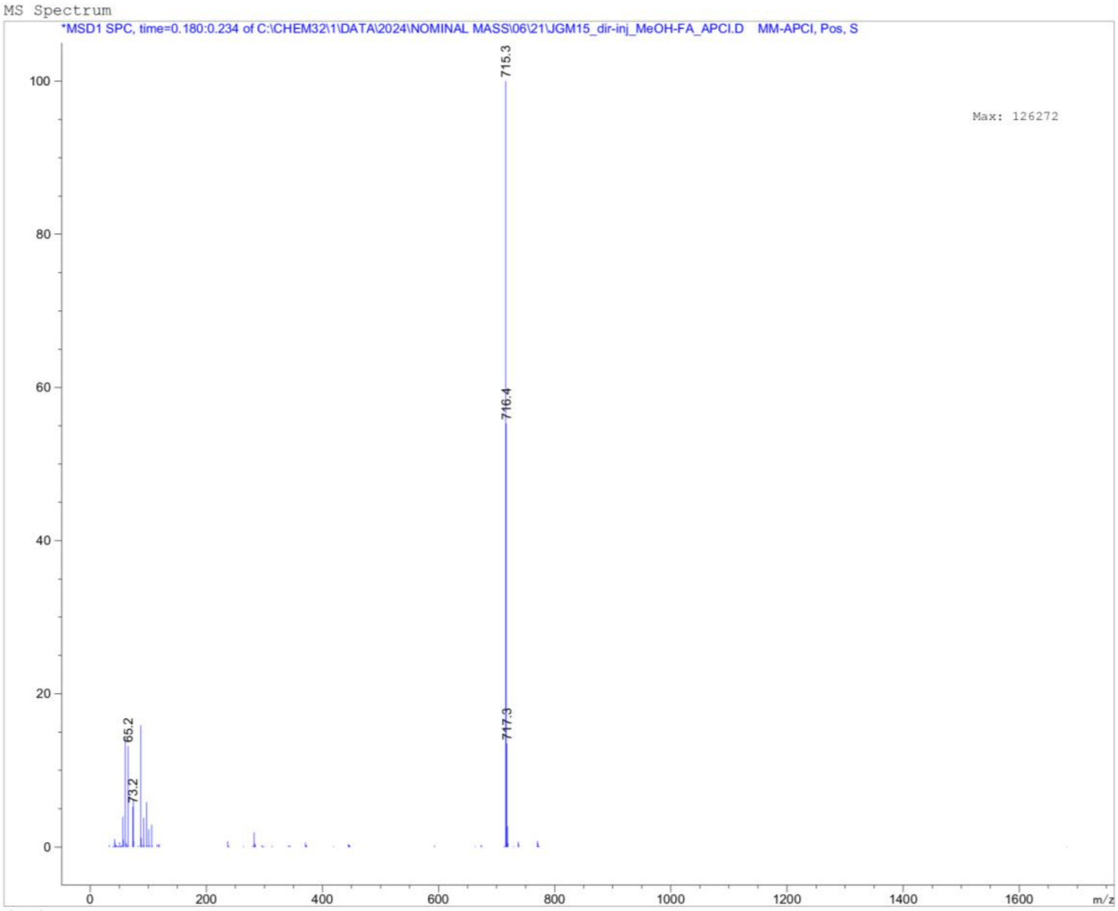

Figure S5. APCI-MS analysis of CCM **1**.

Synthesis of CP 2 at 100 mM monomer concentration

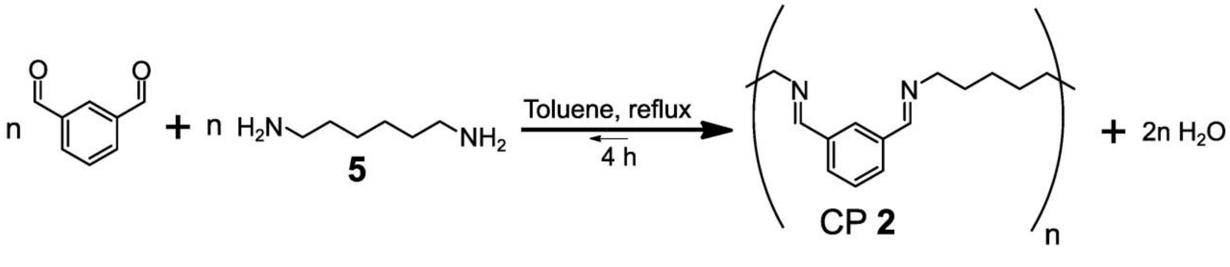

Figure S6. Synthesis of CP **2**.

In a 25 mL round bottom flask 87.2 mg of **5** (0.75 mmol) were dissolved in 7.5 mL of anhydrous toluene. Then, 101 mg (0.75 mmol) of isophthalaldehyde were added to the solution, which was refluxed under stirring for 4 hours. After cooling, the solvent was removed, and the resulting product was analysed by ^{1}H-NMR, SEC, and ESI-MS. ^{1}H NMR (400 MHz, $CDCl_3$) δ: 8.29 (s, 2H), 8.01 (s, 1H), 7.78 (dd, J = 7.7, 1.7 Hz, 2H), 7.43 (t, J = 7.7 Hz, 1H), 3.61 (t, J = 6.9 Hz, 4H), 1.71 (d, J = 8.0 Hz, 4H), 1.41 (s, 4H). ^{1}H NMR (400 MHz, CD_3OD) δ: δ 8.30 (s, 2H), 7.97 (t, J = 1.7 Hz, 1H), 7.79 (dd, J = 7.7, 1.7 Hz, 2H), 7.47 (t, J = 7.7 Hz, 1H), 3.64 – 3.56 (m, 4H), 1.71 (m, J = 11.0, 5.2 Hz, 4H), 1.41 (h, J = 3.6 Hz, 4H).

^{1}H-NMR ($CDCl_3$) of CP 2 obtained at 100 mM monomer concentration

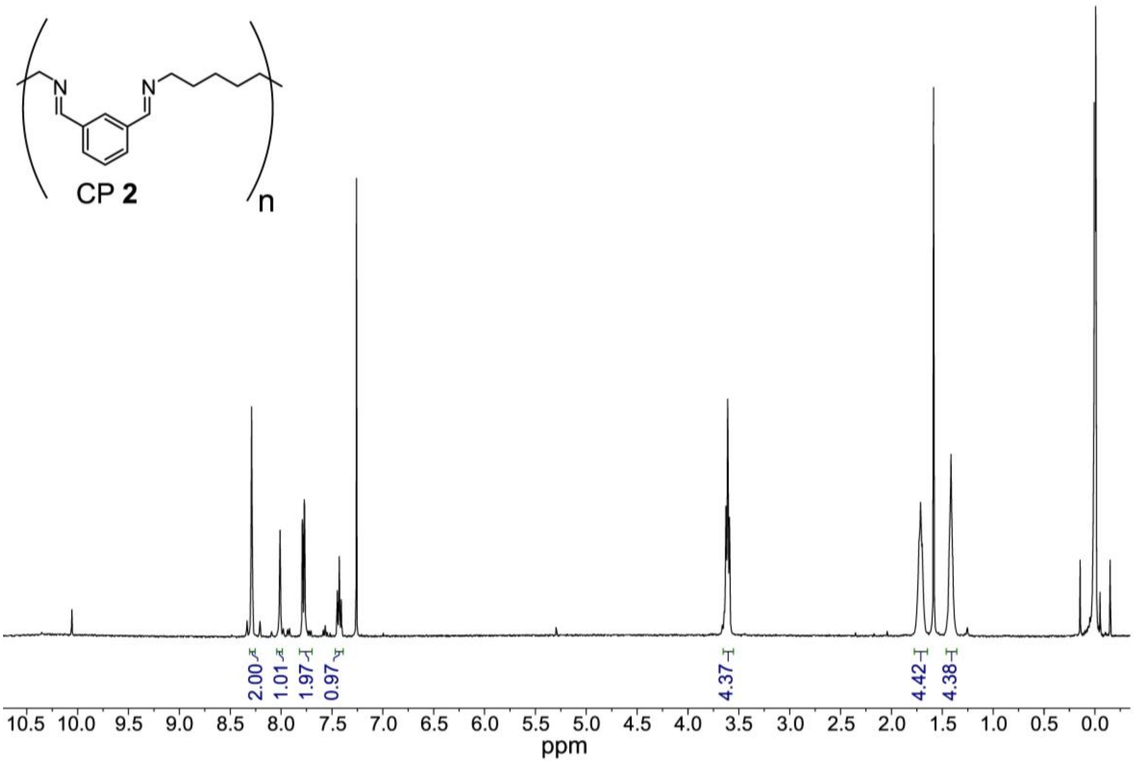

Figure S7. ^{1}H-NMR ($CDCl_3$) of CP **2** obtained from reaction at 100 mM monomer concentration.

^{1}H-NMR ($CDCl_3$ / CD_3OD 3:1) of CP 2 obtained at 100 mM monomer concentration

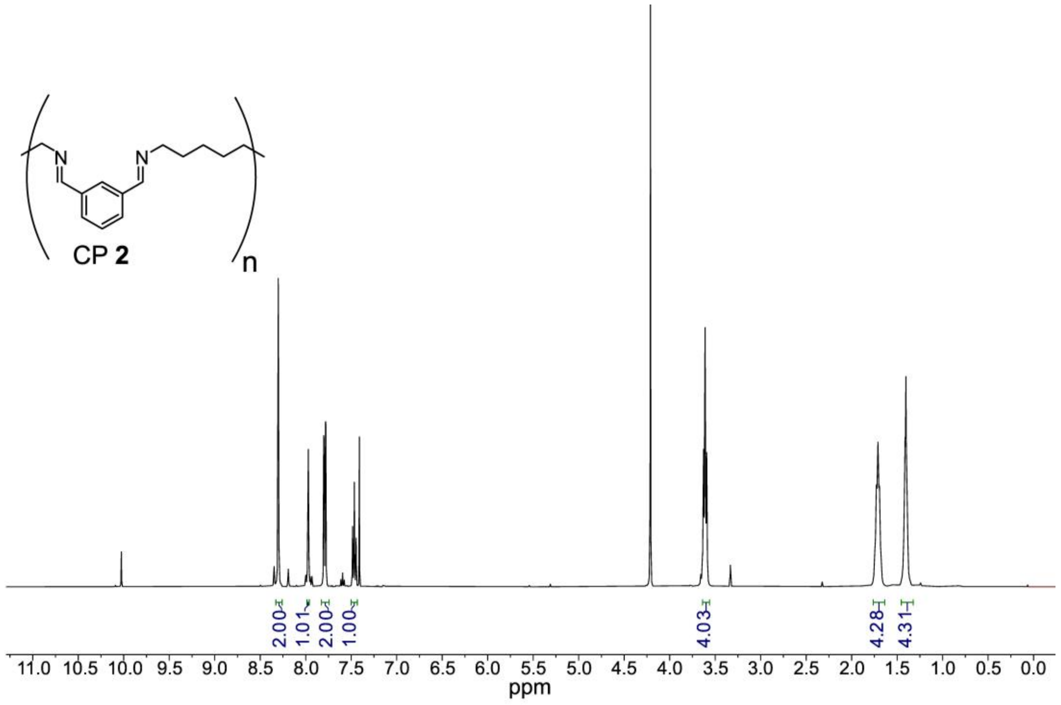

Figure S8. ^{1}H-NMR ($CDCl_3$ / CD_3OD 3:1) of CP **2** obtained from reaction at 100 mM monomer concentration.

SEC analysis of CP 2 obtained at 100 mM monomer concentration

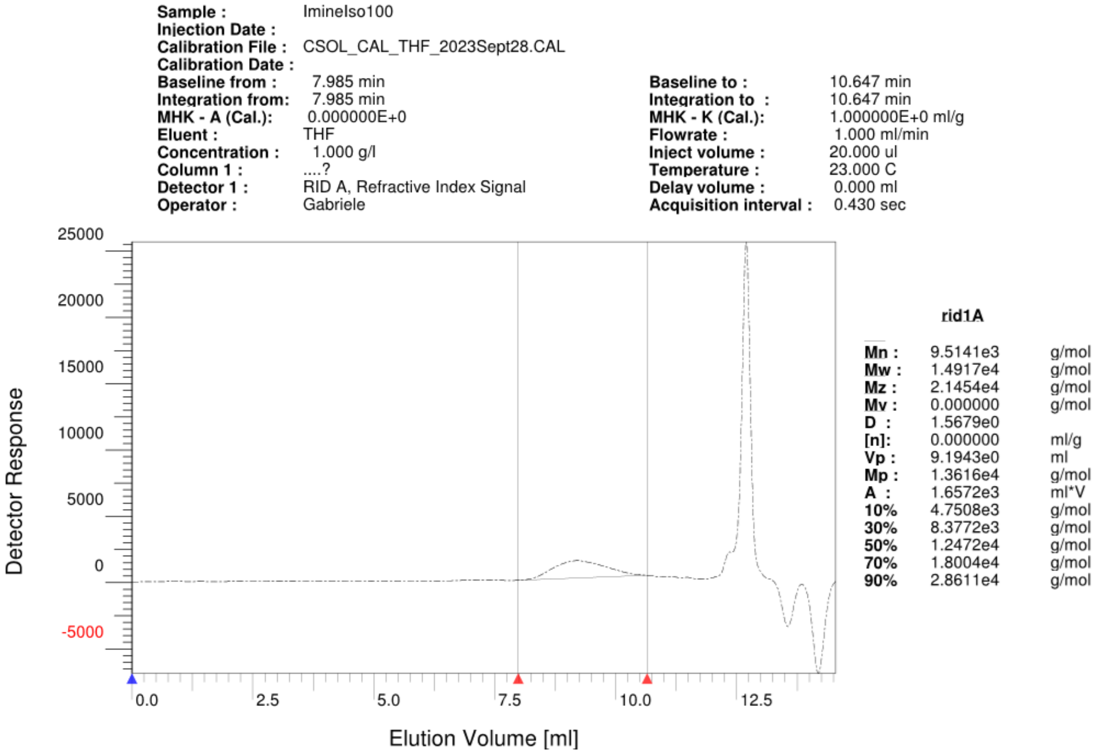

Figure S9. SEC analysis of CP **2** obtained at 100 mM monomer concentration.

The obtained $\overline{M_n}$ value is $9.51 \cdot 10^3$ g/mol. Considering imine C_1 (Figure S10, mass weight M_0 214.3 g/mol) as the repeating unit,

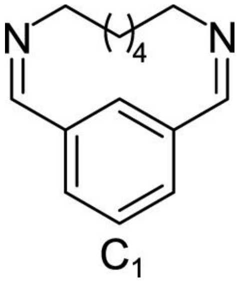

Figure S10. Repeating unit for CP **2**.

for CP **2** obtained at 100 mM monomer concentration, the average degree of polymerization (DP_n) is 44, as derived from Equation S1

$$DP_n = \frac{\overline{M_n}}{M_0} = 44$$

Equation S1

ESI-MS analysis of CP 2 synthesized at 100 mM monomer concentration

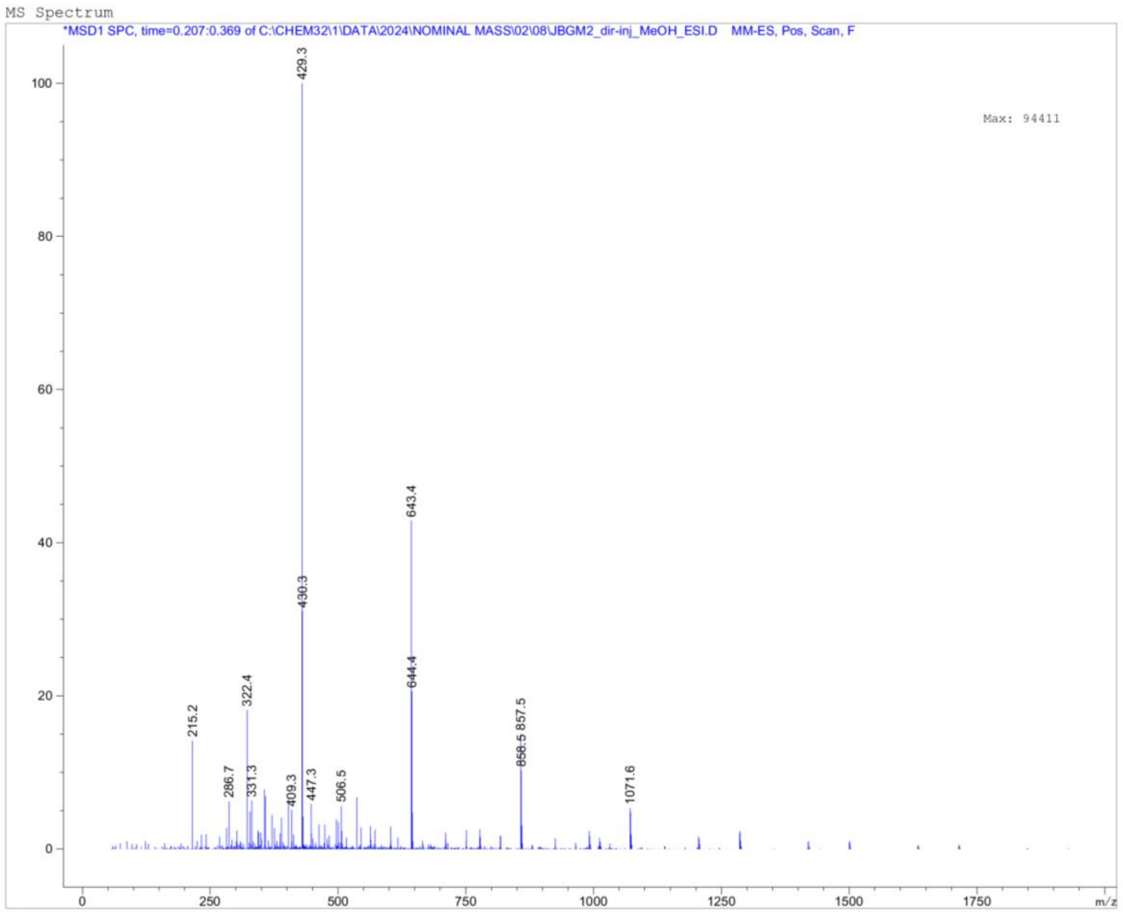

Figure S11. ESI-MS analysis of CP **2** obtained at 100 mM monomer concentration.

The *m*/*z* values obtained are assigned to charged adduct of imine-based macrocyclic species obtained in the condensation between isophthalaldehyde and **5**, as reported in Table S1.

Table S1. *m*/*z* values from ESI-MS analysis of CP **2** obtained at 100 mM monomer concentration, assigned to the corresponding macrocycles.

Imine	*m*/*z* for $[M+H^+]$
Monomeric macrocycle C_1	215.2
Dimeric macrocycle C_2	429.3
Trimeric macrocycle C_3	643.4
Tetrameric macrocycle C_4	857.5
Pentameric macrocycle C_5	1071.6

Synthesis of CP 2 at 500 mM monomer concentration

In a 25 mL round bottom flask 436 mg of **5** (3.75 mmol) were dissolved in 7.5 mL of anhydrous toluene. Then, 503 mg (3.75 mmol) of isophthalaldehyde were added to the solution, which was refluxed under stirring for 4 hours. After cooling, the solvent was removed by vacuum, and the resulting product was analysed by ^{1}H-NMR, SEC, and ESI-MS.

^{1}H NMR (400 MHz, $CDCl_3$) δ: 8.28 (d, J = 3.7 Hz, 2H), 8.06 – 7.89 (m, 1H), 7.81 – 7.68 (m, 2H), 7.42 (td, J = 7.7, 3.0 Hz, 1H), 3.68 – 3.57 (m, 4H), 1.71 (dp, J = 11.7, 6.3 Hz, 4H), 1.47 – 1.35 (m, 4H).

^{1}H-NMR spectrum of CP 2 synthesized at 500 mM monomer concentration

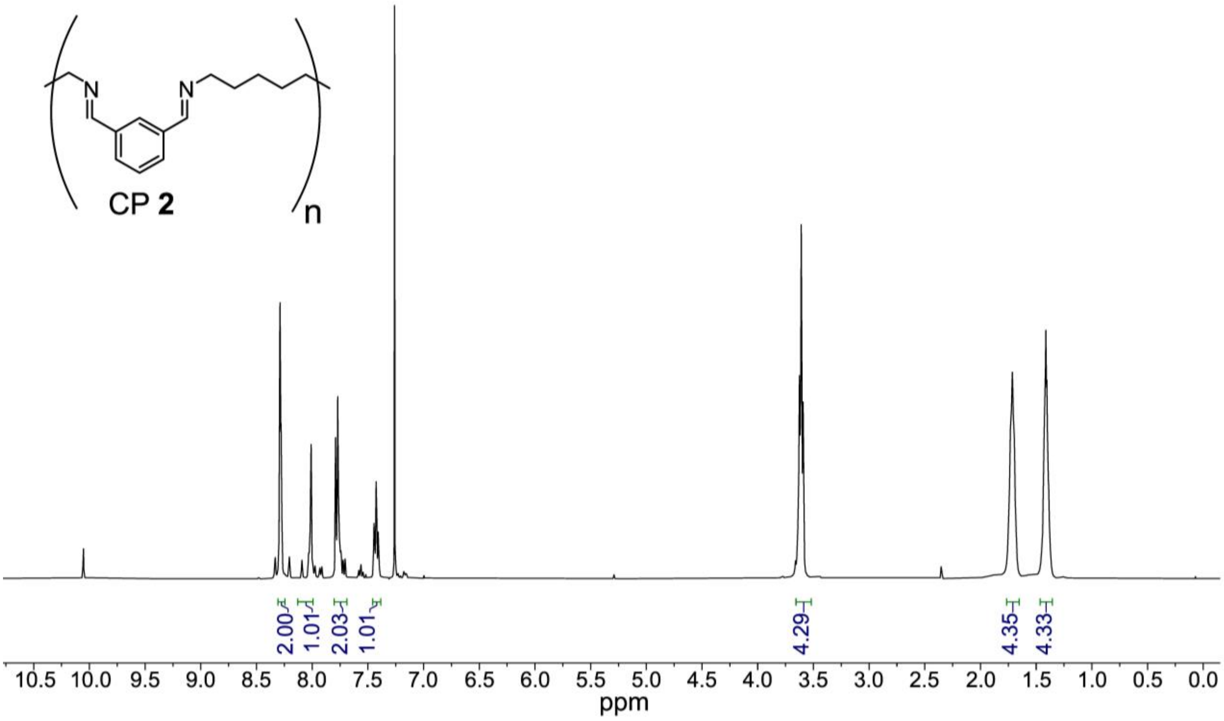

Figure S12. ^{1}H-NMR ($CDCl_3$) of CP **2** obtained at 500 mM monomer concentration.

SEC analysis of CP 2 obtained at 500 mM monomer concentration

**Sample :**	Imine500I		
**Injection Date :**			
**Calibration File :**	CSOL_CAL_THF_2023Sept28.CAL		
**Calibration Date :**			
**Baseline from :**	7.700 min	**Baseline to :**	10.663 min
**Integration from:**	7.700 min	**Integration to :**	10.663 min
**MHK - A (Cal.):**	0.000000E+0	**MHK - K (Cal.):**	1.000000E+0 ml/g
**Eluent :**	THF	**Flowrate :**	1.000 ml/min
**Concentration :**	0.000 g/l	**Inject volume :**	20.000 ul
**Column 1 :**	n.a.	**Temperature :**	23.000 C
**Detector 1 :**	RID A, Refractive Index Signal	**Delay volume :**	0.000 ml
**Operator :**	Gabriele	**Acquisition interval :**	0.430 sec

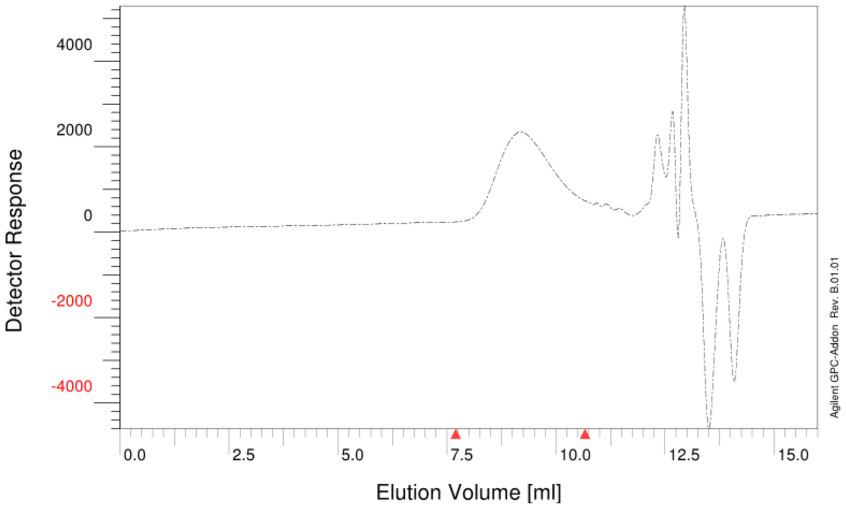

rid1A

**Mn :**	9.3144e3	g/mol
**Mw :**	1.5072e4	g/mol
**Mz :**	2.2099e4	g/mol
**Mv :**	0.000000	g/mol
**D :**	1.6181e0	
**[n]:**	0.000000	ml/g
**Vp :**	9.1727e0	ml
**Mp :**	1.4043e4	g/mol
**A :**	2.4280e3	ml*V
**10%**	4.5568e3	g/mol
**30%**	8.2847e3	g/mol
**50%**	1.2477e4	g/mol
**70%**	1.8191e4	g/mol
**90%**	2.9271e4	g/mol

Figure S13. SEC analysis of CP **2** obtained at 500 mM monomer concentration. For this poly(imine) $\overline{DP_n}$ 43 is obtained.

ESI-MS analysis of CP 2 obtained at 500 mM monomer concentration

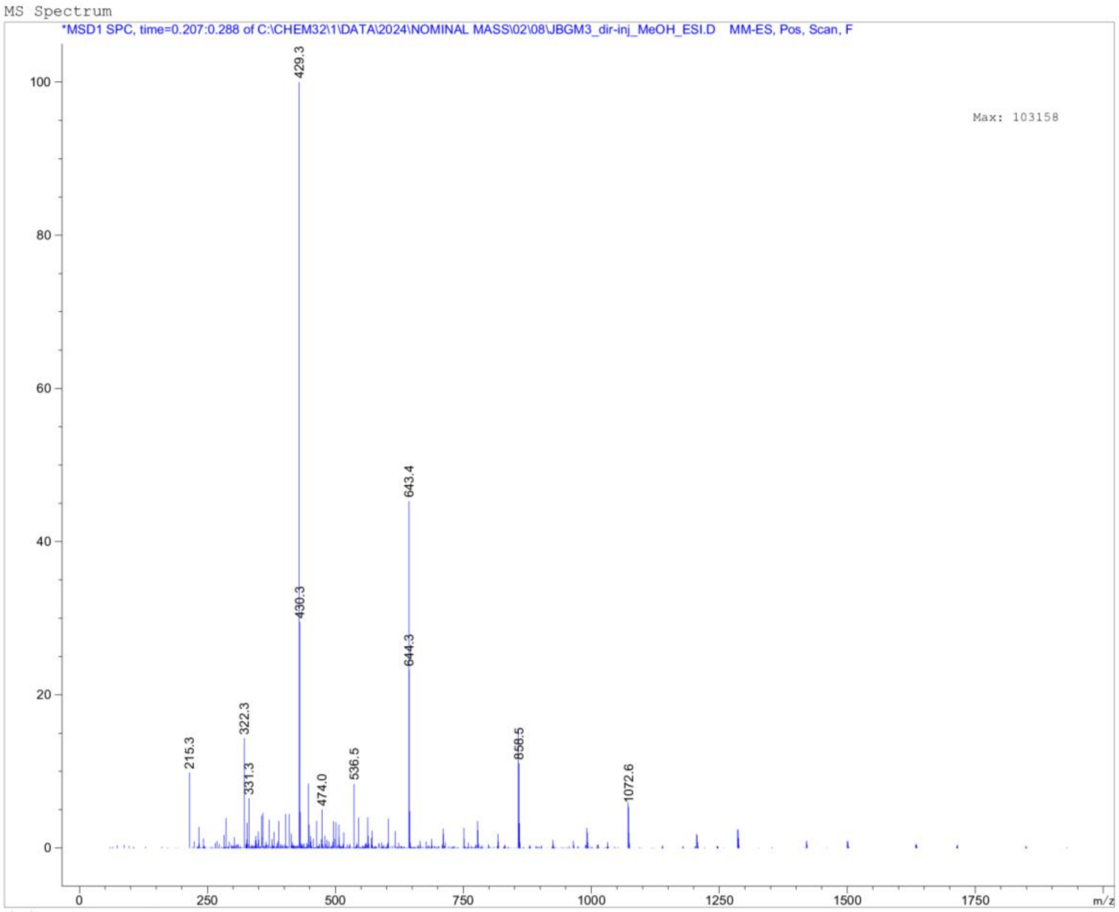

Figure S14. ESI-MS analysis of CP **2** obtained at 500 mM monomer concentration.

Synthesis of CP 2 at 1 M monomer concentration

In a 25 mL round bottom flask 581 mg of **5** (5.00 mmol) were dissolved in 5 mL of anhydrous toluene. Then, 671 mg (5.00 mmol) of isophthalaldehyde were added to the solution, which was refluxed under stirring for 4 hours. After cooling, the solvent was removed, and the resulting product was analysed by ^{1}H-NMR and SEC. ^{1}H NMR (400 MHz, $CDCl_3$) δ: 8.28 (t, J = 2.7 Hz, 2H), 8.11 – 7.99 (m, 1H), 7.82 – 7.68 (m, 2H), 7.42 (td, J = 7.6, 2.5 Hz, 1H), 3.68 – 3.55 (m, 4H), 1.76 – 1.66 (m, 4H), 1.40 (tt, J = 9.9, 5.4 Hz, 4H).

^{1}H-NMR spectrum of CP 2 obtained at 1 M monomer concentration

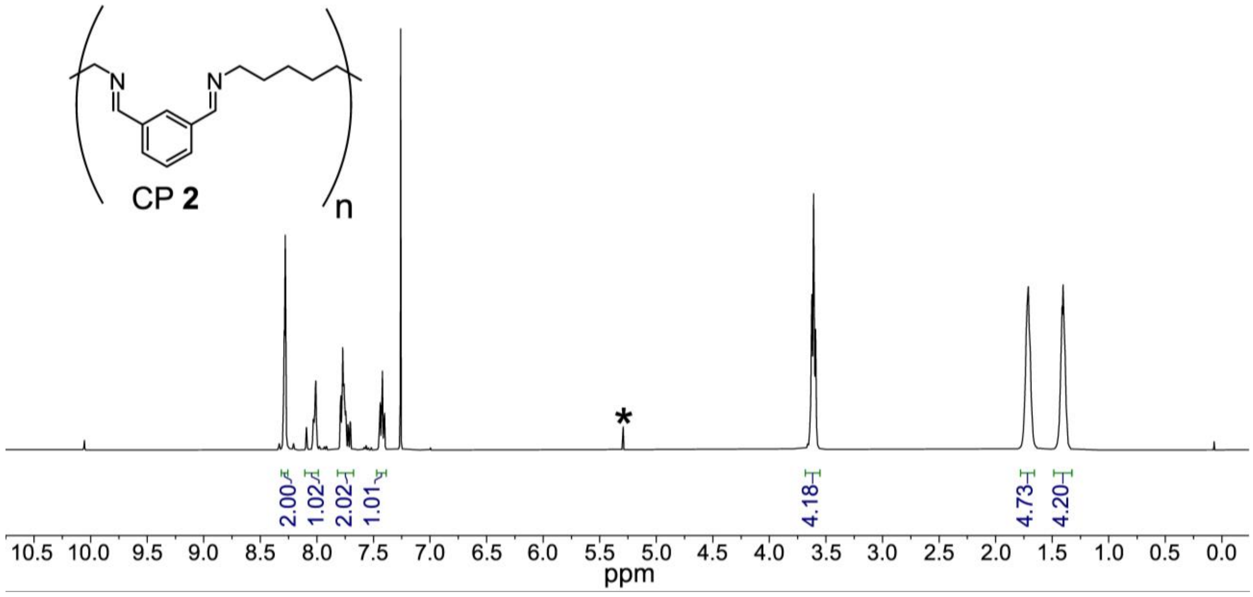

Figure S15. ^{1}H-NMR ($CDCl_3$) of CP **2** at 1 M monomer concentration, (residual dichloromethane marked with an asterisk).

SEC analysis of CP 2 synthesized at 1 M monomer concentration

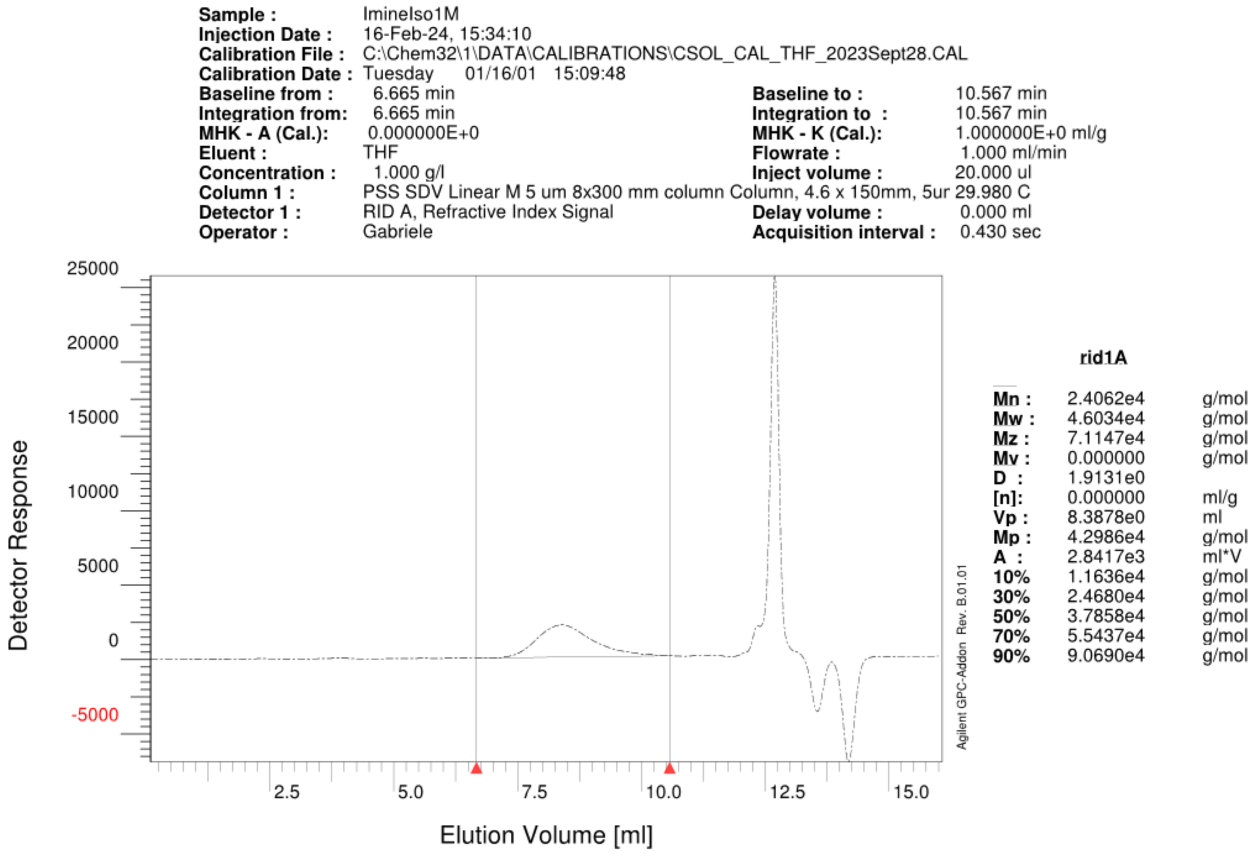

Figure S16. SEC analysis of CP **2** synthesized at 1 M.

For this poly(imine) the resulted $\overline{DP_n}$ is 112.

Transimination between CCM 1 and 4

In a NMR tube 78.7 mg (0.11 mmol) of CCM **1** were dissolved in 550 µL of a 400 mM solution ($CDCl_3$ / CD_3OD 3:1) of **4** (0.22 mmol). The mixture was heated at 50 °C for five days, and ^{1}H-NMR monitored up to equilibrium.

^{1}H-NMR spectrum of a transimination mixture of CCM 1 and 4

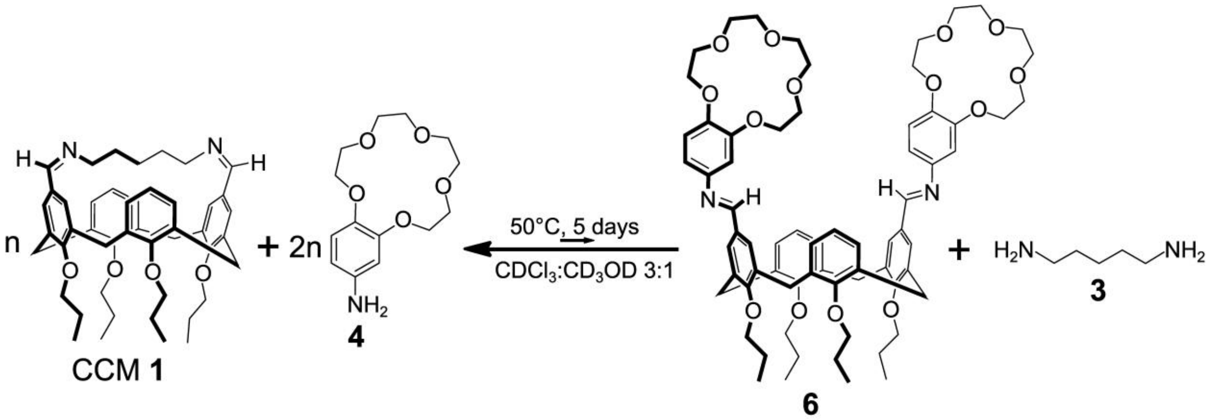

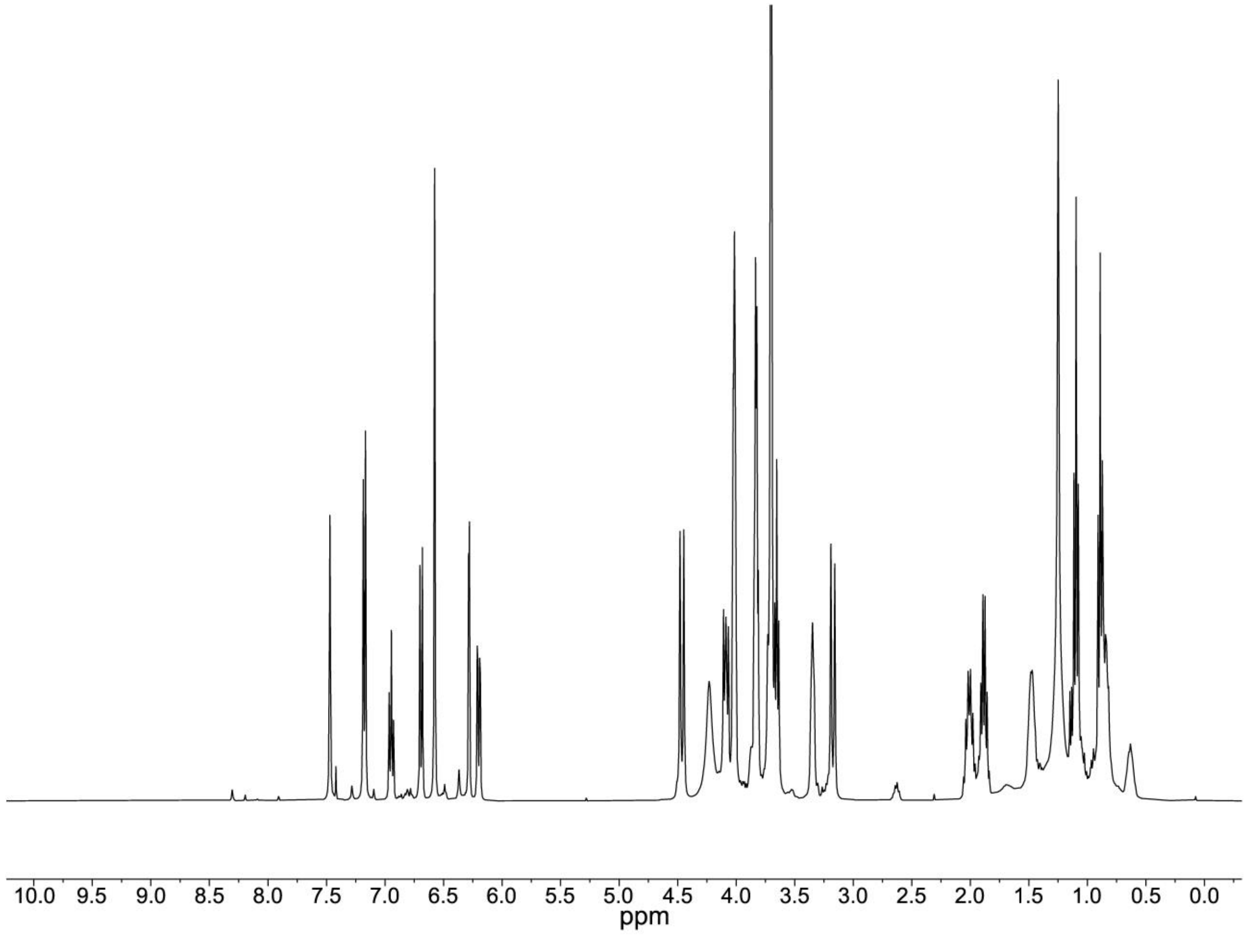

Figure S17. ^{1}H-NMR ($CDCl_3$ / CD_3OD 3:1) of a transimination mixture of CCM **1** and **4**, at equilibrium.

Transimination experiment between CP 2 and 4

In a NMR tube 11.8 mg (0.055 mmol) of CP **2** obtained at 500 mM concentration of monomers, were dissolved in 550 µL of a 200 mM solution of **4** (0.11 mmol) in $CDCl_3$ / CD_3OD 3:1. The mixture was heated at 50 °C for one day, and ^{1}H-NMR monitored up to equilibrium.

^{1}H-NMR spectrum of a transimination mixture of CP 2 and 4

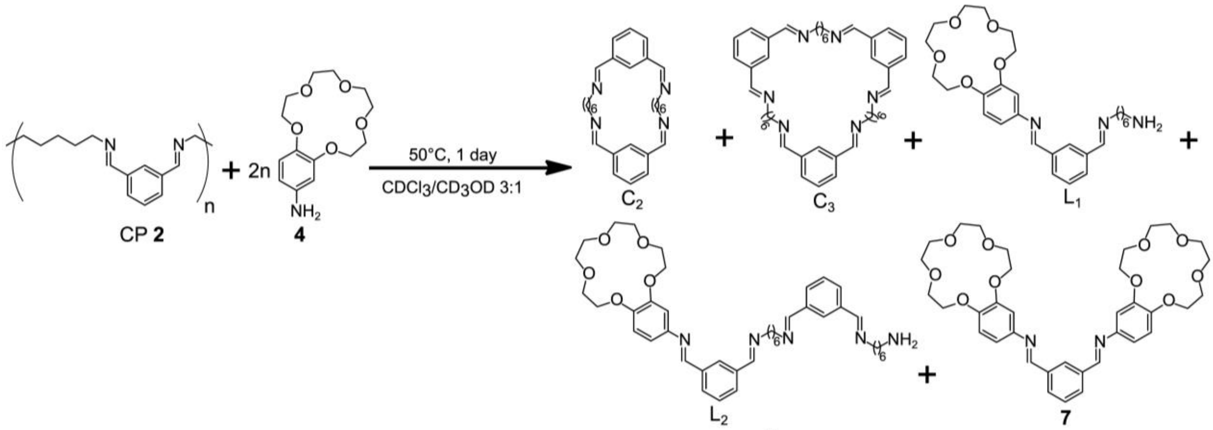

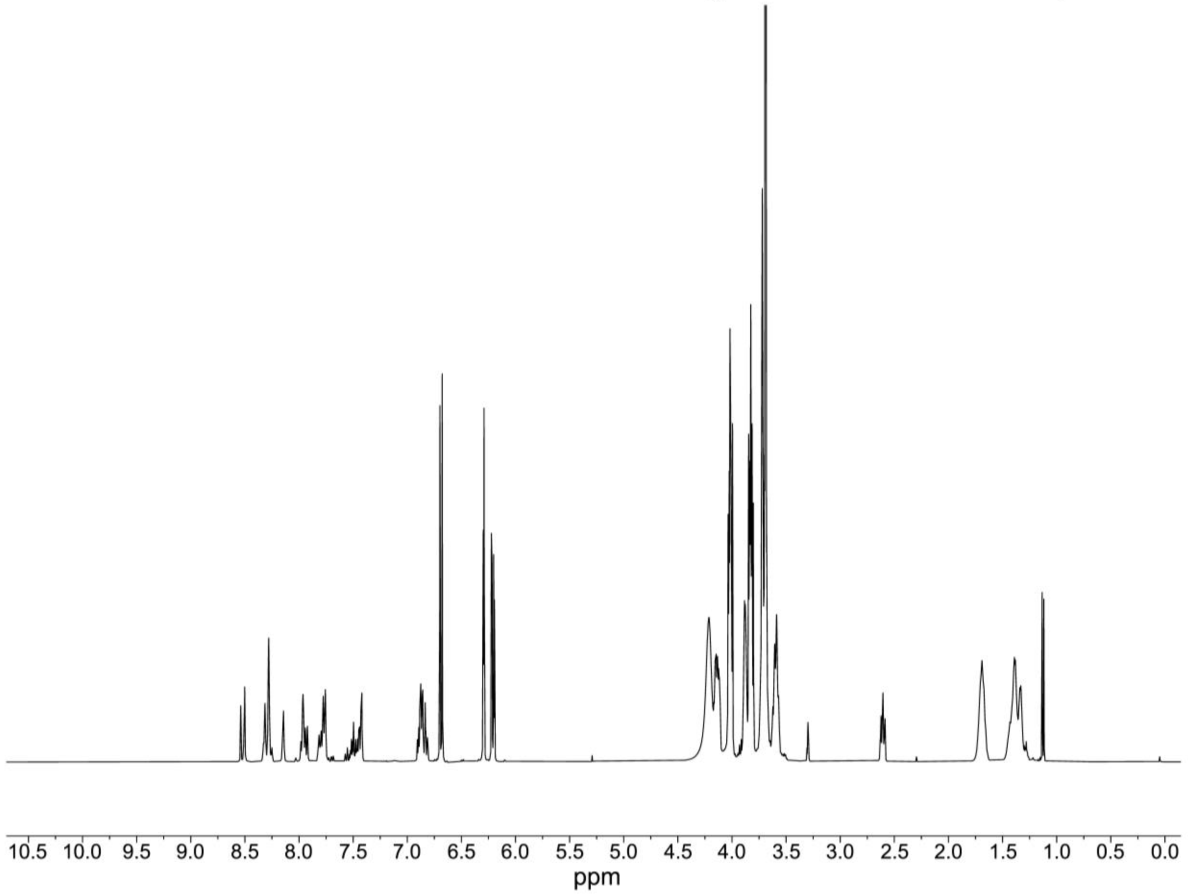

Figure S18. ^{1}H-NMR ($CDCl_3$ / CD_3OD 3:1) of a transimination mixture of CP **2** and **4**, at equilibrium.

ESI-MS analysis of a transimination mixture of CP 2 and 4

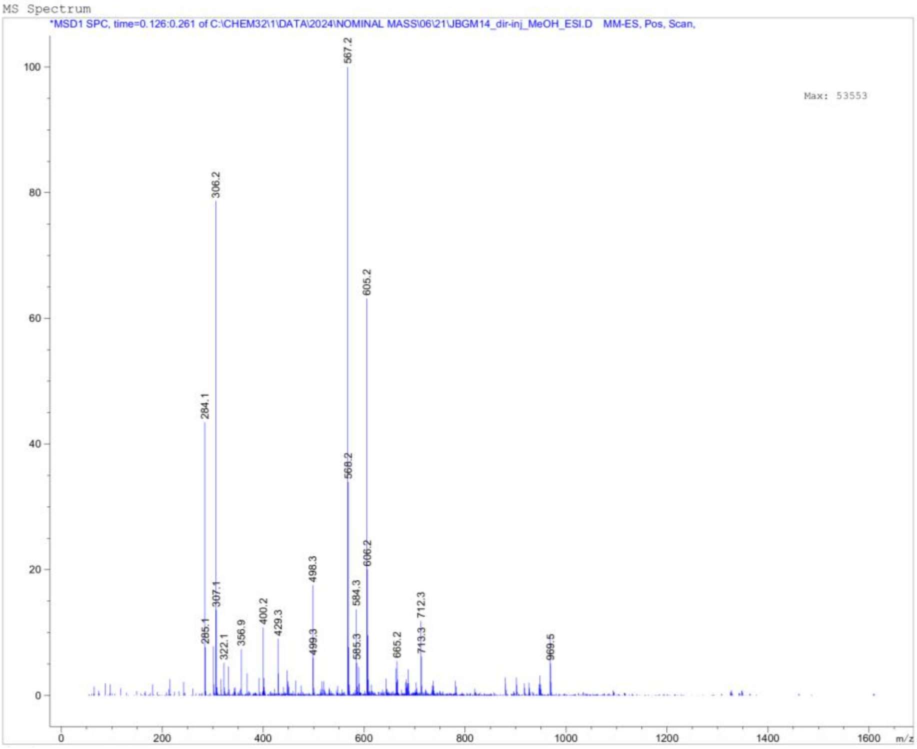

Figure S19. ESI-MS analysis of a mixture obtained from equilibration between CP **2** and **4**.

APCI-MS analysis of a transimination mixture of CP 2 and 4

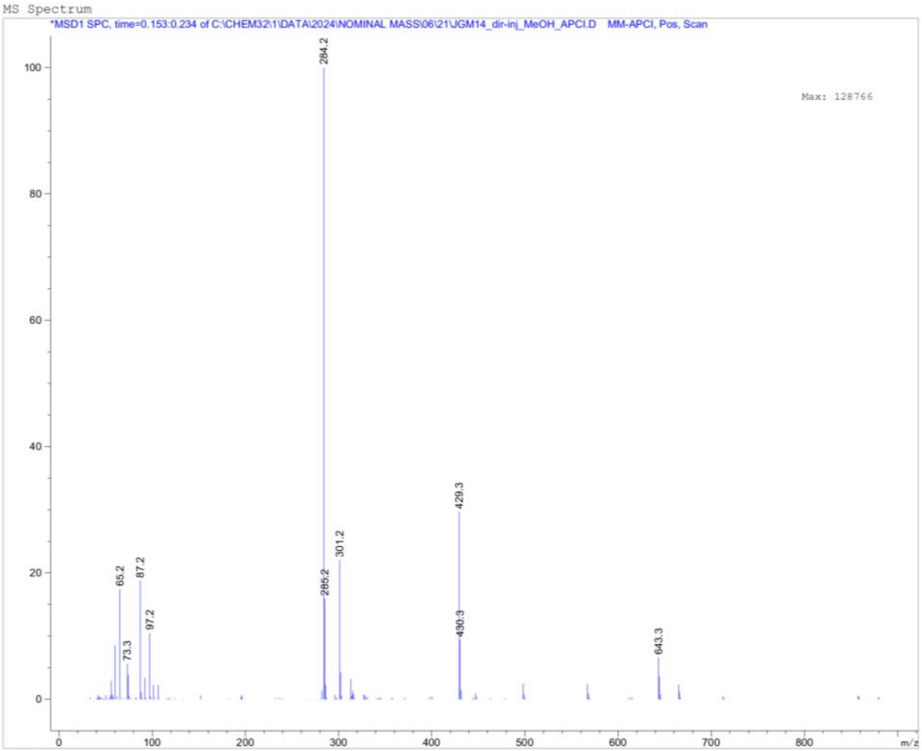

Figure S20. APCI-MS analysis of a mixture obtained from equilibration between CP **2** and **4**.

The charged adducts detected in the ESI-MS analysis of the transimination mixture between CP **2** and **4** at equilibrium are reported in Table 2.

Table S2. *m*/*z* values obtained from ESI and APCI-MS analysis of a mixture obtained from equilibration of CP **2** and **4**. a) These signals are also found in the ESI-MS spectrum of **4** (Figure **S21**).

Charged adducts detected	*m*/*z* value
[**4** + H]$^+$	284.1
[C_2 + H]$^+$	429.3
[L_1 + H]$^+$	498.3
[(**4**)$_2$ + H]$^+$	567.2[a]
[(**4**)$_2$ + K]$^+$	605.2[a]
[**7** + H]$^+$	665.2
[L_2 + H]$^+$	712.3
Impurity from **4**	968.5[a]

ESI-MS analysis of 4

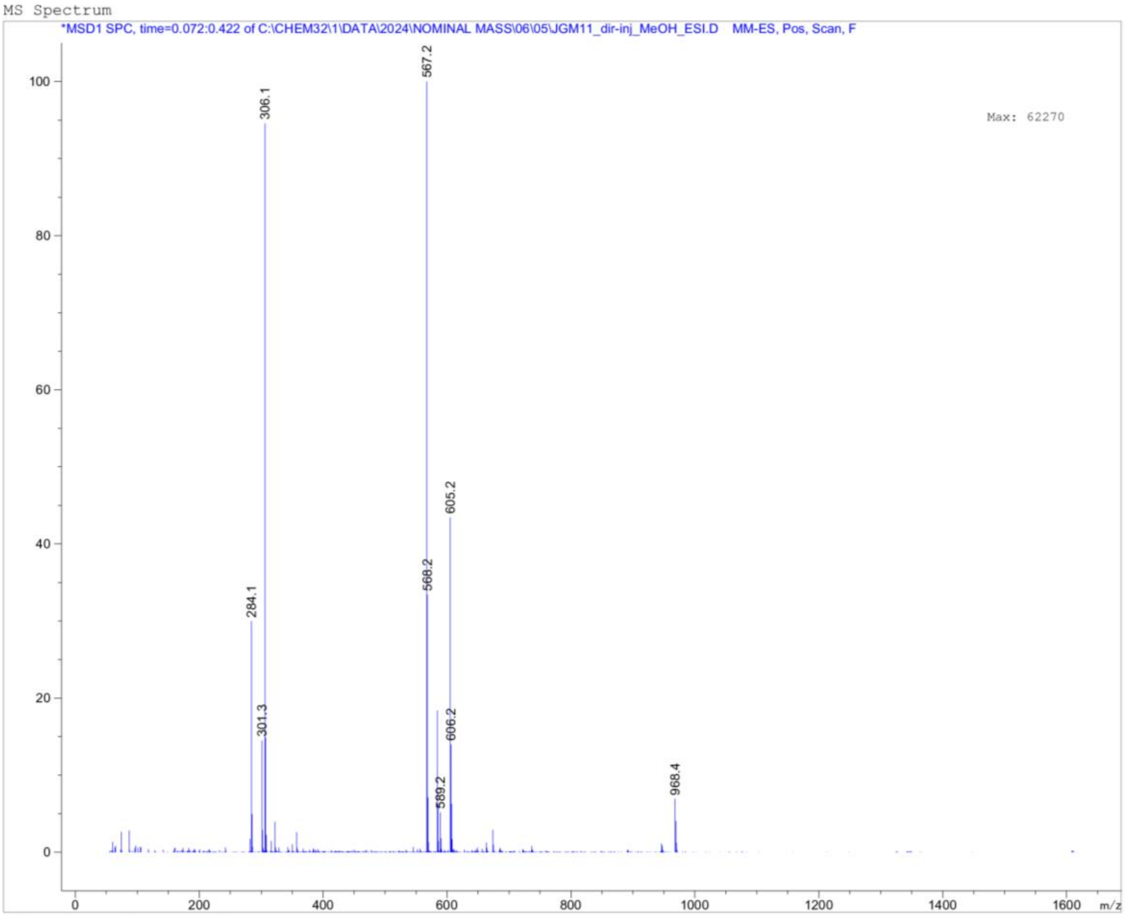

Figure S21. ESI-MS of **4**.

SEC analysis of a transimination mixture of CP 2 and 4

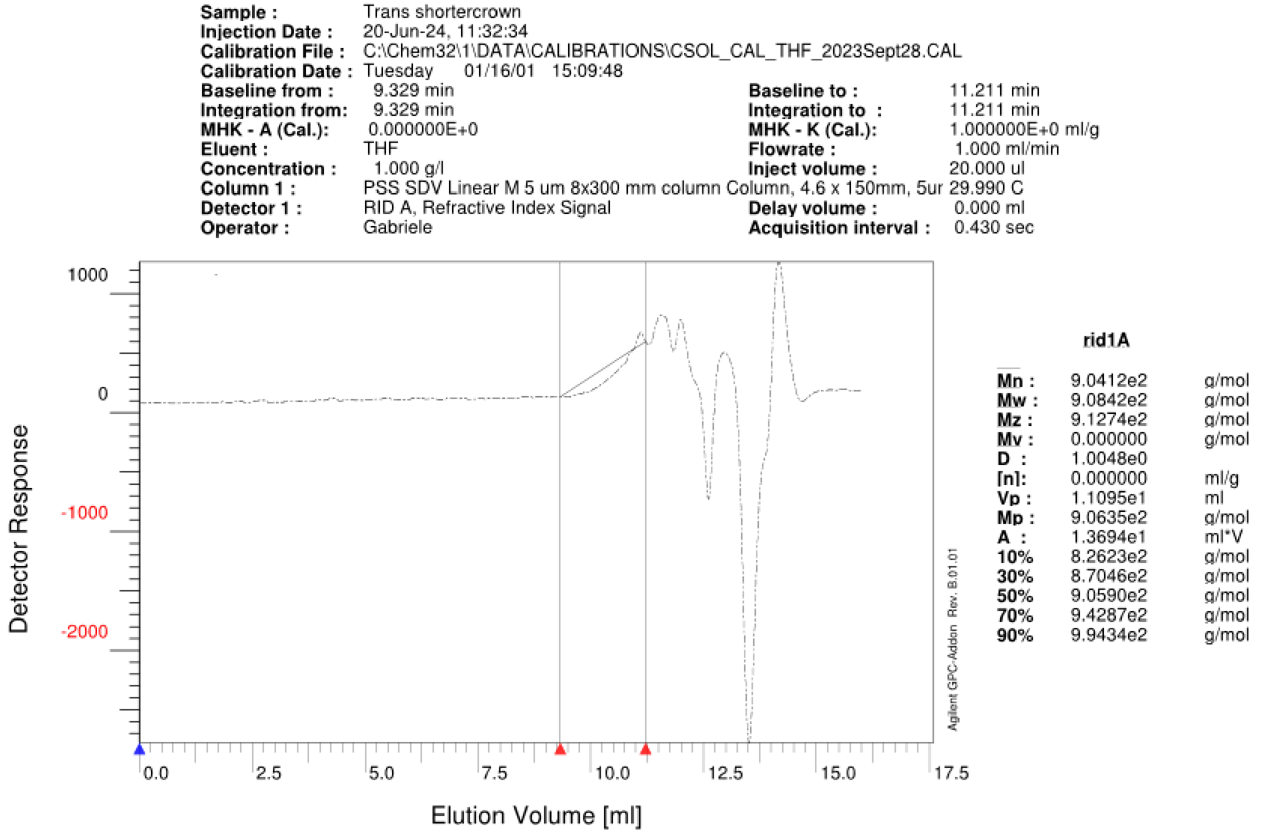

Figure S22. SEC analysis of a transimination mixture of CP **2** and **4**.

SEC analysis confirms the disappearance of the polymer peak of CP **2** (Figure S13), with the formation of lighter species that are eluted close to the mobile phase, and were observed by ESI and APCI-MS analysis (Figures S19 and S20).

Acid-induced transimination of 200 mM CCM 1 and 400 mM 4, in a 400 mM TFA solution

In a 4 mL vial 85.8 mg (0.120 mmol) of CCM **1** were dissolved in 600 μL of a 400 mM solution of **4** (0.240 mmol) in $CDCl_3$ / CD_3OD 3:1. Then, 18.4 μL (0.240 mmol) of TFA were added, and the mixture was studied by 1D and 2D-DOSY ^{1}H-NMR.

^{1}H-NMR spectrum of a 200 mM CCM 1, 400 mM 4, and 400 mM TFA solution

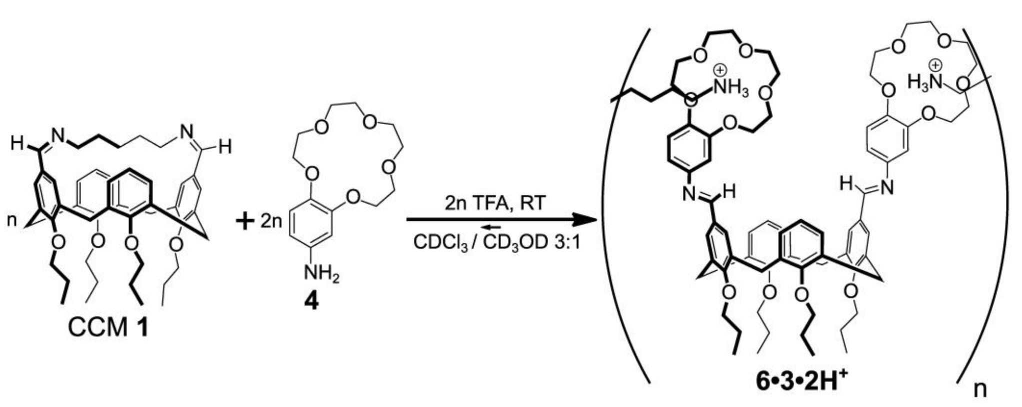

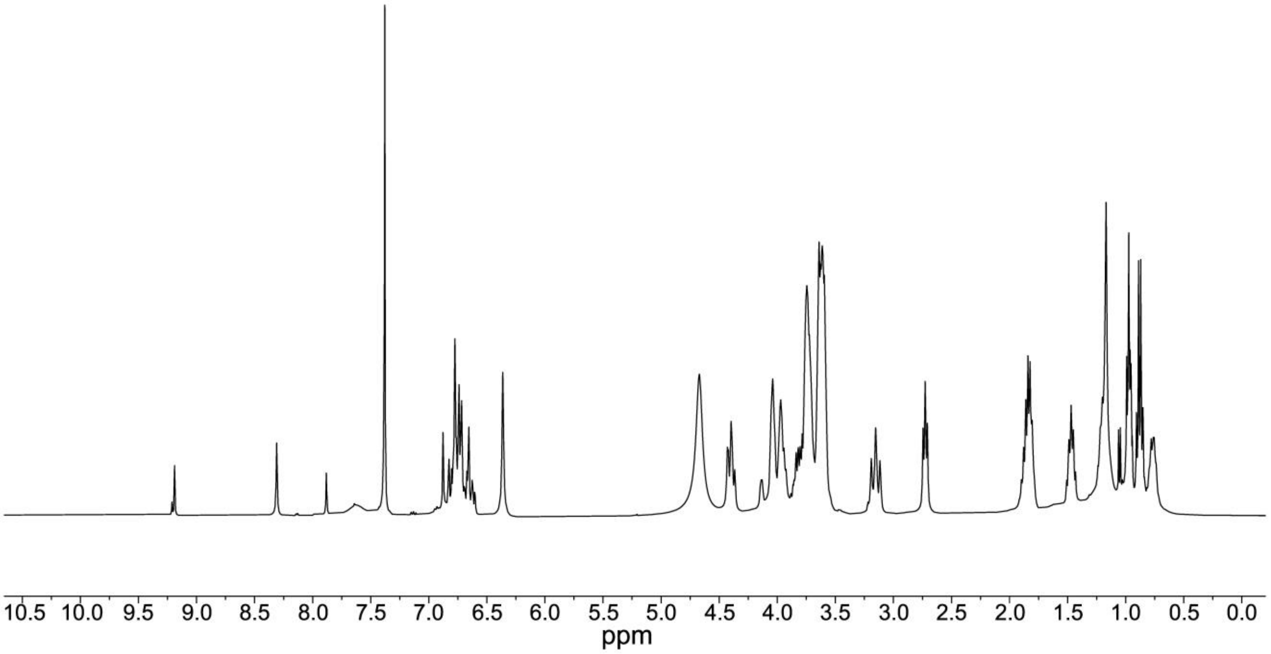

Figure S23. ^{1}H-NMR ($CDCl_3$ / CD_3OD 3:1) of a 200 mM CCM **1**, 400 mM **4**, and 400 mM TFA solution.

DOSY spectrum (Bayesian mode) of a 200 mM CCM 1, 400 mM 4, and 400 mM TFA solution

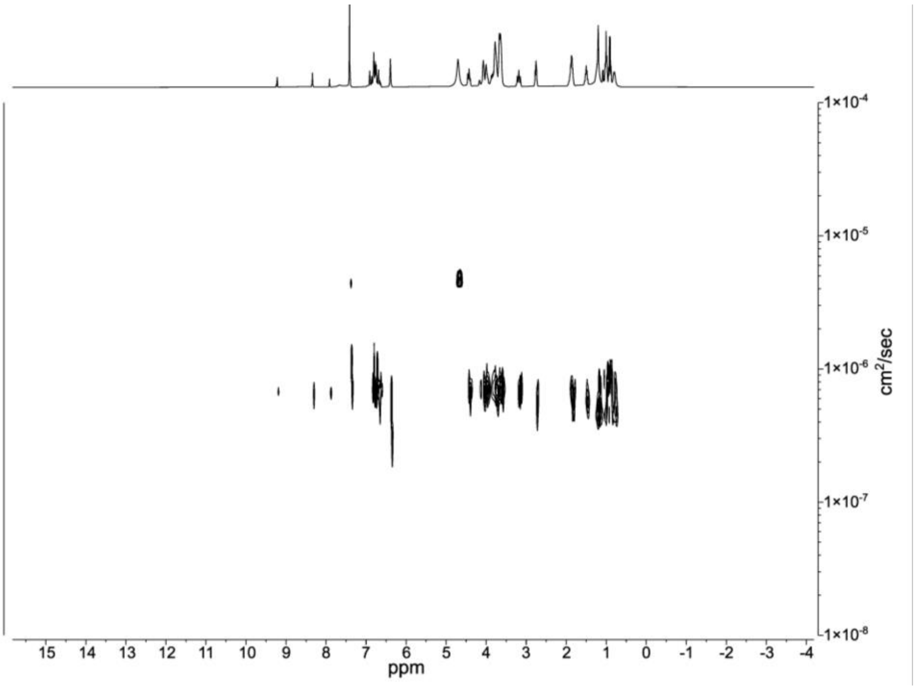

Figure S24. DOSY spectrum (Bayesian mode), recorded in $CDCl_3$ / CD_3OD 3:1 at RT, of a 200 mM CCM **1**, 400 mM **4**, and 400 mM TFA solution.

DOSY spectrum (Peak fit mode) of a 200 mM CCM 1, 400 mM 4, and 400 mM TFA solution

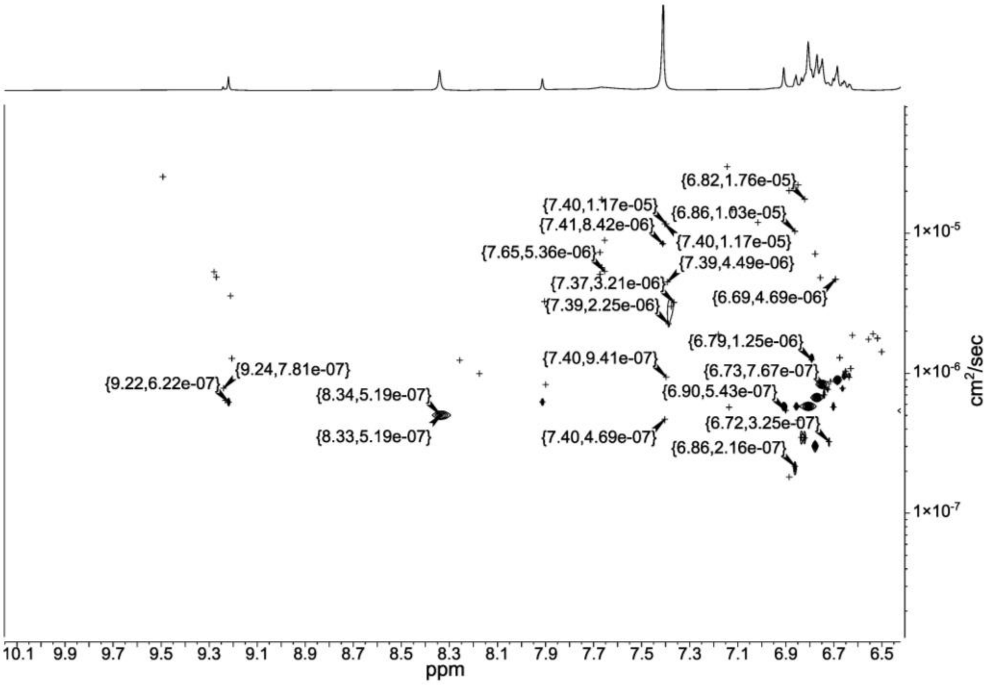

Figure S25. DOSY spectrum (Peak fit mode) of a 200 mM CCM **1**, 400 mM **4**, and 400 mM TFA solution, recorded in $CDCl_3$ / CD_3OD 3:1 at RT.

From Peak fit mode the diffusion coefficient value $5.19 \cdot 10^{-7}$ cm^2/s was obtained for **6**.

Acid-induced transimination of 175 mM CCM 1 and 350 mM 4, in a 350 mM TFA solution

In a 4 mL vial 75.2 mg (0.105 mmol) of CCM **1** were dissolved in 600 µL of a 350 mM solution of **4** (0.210 mmol) in $CDCl_3$ / CD_3OD 3:1. Then, 16.1 µL (0.21 mmol) of TFA were added, and the mixture was studied by 1D and 2D-DOSY ^{1}H-NMR.

^{1}H-NMR spectrum of a 175 mM CCM 1, 350 mM 4, and 350 mM TFA solution

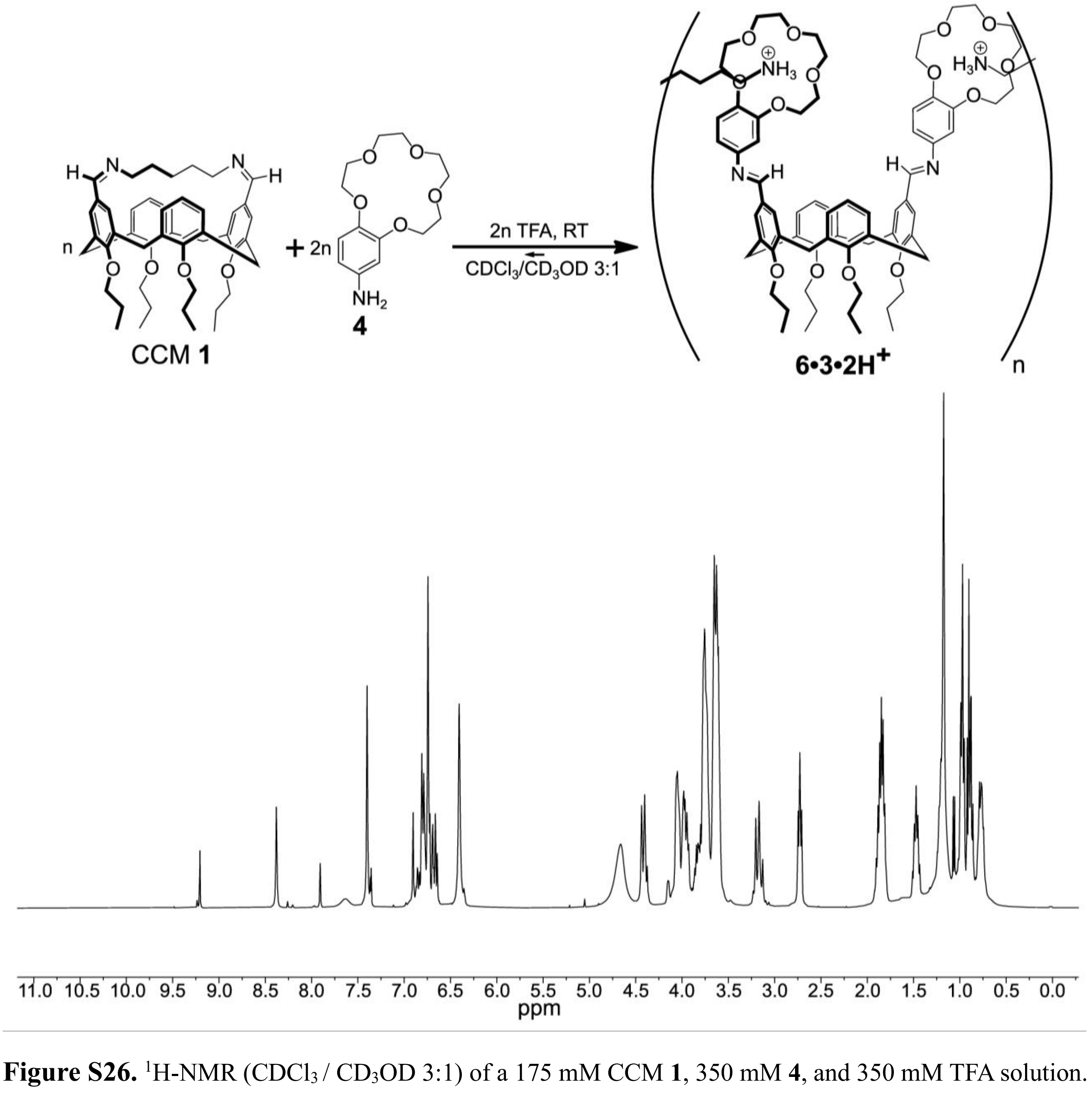

Figure S26. ^{1}H-NMR ($CDCl_3$ / CD_3OD 3:1) of a 175 mM CCM **1**, 350 mM **4**, and 350 mM TFA solution.

DOSY spectrum (Bayesian mode) of a 175 mM CCM 1, 350 mM 4, and 350 mM TFA solution

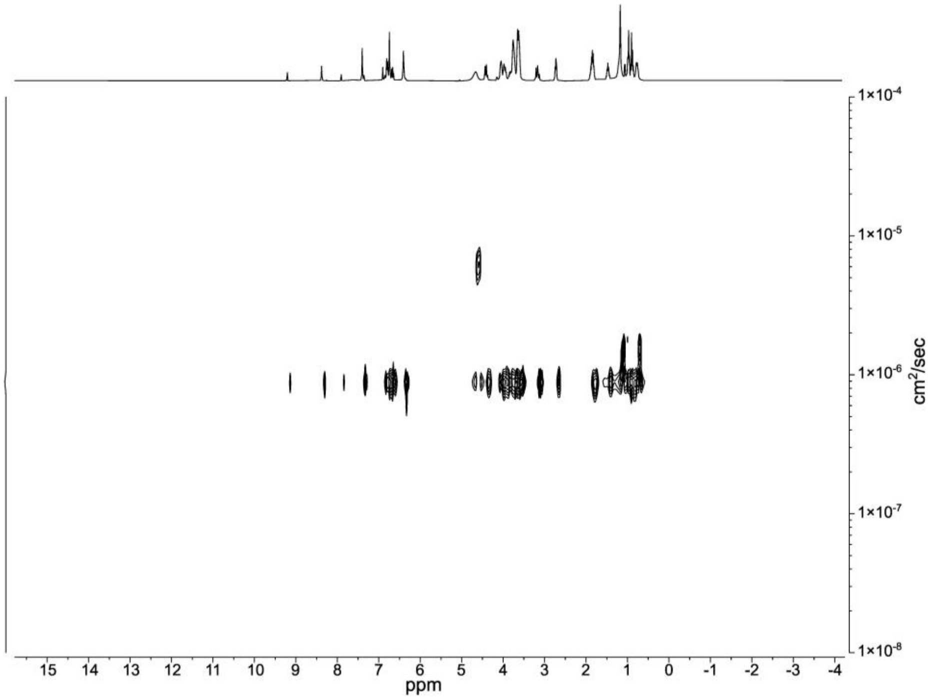

Figure S27. DOSY spectrum (Bayesian mode), recorded in $CDCl_3$ / CD_3OD 3:1 at RT, of a 175 mM CCM **1**, 350 mM **4**, and 350 mM TFA solution.

DOSY spectrum (Peak fit mode) of a 175 mM CCM 1, 350 mM 4, and 350 mM TFA solution

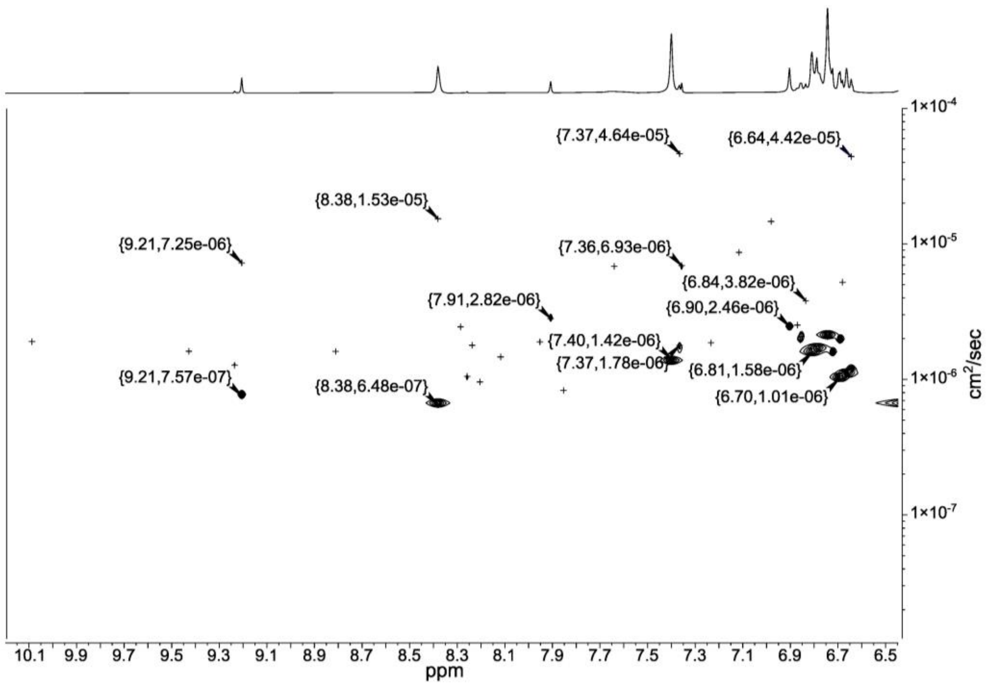

Figure S28. DOSY spectrum (Peak fit mode) of a 175 mM CCM **1**, 350 mM **4**, and 350 mM TFA solution, recorded in $CDCl_3$ / CD_3OD 3:1 at RT.

From Peak fit mode the diffusion coefficient value $6.48 \cdot 10^{-7}$ cm^2/s was obtained for **6**.

Acid-induced transimination of 150 mM CCM 1 and 300 mM 4, in a 300 mM TFA solution

In a 4 mL vial 64.3 mg (0.090 mmol) of CCM **1** were dissolved in 600 µL of a 300 mM solution of **4** (0.180 mmol) in $CDCl_3$ / CD_3OD 3:1. Then, 13.8 µL (0.180 mmol) of TFA were added, and the mixture was studied by 1D and 2D-DOSY ^{1}H-NMR.

^{1}H-NMR spectrum of a 150 mM CCM 1, 300 mM 4, and 300 mM TFA solution

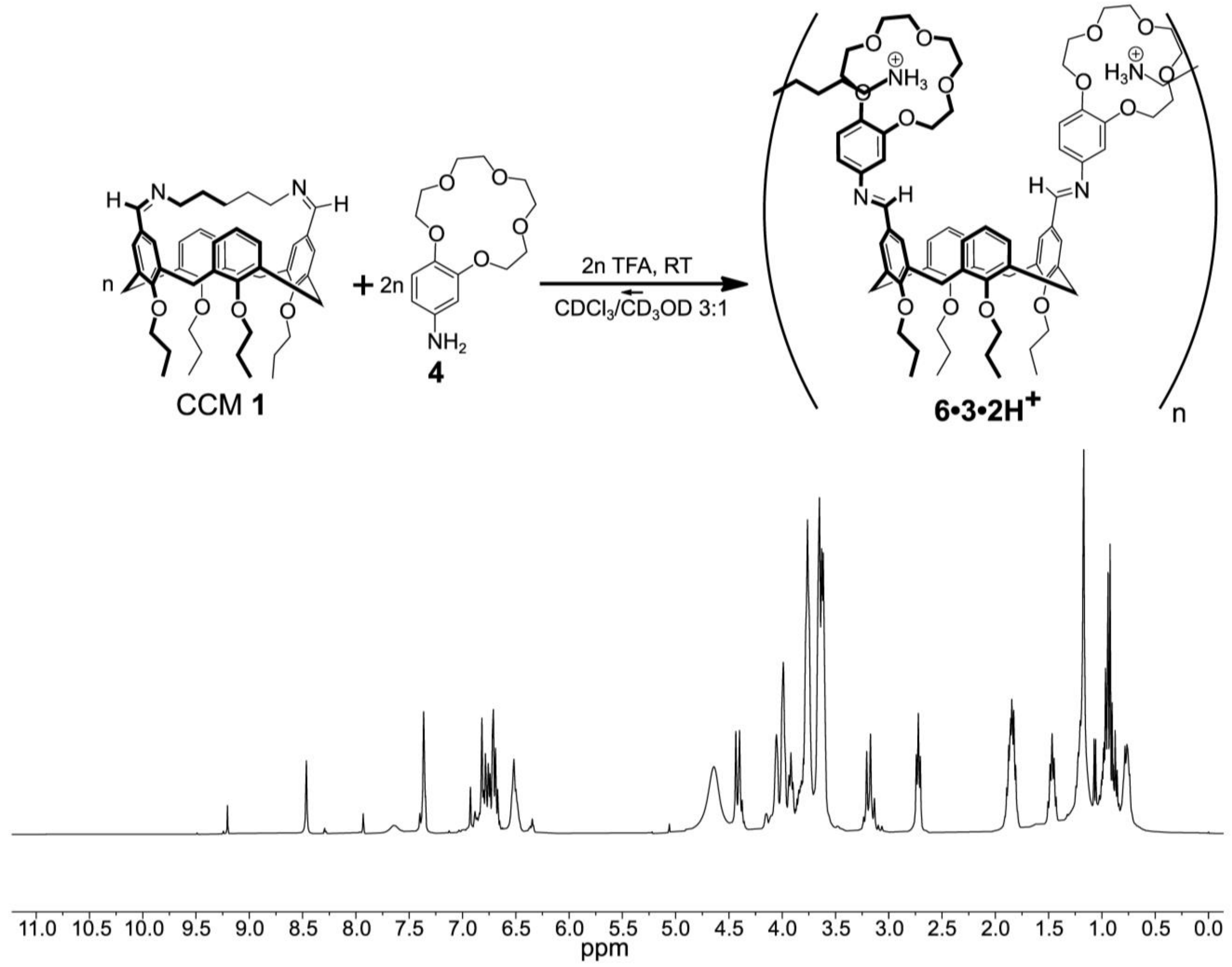

Figure S29. ^{1}H-NMR ($CDCl_3$ / CD_3OD 3:1) of a 150 mM CCM **1**, 300 mM **4**, and 300 mM TFA solution.

DOSY spectrum (Bayesian mode) of a 150 mM CCM 1, 300 mM 4, and 300 mM TFA solution

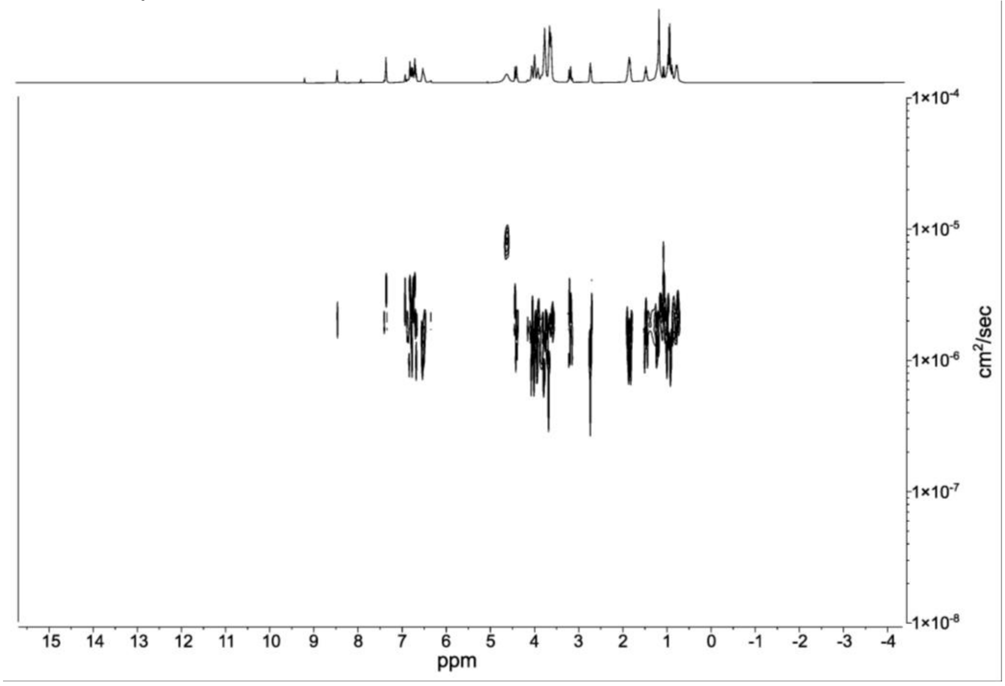

Figure S30. DOSY spectrum (Bayesian mode), recorded in $CDCl_3$ / CD_3OD 3:1 at RT, of a 150 mM CCM **1**, 300 mM **4**, and 300 mM TFA solution

DOSY spectrum (Peak fit mode) of a 150 mM CCM 1, 300 mM 4, and 300 mM TFA solution

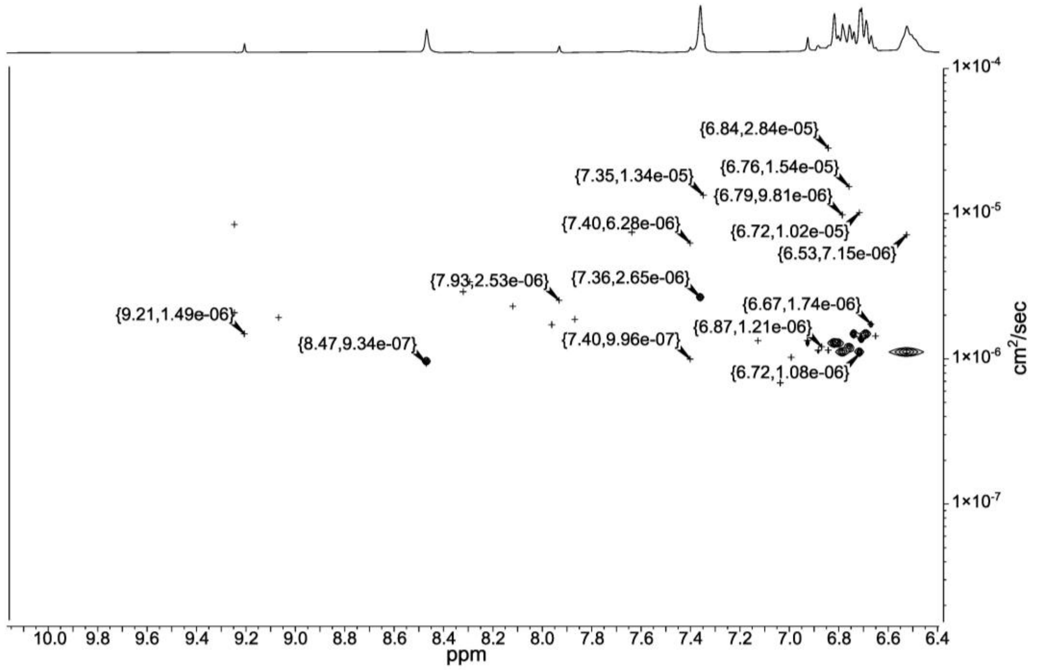

Figure S31. DOSY spectrum (Peak fit mode) of a 150 mM CCM **1**, 300 mM **4**, and 300 mM TFA solution, recorded in $CDCl_3$ / CD_3OD 3:1 at RT.

From Peak fit mode the diffusion coefficient value $9.34 \cdot 10^{-7}$ cm^2/s was obtained for **6**.

Acid-induced transimination of 100 mM CCM 1 and 200 mM 4, in a 200 mM TFA solution

In a 4 mL vial 42.9 mg (0.060 mmol) of CCM **1** were dissolved in 600 µL of a 200 mM solution of **4** (0.120 mmol) in $CDCl_3$ / CD_3OD 3:1. Then, 9.2 µL (0.12 mmol) of TFA were added, and the mixture was studied by 1D and 2D-DOSY ^{1}H-NMR.

^{1}H-NMR spectrum of a 100 mM CCM 1, 200 mM 4, and 200 mM TFA solution

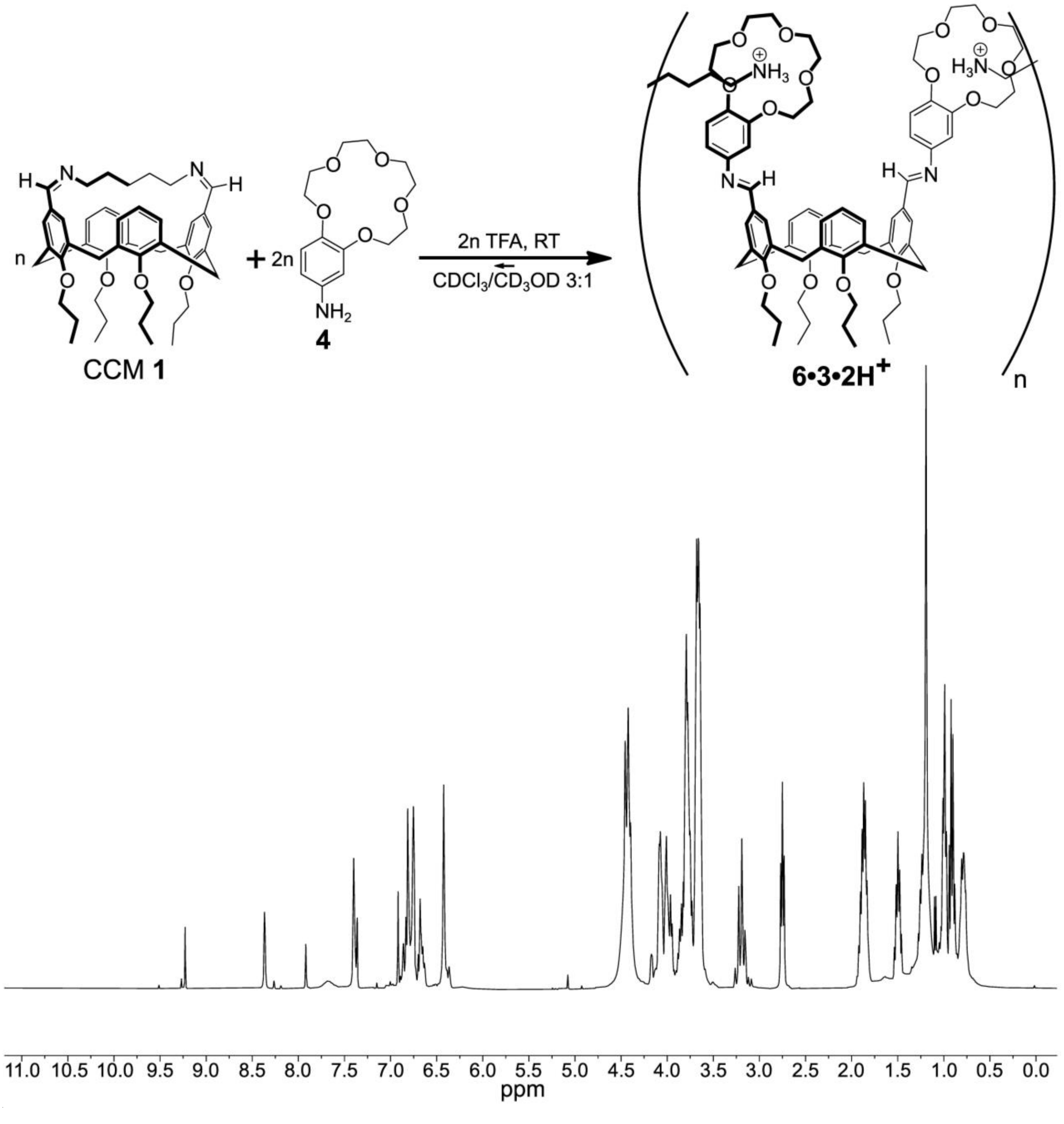

Figure S32. ^{1}H-NMR ($CDCl_3$ / CD_3OD 3:1) of a 100 mM CCM **1**, 200 mM **4**, and 200 mM TFA solution.

DOSY spectrum (Bayesian mode) of a 100 mM CCM 1, 200 mM 4, and 200 mM TFA solution

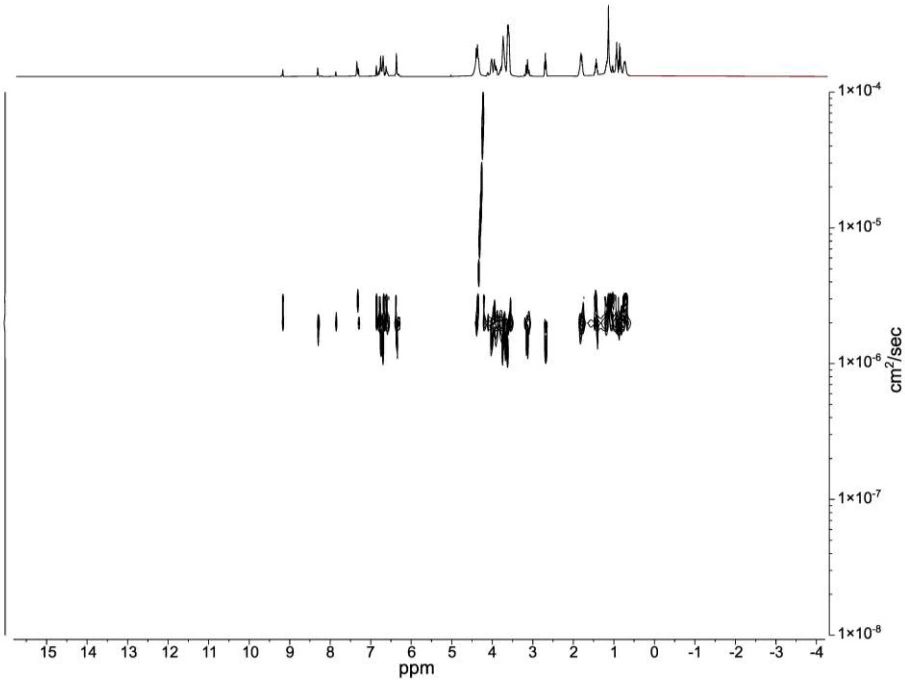

Figure S33. DOSY spectrum (Bayesian mode), recorded in $CDCl_3$ / CD_3OD 3:1 at RT, of a 100 mM CCM **1**, 200 mM **4**, and 200 mM TFA solution.

DOSY spectrum (Peak fit mode) of a 100 mM CCM 1, 200 mM 4, and 200 mM TFA solution

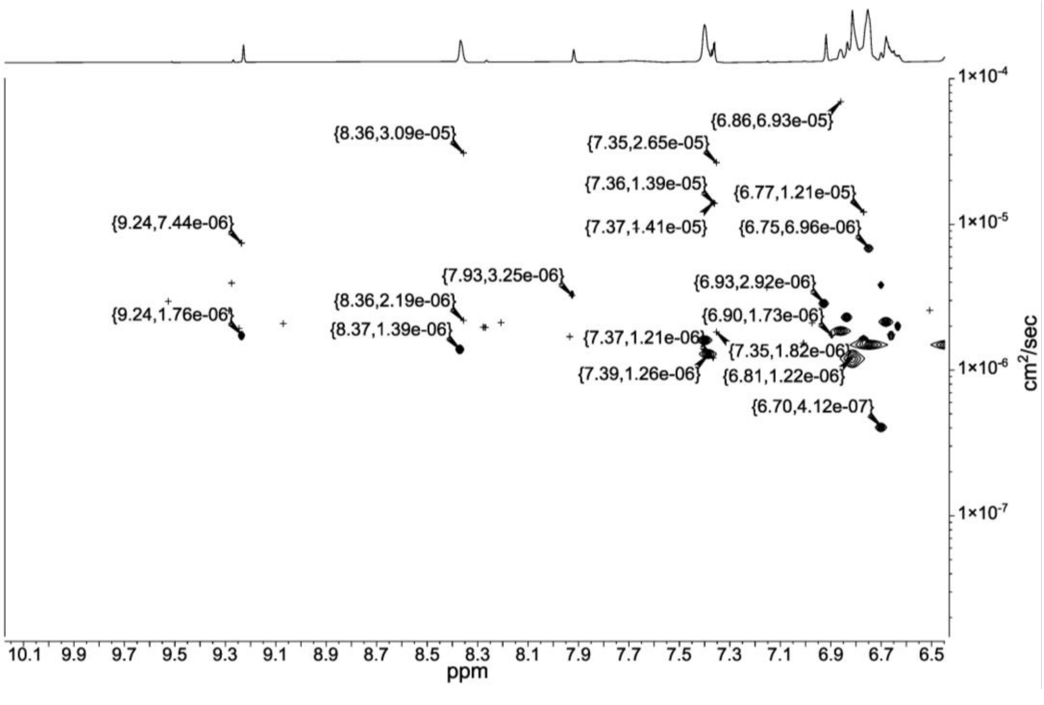

Figure S34. DOSY spectrum (Peak fit mode) of a 100 mM CCM **1**, 200 mM **4**, and 200 mM TFA solution, recorded in $CDCl_3$ / CD_3OD 3:1 at RT.

From Peak fit mode the diffusion coefficient value $1.39 \cdot 10^{-6}$ cm^2/s was obtained for **6**.

Acid-induced transimination of 50 mM CCM 1 and 100 mM 4, in a 100 mM TFA solution

In a 4 mL vial 21.4 mg (0.030 mmol) of CCM **1** were dissolved in 600 μL of a 100 mM solution of **4** (0.060 mmol) in $CDCl_3$ / CD_3OD 3:1. Then, 4.6 μL (0.060 mmol) of TFA were added, and the mixture was studied by 1D and 2D-DOSY ^{1}H-NMR.

^{1}H-NMR spectrum of a 50 mM CCM 1, 100 mM 4, and 100 mM TFA solution

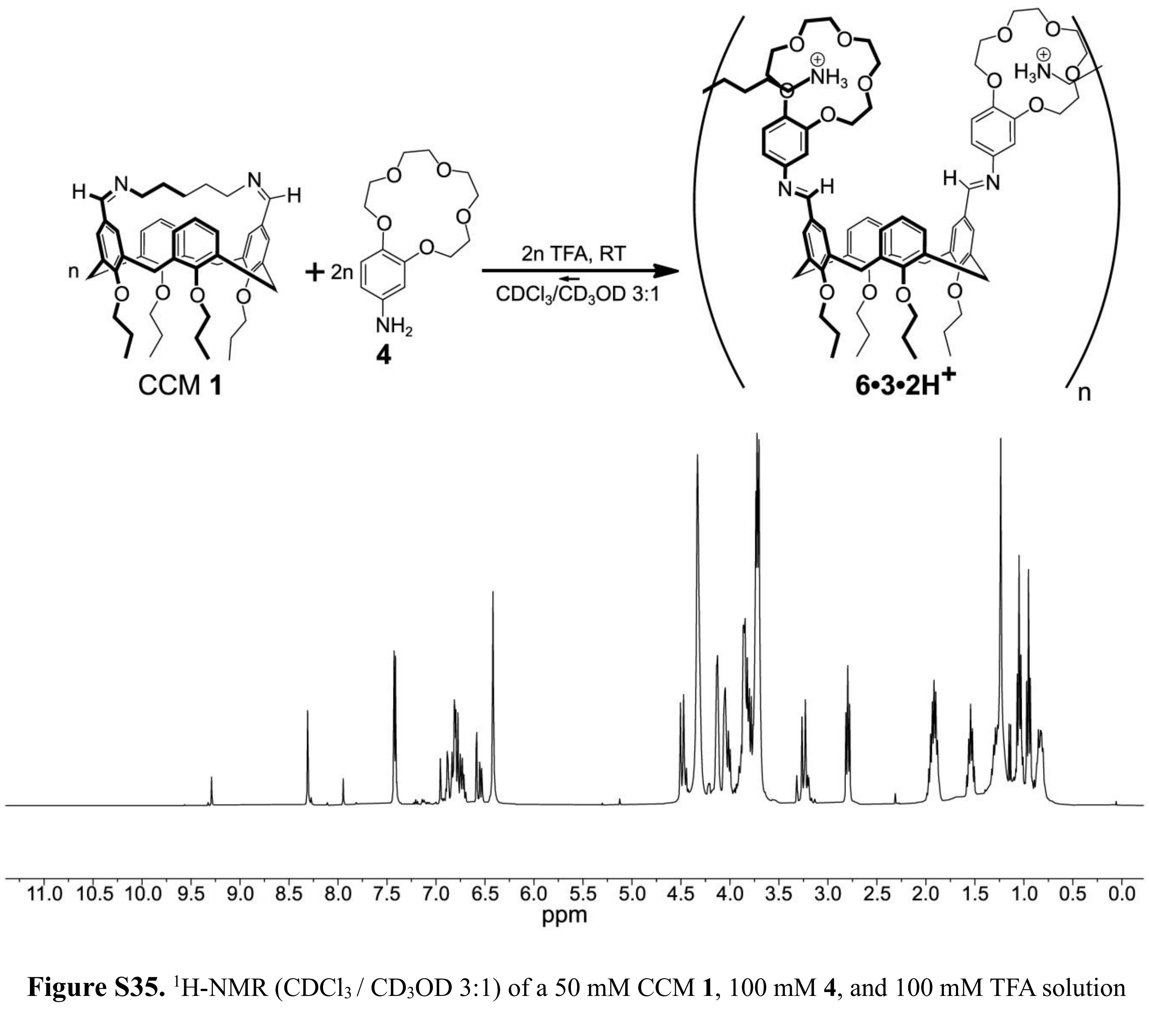

Figure S35. ^{1}H-NMR ($CDCl_3$ / CD_3OD 3:1) of a 50 mM CCM **1**, 100 mM **4**, and 100 mM TFA solution

DOSY spectrum (Bayesian mode) of a 50 mM CCM 1, 100 mM 4, and 100 mM TFA solution

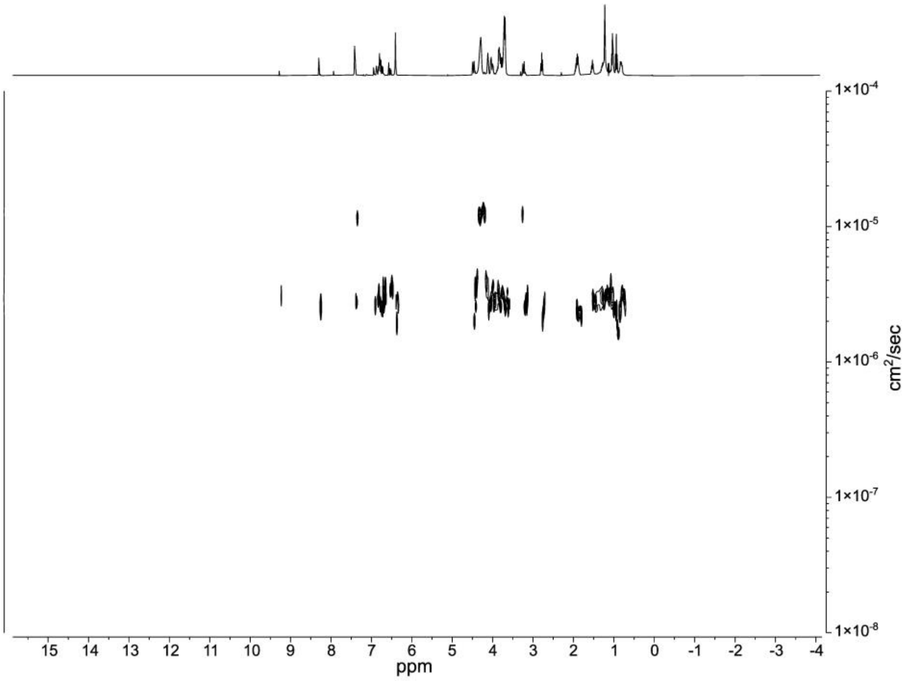

Figure S36. DOSY spectrum (Bayesian mode), recorded in $CDCl_3$ / CD_3OD 3:1 at RT, of a 50 mM CCM **1**, 100 mM **4**, and 100 mM TFA solution.

DOSY spectrum (Peak fit mode) of a 50 mM CCM 1, 100 mM 4, and 100 mM TFA solution

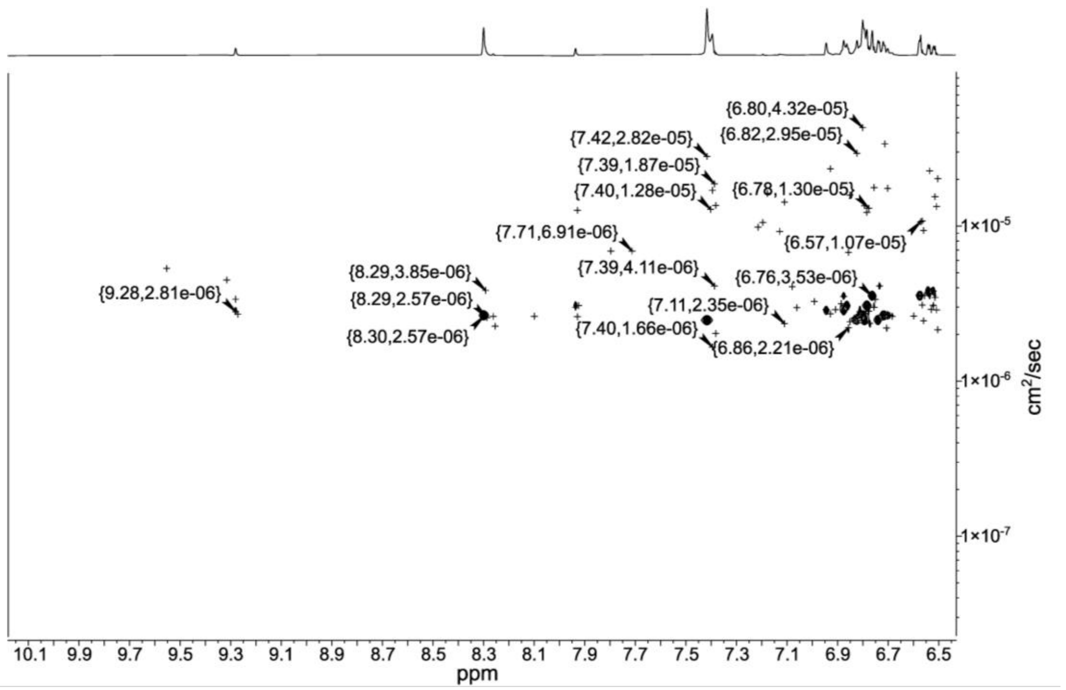

Figure S37. DOSY spectrum (Peak fit mode) of a 50 mM CCM **1**, 100 mM **4**, and 100 mM TFA solution, recorded in $CDCl_3$ / CD_3OD 3:1 at RT.

From Peak fit mode the diffusion coefficient value $2.57 \cdot 10^{-6}$ cm^2/s was obtained for **6**.

Synthesis of monomer 6

In a 10 mL round bottom flask 29.2 mg (0.0450 mmol) of 5,17-diformyl-25,26,27,28-tetrapropoxycalix[4]arene were dissolved in 4 mL of toluene. Then, 51.0 mg (0.180 mmol) of **4** were added to the solution, which was refluxed and stirred overnight. A two-fold excess of **4** was added in order to shift the equilibrium toward **6**. Once cooled down, solvent was removed under vacuum and the resulting solid mixture characterized by 1D and 2D-DOSY ^{1}H-NMR. ^{1}H-NMR analysis revealed a yield of 85% for **6** (Figure S38), with a 15% monoaldehyde by-product observed. For 2D-DOSY NMR, the solid was dissolved in 900 µL of a $CDCl_3$ / CD_3OD 3:1 solution, to have 50 mM concentration of **6**. ^{1}H NMR (400 MHz, CD_3OD) δ: 8.30 (s, 2H), 7.46 (s, 4H), 6.82 – 6.77 (m, 4H), 6.71 (d, J = 2.5 Hz, 1H), 6.69 (d, J = 2.2 Hz, 1H), 6.34 (s, 6H), 4.47 (d, J = 13.3 Hz, 4H), 3.24 (d, J = 13.5 Hz, 4H), 1.92 (dq, J = 17.2, 7.5 Hz, 8H), 1.06 (t, J = 7.5 Hz, 6H), 0.93 (t, J = 7.5 Hz, 6H).

^{1}H-NMR spectrum of monomer 6

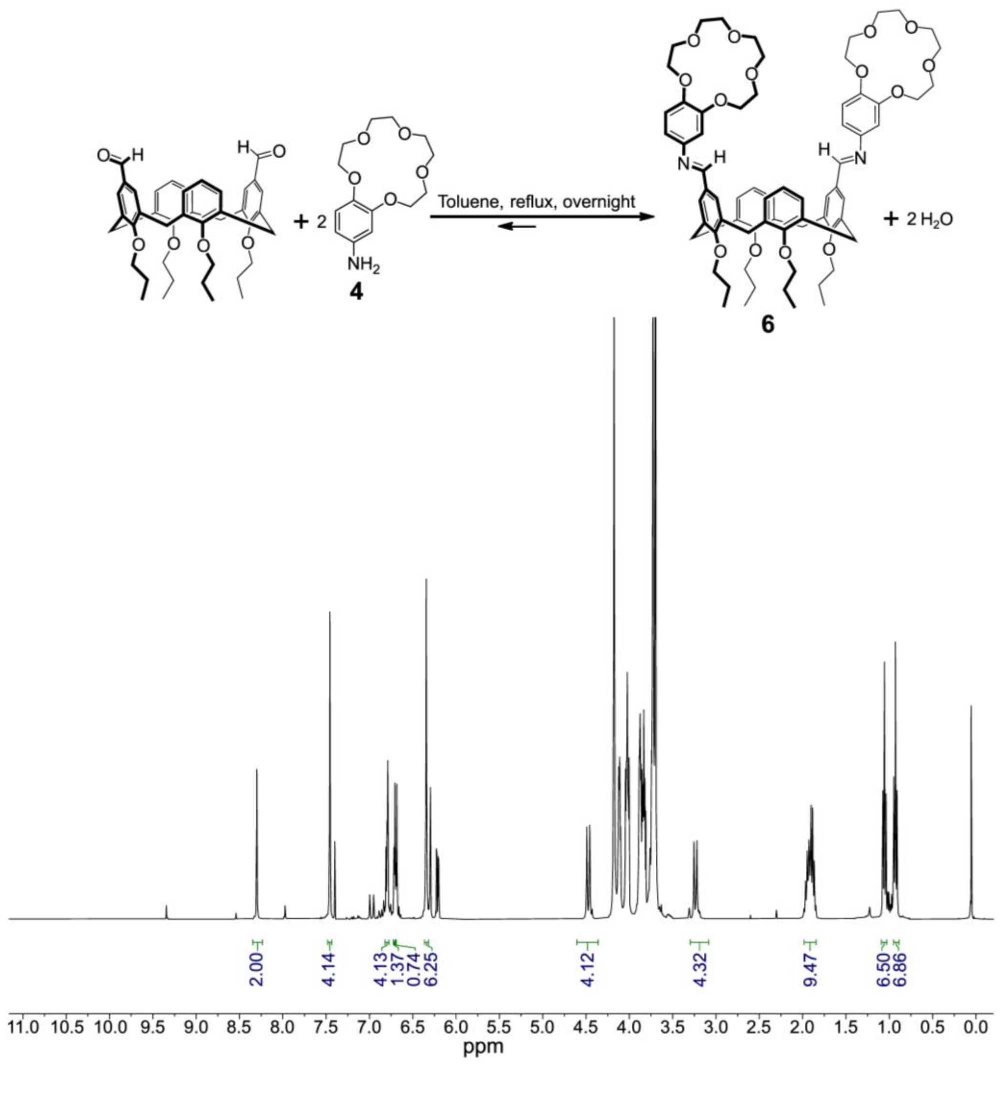

Figure S38. ^{1}H-NMR ($CDCl_3$ / CD_3OD 3:1) of monomer **6**.

DOSY spectrum (Bayesian mode) of 50 mM monomer 6

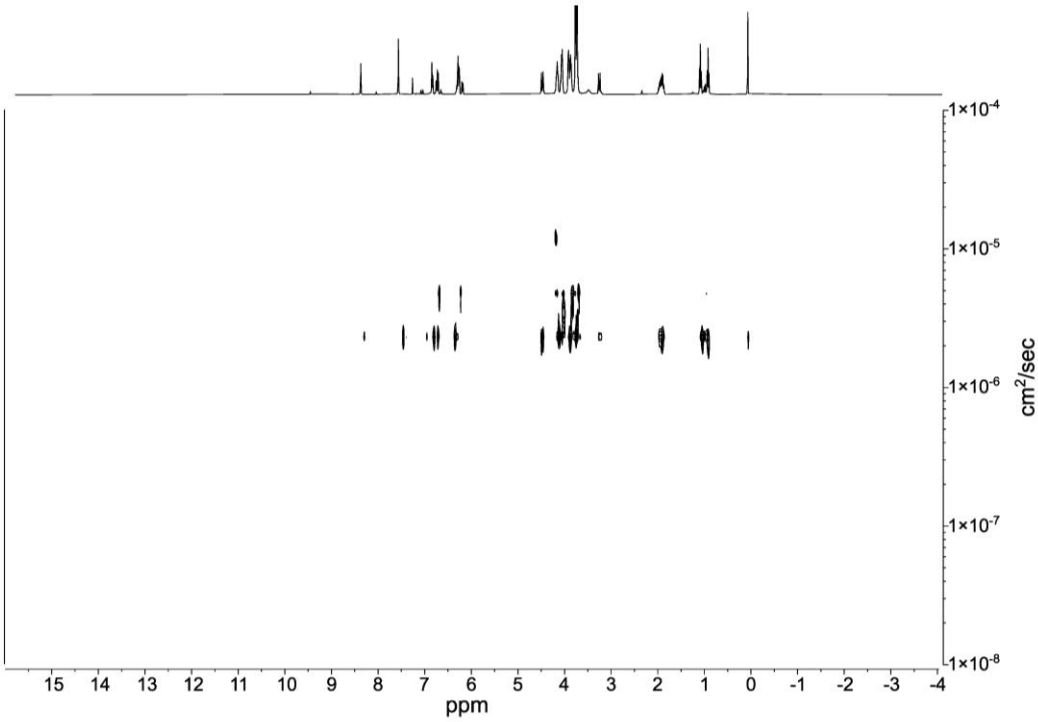

Figure S39. DOSY spectrum (Bayesian mode) of 50 mM monomer **6**, recorded in $CDCl_3$ / CD_3OD 3:1 at RT.

DOSY spectrum (Peak fit mode) of 50 mM monomer 6

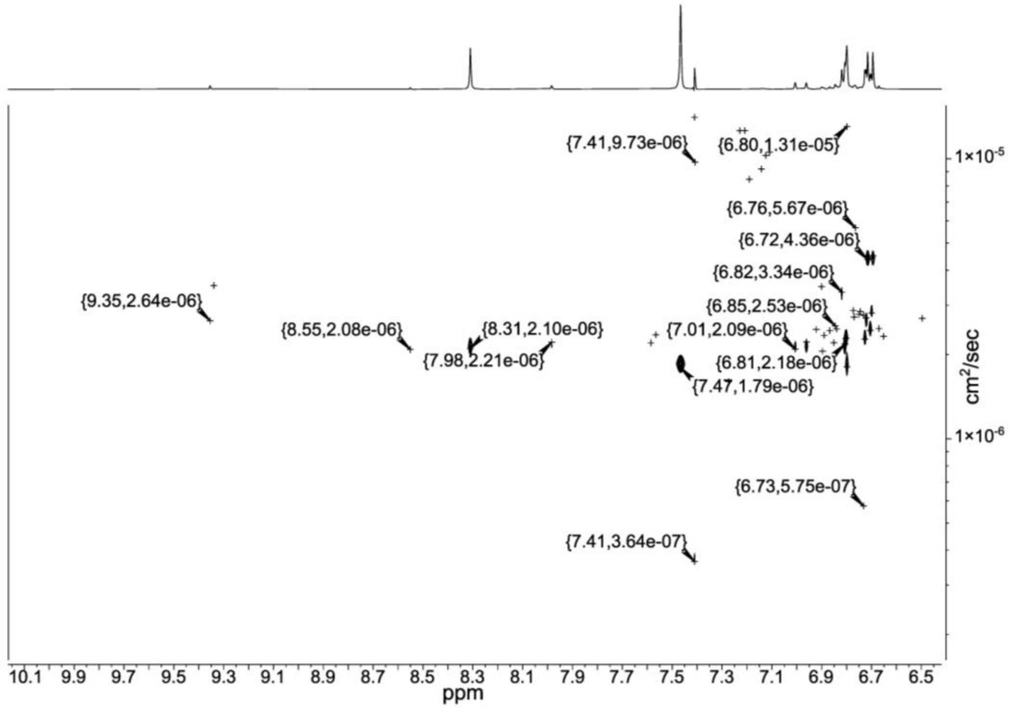

Figure S40. DOSY spectrum (Peak fit) of 50 mM monomer **6**, recorded in $CDCl_3$ / CD_3OD 3:1 at RT.

From Peak fit mode the diffusion coefficient value $2.10 \cdot 10^{-6}$ cm^2/s was obtained for **6**.

Degree of polymerization (DP) of SP-α

The average DP of supramolecular polymers was calculated from the diffusion coefficients obtained by DOSY experiments, using Equation S2,[3] derived from the Stokes-Einstein equation:

$$\mathrm{DP} = \frac{(\mathrm{D}_{monomer})^3}{(\mathrm{D}_{assembly})^3}$$

Equation S2. DP determination of supramolecular polymers.

The diffusion coefficients summarized in Table S3 were obtained from the previously reported experiments of acid-induced transimination of **1** and **4** carried out on solutions in which **1**, **4**, and TFA were mixed in a 1 : 2 : 2 ratio, and that were prepared at different concentrations of **1**.

Table S3. Diffusion coefficient values obtained from DOSY analysis for **6** from acid-induced transimination of **1** and **4** (experiments carried out on solutions of **1**, **4**, and TFA mixed in a 1 : 2 : 2 ratio, at different concentrations of **1**). In particular, the Peak fit mode was used to obtain accurate and homogeneous values of the diffusion coefficients.

Concentration of imines	Diffusion Coefficient
50 mM	$2.57\cdot10^{-6}$ cm^2/s
100 mM	$1.39\cdot10^{-6}$ cm^2/s
150 mM	$9.34\cdot10^{-7}$ cm^2/s
175 mM	$6.48\cdot10^{-7}$ cm^2/s
200 mM	$5.19\cdot10^{-7}$ cm^2/s

The value of $2.57\cdot10^{-6}$ cm^2/s obtained for the dynamic library of 50 mM **1** and 100 mM **4** after TFA stoichiometric addition (100 mM) was adopted as $\mathrm{D}_{monomer}$, since in this case the supramolecular monomer (**6**•**3**•2H$^+$) is expected to be the main component in solution. This $\mathrm{D}_{monomer}$ value was used to calculate the DP values in Table S4.

Table S4. Diffusion coefficient values obtained from DOSY analysis of **6** and related DP of the assemblies obtained from acid-induced transimination of **1** and **4** carried out on solutions in which **1**, **4**, and TFA were mixed in a 1 : 2 : 2 ratio, and at different concentrations of **1**.

Concentration of imines	Diffusion Coefficient	DP
50 mM	$2.57\cdot10^{-6}$ cm^2/s	1
100 mM	$1.39\cdot10^{-6}$ cm^2/s	6
150 mM	$9.34\cdot10^{-7}$ cm^2/s	21
175 mM	$6.48\cdot10^{-7}$ cm^2/s	62
200 mM	$5.19\cdot10^{-7}$ cm^2/s	121

^{1}H-NMR spectra of 3, 3•2H$^+$, 3•2H$^+$ with 4, and of a 1:2:2 mixture of CCM 1, 4, and TFA

a) In a 4 mL vial 3.5 µL of **3** (0.03 mmol) were dissolved in 600 µL of $CDCl_3$ / CD_3OD 3:1.
b) In a 4 mL vial 3.5 µL of **3** (0.03 mmol) were dissolved in 500 µL of $CDCl_3$ / CD_3OD 3:1. Then, 100 µL of a 600 mM TFA solution (0.06 mmol) in $CDCl_3$ / CD_3OD 3:1 were added.
c) In a 4 mL vial 3.5 µL of **3** (0.03 mmol) were dissolved in 400 µL of $CDCl_3$ / CD_3OD 3:1. Then, 100 µL of 600 mM TFA (0.06 mmol) and 100 µL of 600 mM **4** (0.06 mmol) solutions in $CDCl_3$ / CD_3OD 3:1 were added.

^{1}H-NMR spectra were recorded and compared with the spectrum (d) obtained at 200 mM CCM **1**, 400 mM **4**, and 400 mM TFA, in $CDCl_3$ / CD_3OD 3:1.

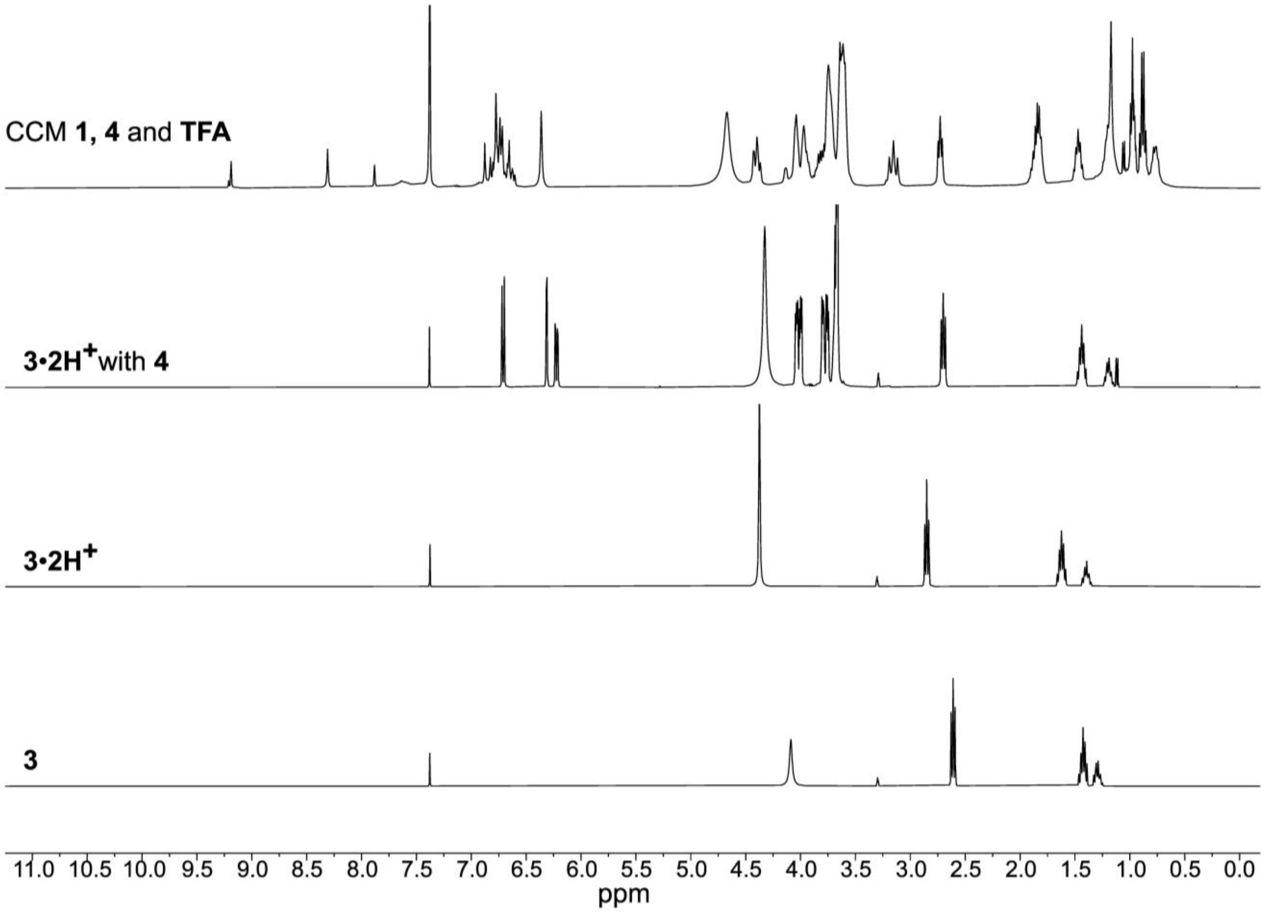

Figure S41. ^{1}H-NMR ($CDCl_3$ / CD_3OD 3:1). From bottom to top: a) 50 mM **3**; b) 50 mM **3**, and 100 mM TFA; c) 50 mM **3**, 100 mM **4**, and 100 mM TFA; d) 200 mM CCM **1**, 400 mM **4**, and 400 mM TFA.

Acid-induced transimination of 300 mM CP 2 and 600 mM 4, in a 600 mM TFA solution

In a 4 mL vial 38.6 mg of CP **2** (obtained at 500 mM monomer concentration, 0.180 mmol) were dissolved in 600 µL of a 600 mM solution of **4** (0.360 mmol) in $CDCl_3$ / CD_3OD 3:1. The mixture was sonicated for 1 minute in order to dissolve the polymer. Once a homogeneous solution was obtained, 27.6 µL of TFA (0.360 mmol) were added, and the mixture was investigated by 1D and 2D-DOSY ^{1}H-NMR.

^{1}H-NMR spectrum of a 300 mM CP 2, 600 mM 4, and 600 mM TFA solution

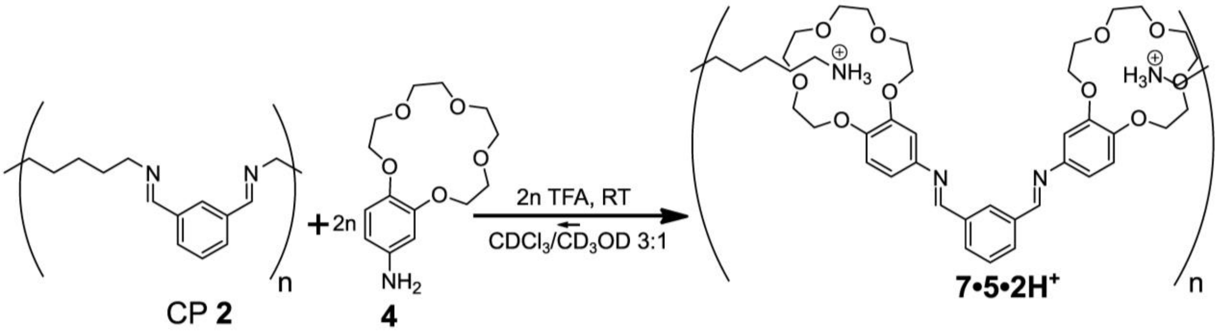

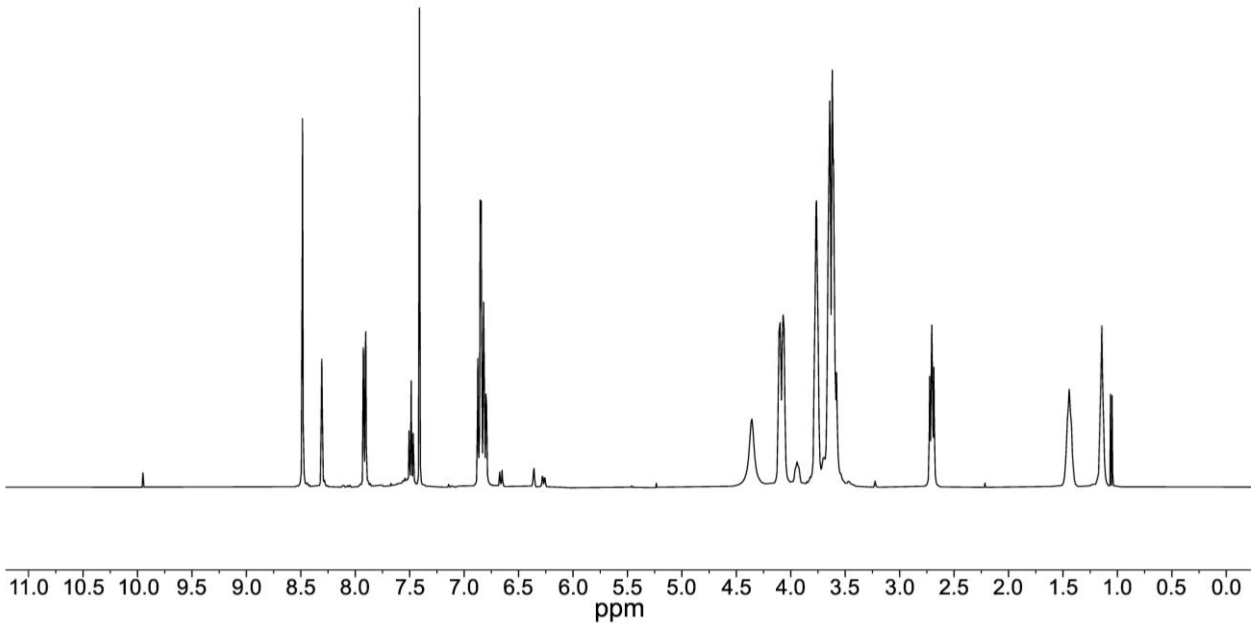

Figure S42. ^{1}H-NMR ($CDCl_3$ / CD_3OD 3:1) of a 300 mM CP **2**, 600 mM **4**, and 600 mM TFA solution.

DOSY spectrum (Bayesian mode) of a 300 mM CP 2, 600 mM 4, and 600 mM TFA solution

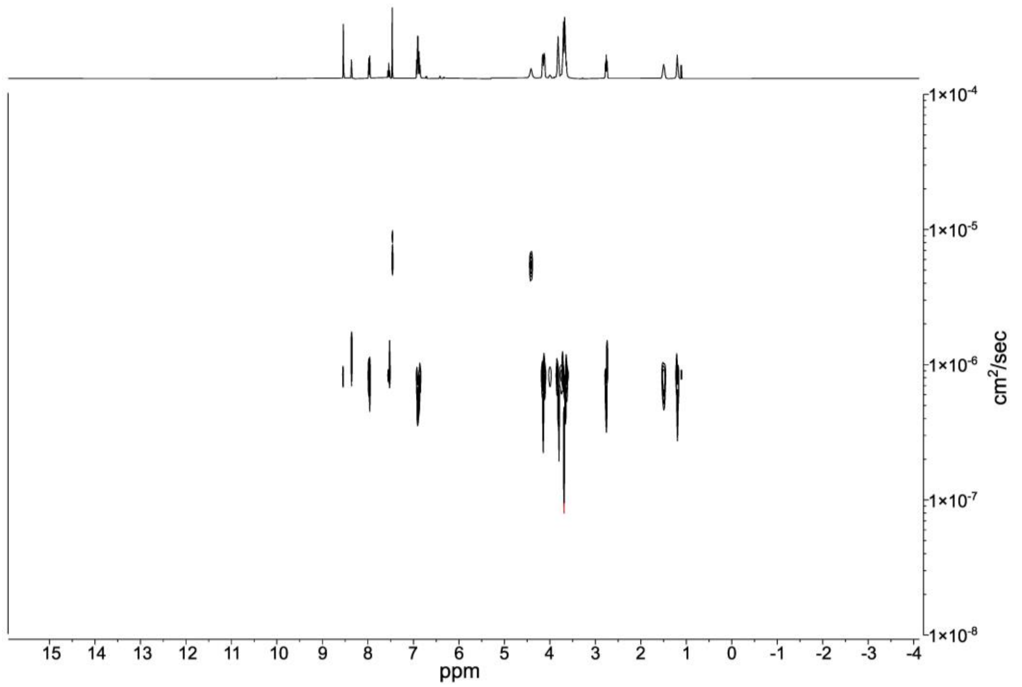

Figure S43. DOSY spectrum (Bayesian mode), recorded in $CDCl_3$ / CD_3OD 3:1 at RT, of a 300 mM CP **2**, 600 mM **4**, and 600 mM TFA solution.

DOSY spectrum (Peak fit mode) of a 300 mM CP 2, 600 mM 4, and 600 mM TFA solution

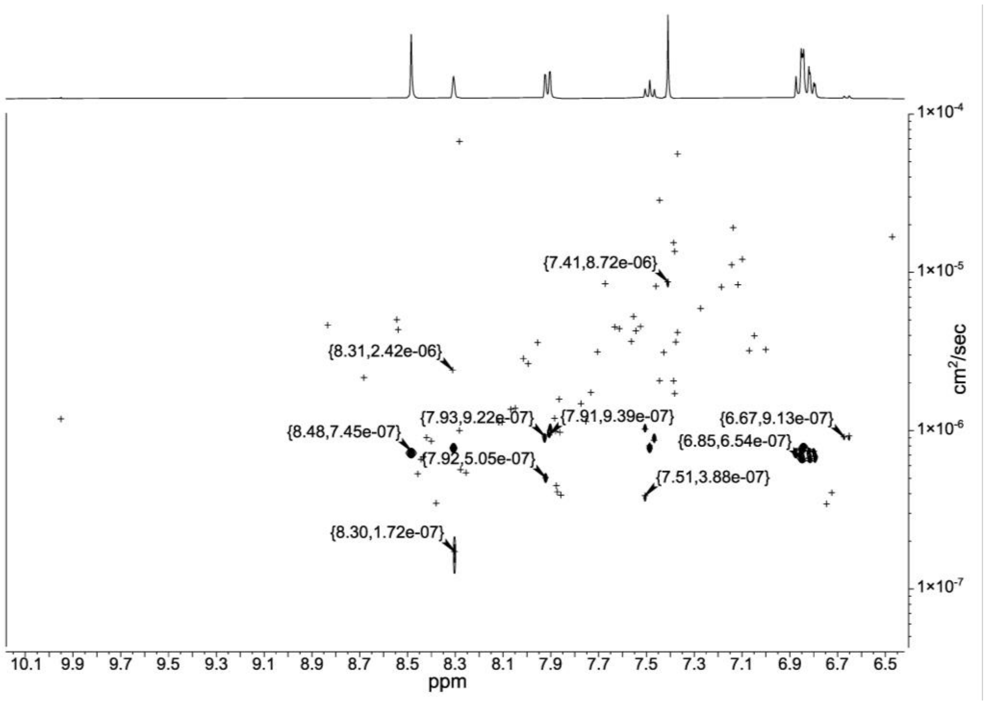

Figure S44. DOSY spectrum (Peak fit mode) of a 300 mM CP **2**, 600 mM **4**, and 600 mM TFA solution, recorded in $CDCl_3$ / CD_3OD 3:1 at RT.

From Peak fit mode the diffusion coefficient value $7.45 \cdot 10^{-7}$ cm^2/s was obtained for **7**.

Acid-induced transimination of 250 mM CP 2 and 500 mM 4, in a 500 mM TFA solution

In a 4 mL vial 32.1 mg of CP **2** (obtained at 500 mM, 0.150 mmol) were dissolved in 600 µL of a 500 mM solution of **4** (0.300 mmol) in $CDCl_3$ / CD_3OD 3:1. The mixture was sonicated for 1 minute in order to dissolve the polymer. Once a homogeneous solution was obtained, 23.0 µL of TFA (0.300 mmol) were added, and the mixture was investigated by 1D and 2D-DOSY ^{1}H-NMR.

^{1}H-NMR spectrum of a 250 mM CP 2, 500 mM 4, and 500 mM TFA solution

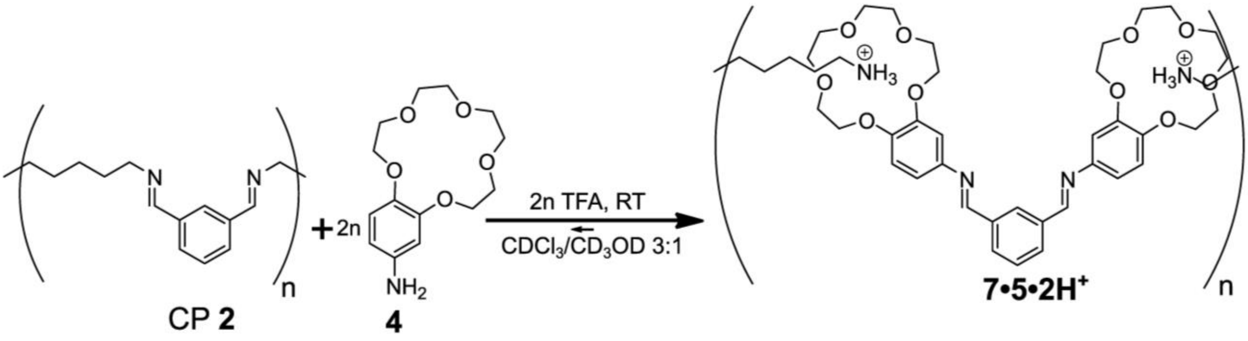

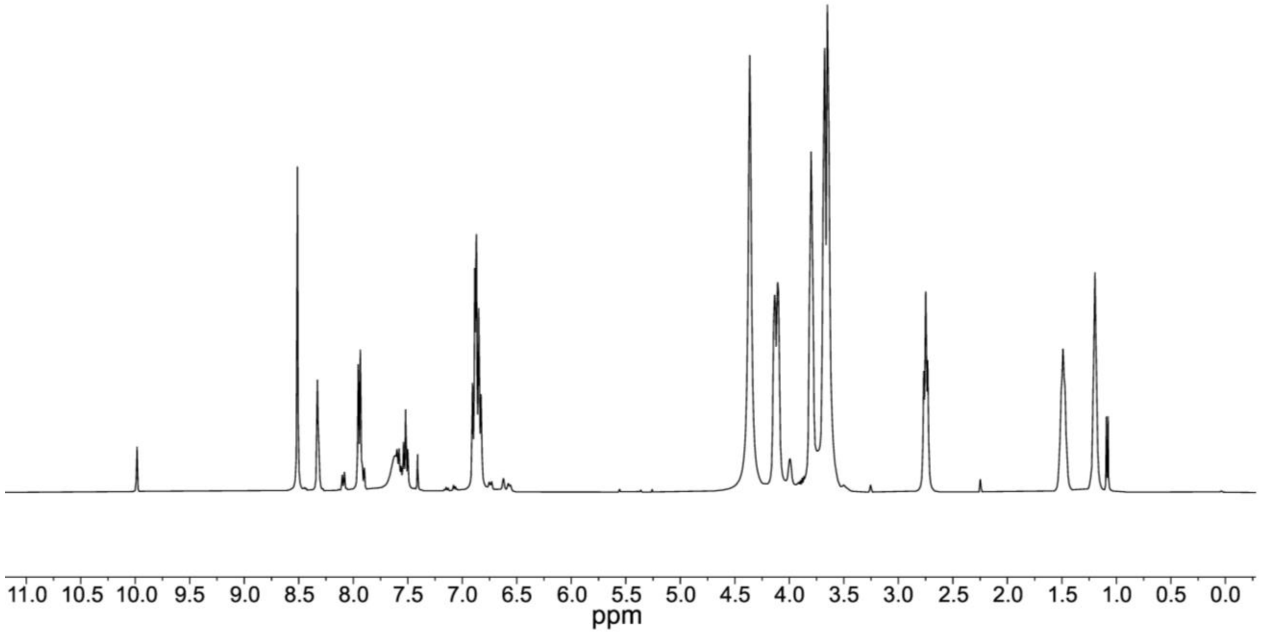

Figure S45. ^{1}H-NMR ($CDCl_3$ / CD_3OD 3:1) of a 250 mM CP **2**, 500 mM **4**, and 500 mM TFA solution.

DOSY spectrum (Bayesian mode) of a 250 mM CP 2, 500 mM 4, and 500 mM TFA solution

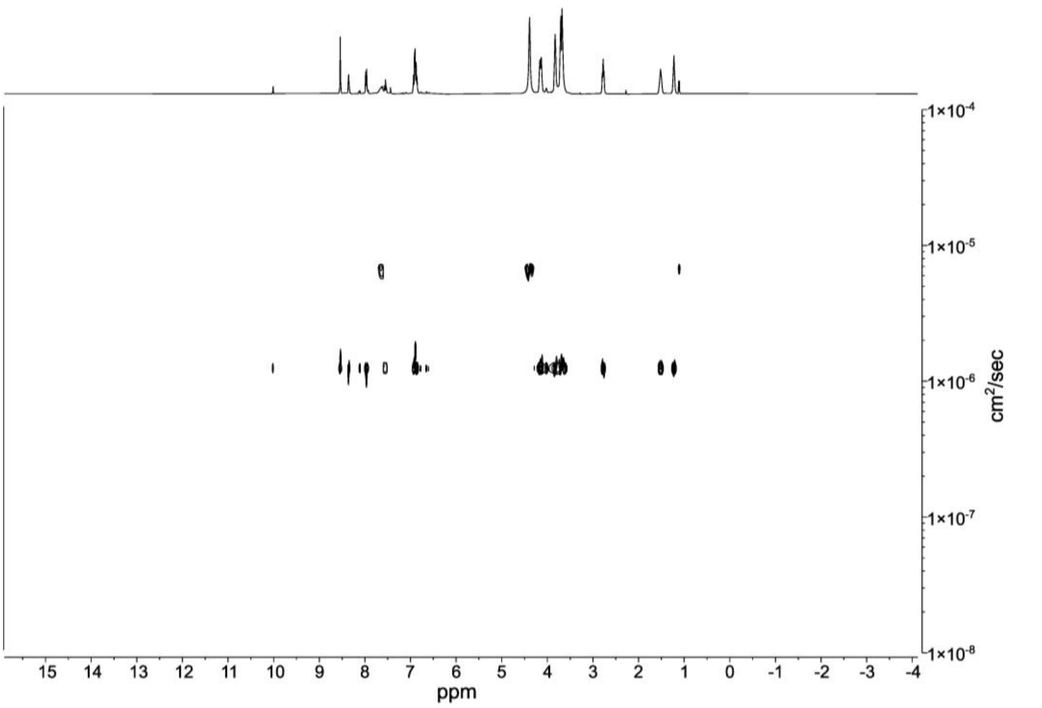

Figure S46. DOSY spectrum (Bayesian mode), recorded in $CDCl_3$ / CD_3OD 3:1 at RT, of a 250 mM CP **2**, 500 mM **4**, and 500 mM TFA solution.

DOSY spectrum (Peak fit mode) of a 250 mM CP 2, 500 mM 4, and 500 mM TFA solution

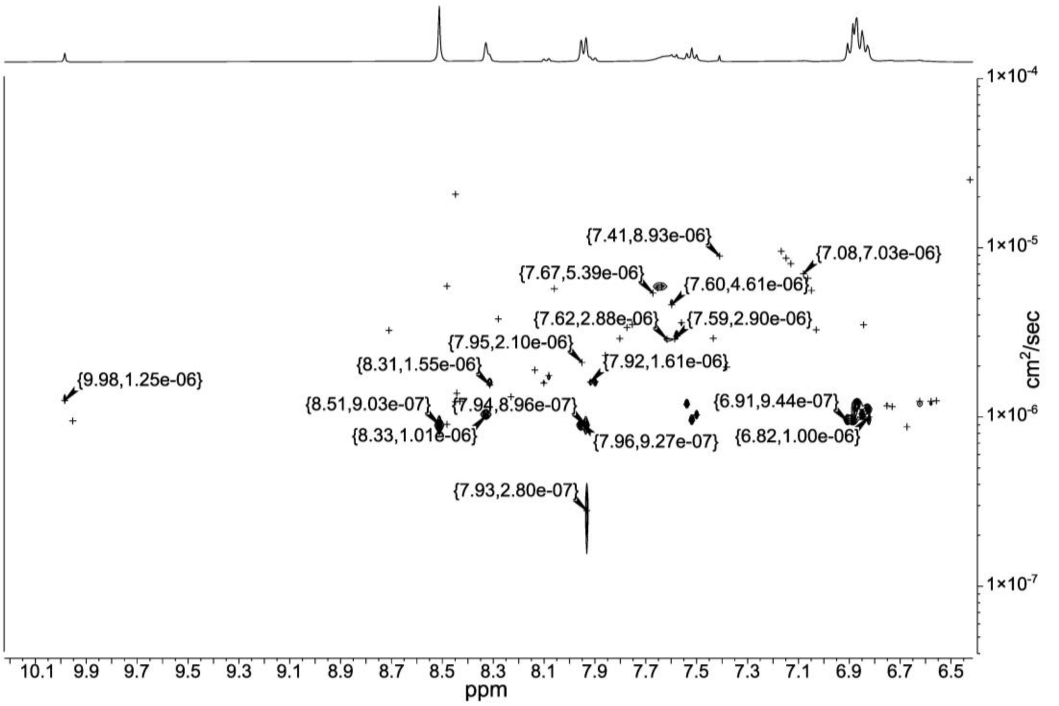

Figure S47. DOSY spectrum (Peak fit mode) of a 250 mM CP **2**, 500 mM **4**, and 500 mM TFA solution, recorded in $CDCl_3$ / CD_3OD 3:1 at RT.

From Peak fit mode the diffusion coefficient value $9.03 \cdot 10^{-7}$ cm^2/s was obtained for **7**.

Acid-induced transimination of 200 mM CP 2 and 400 mM 4, in a 400 mM TFA solution

In a 4 mL vial 25.7 mg of CP **2** (obtained at 500 mM monomer concentration, 0.120 mmol) were dissolved in 600 µL of a 400 mM solution of **4** (0.240 mmol) in $CDCl_3$ / CD_3OD 3:1. The mixture was sonicated for 1 minute in order to dissolve the polymer. Once a homogeneous solution was obtained, 18.4 µL of TFA (0.240 mmol) were added, and the mixture was studied by 1D and 2D-DOSY ^{1}H-NMR.

^{1}H-NMR spectrum of a 200 mM CP 2, 400 mM 4, and 400 mM TFA solution

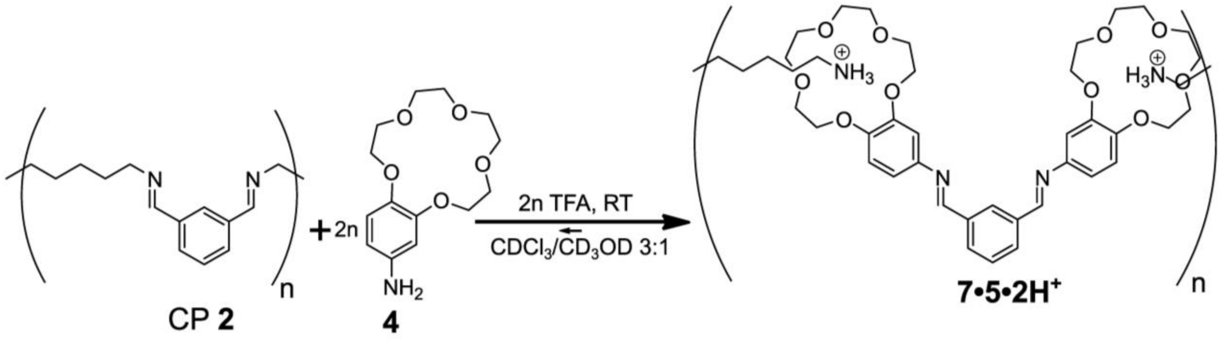

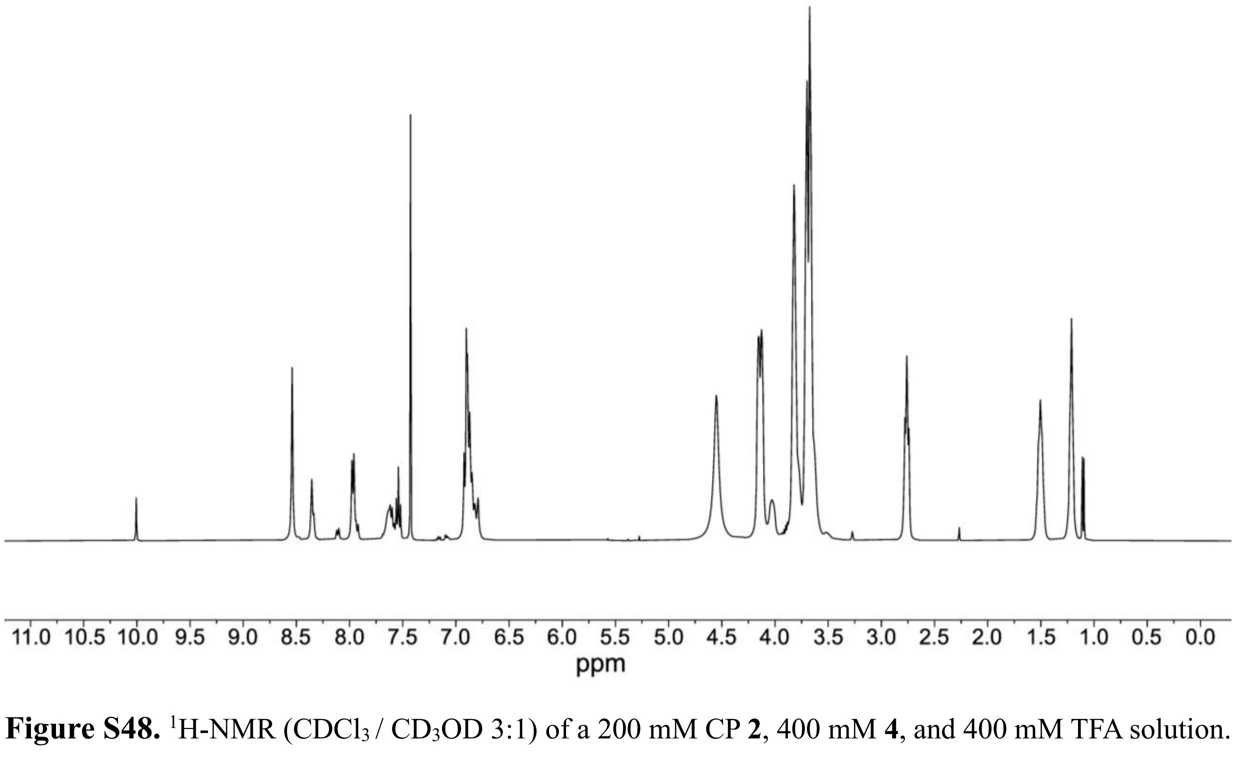

Figure S48. ^{1}H-NMR ($CDCl_3$ / CD_3OD 3:1) of a 200 mM CP **2**, 400 mM **4**, and 400 mM TFA solution.

DOSY spectrum (Bayesian mode) of a 200 mM CP 2, 400 mM 4, and 400 mM TFA solution

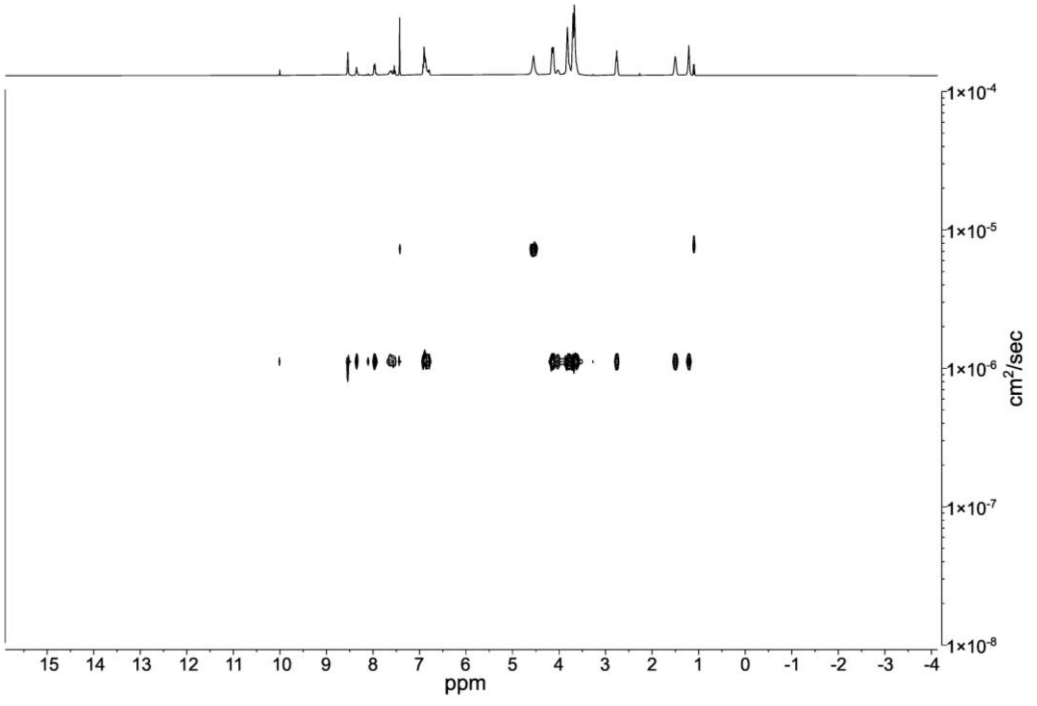

Figure S49. DOSY spectrum (Bayesian mode), recorded in $CDCl_3$ / CD_3OD 3:1 at RT, of a 200 mM CP **2**, 400 mM **4**, and 400 mM TFA solution.

DOSY spectrum (Peak fit mode) of a 200 mM CP 2, 400 mM 4, and 400 mM TFA solution

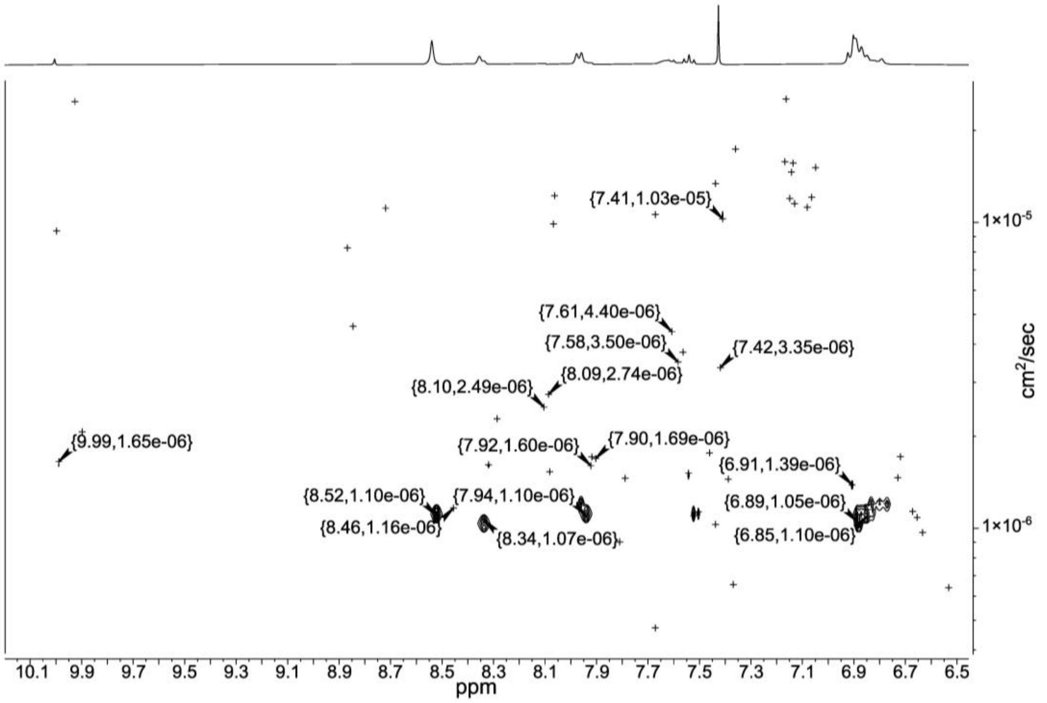

Figure S50. DOSY spectrum (Peak fit mode) of a 200 mM CP **2**, 400 mM **4**, and 400 mM TFA solution, recorded in $CDCl_3$ / CD_3OD 3:1 at RT.

From Peak fit mode the diffusion coefficient value $1.10 \cdot 10^{-6}$ cm^2/s was obtained for **7**.

Acid-induced transimination of 175 mM CP 2 and 350 mM 4, in a 350 mM TFA solution

In a 4 mL vial 22.5 mg of CP **2** (obtained at 500 mM monomer concentration, 0.105 mmol) were dissolved in 600 µL of a 350 mM solution of **4** (0.210 mmol) in $CDCl_3$ / CD_3OD 3:1. The mixture was sonicated for 1 minute in order to dissolve the polymer. Once a homogeneous solution was obtained, 16.1 µL of TFA (0.210 mmol) were added, and the mixture was investigated by 1D and 2D-DOSY ^{1}H-NMR.

^{1}H-NMR spectrum of a 175 mM CP 2, 350 mM 4, and 350 mM TFA solution

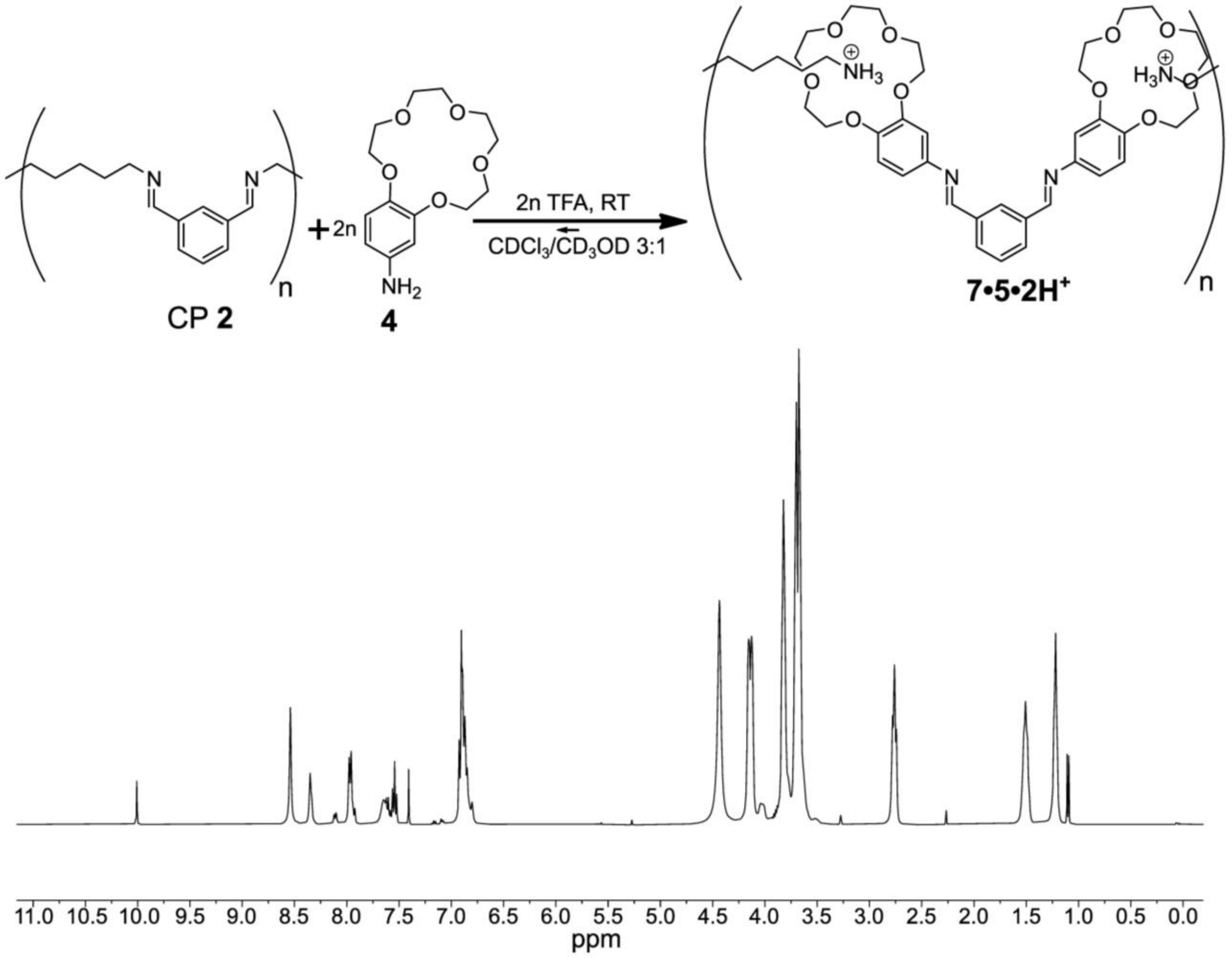

Figure S51. ^{1}H-NMR ($CDCl_3$ / CD_3OD 3:1) of a 175 mM CP **2**, 350 mM **4**, and 350 mM TFA solution.

DOSY spectrum (Bayesian mode) of a 175 mM CP 2, 350 mM 4, and 350 mM TFA solution

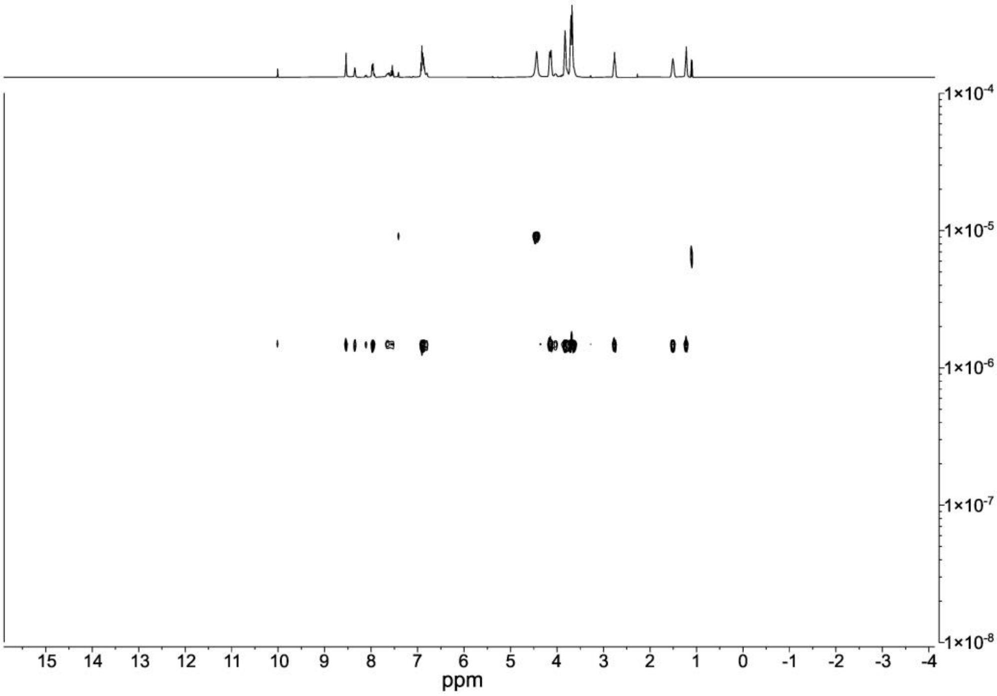

Figure S52. DOSY spectrum (Bayesian mode), recorded in $CDCl_3$ / CD_3OD 3:1 at RT, of a 175 mM CP **2**, 350 mM **4**, and 350 mM TFA solution.

DOSY spectrum (Peak fit mode) of a 175 mM CP 2, 350 mM 4, and 350 mM TFA solution

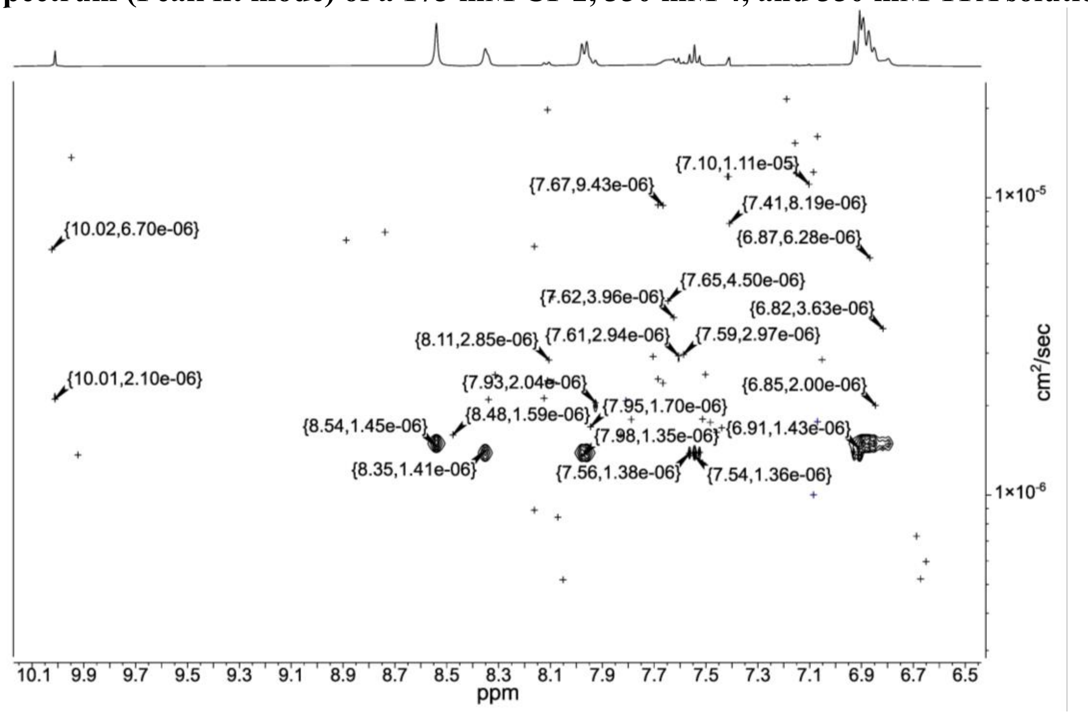

Figure S53. DOSY spectrum (Peak fit mode) of a 175 mM CP **2**, 350 mM **4**, and 250 mM TFA solution, recorded in $CDCl_3$ / CD_3OD 3:1 at RT.

From Peak fit mode the diffusion coefficient value $1.45 \cdot 10^{-6}$ cm^2/s was obtained for **7**.

Acid-induced transimination of 150 mM CP 2 and 300 mM 4, in a 300 mM TFA solution

In a 4 mL vial 19.3 mg of CP **2** (obtained at 500 mM monomer concentration, 0.090 mmol) were dissolved in 600 µL of a 300 mM solution of **4** (0.180 mmol) in $CDCl_3$ / CD_3OD 3:1. The mixture was sonicated for 1 minute in order to dissolve the polymer. Once a homogeneous solution was obtained, 13.8 µL of TFA (0.180 mmol) were added, and the mixture was studied by 1D and 2D-DOSY ^{1}H-NMR.

^{1}H-NMR spectrum of a 150 mM CP 2, 300 mM 4, and 300 mM TFA solution

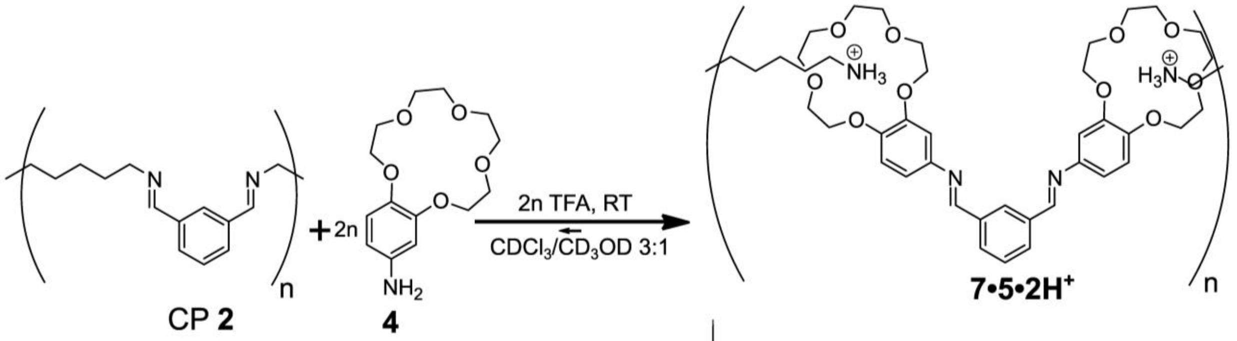

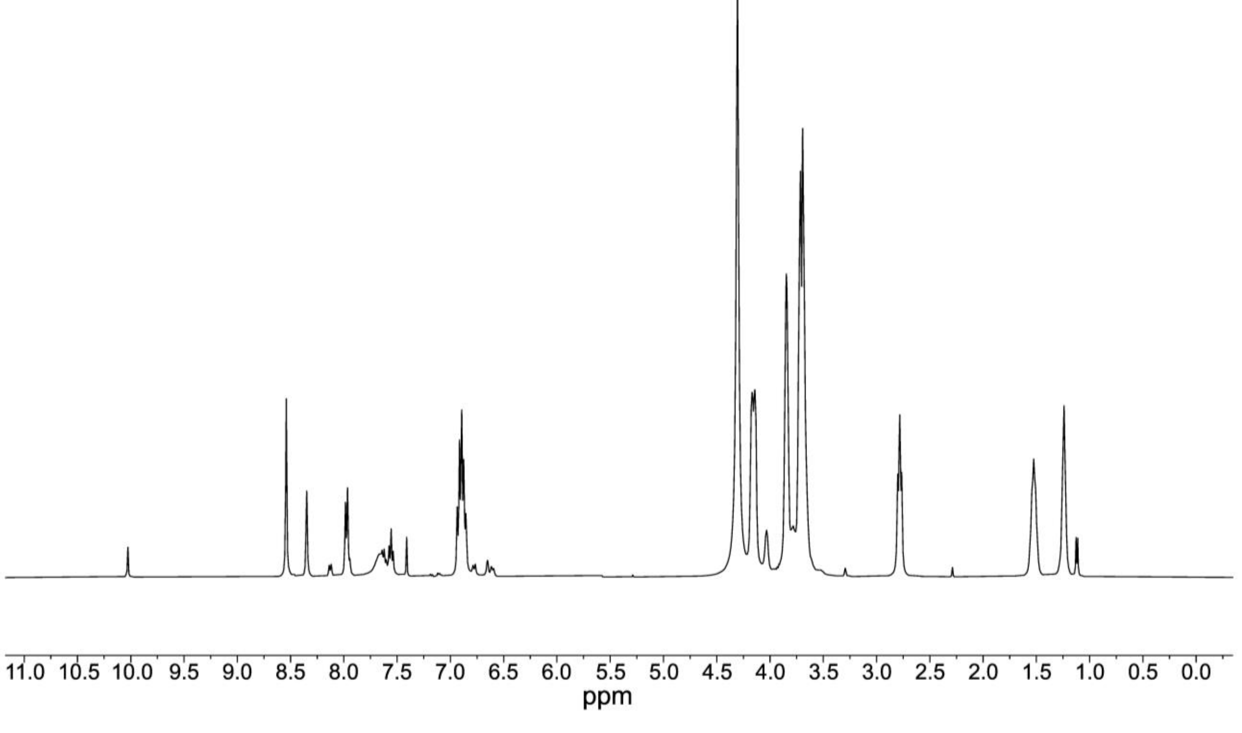

Figure S54. ^{1}H-NMR ($CDCl_3$ / CD_3OD 3:1) of a 150 mM CP **2**, 300 mM **4**, and 300 mM TFA solution.

DOSY spectrum (Bayesian mode) of a 150 mM CP 2, 300 mM 4, and 300 mM TFA solution

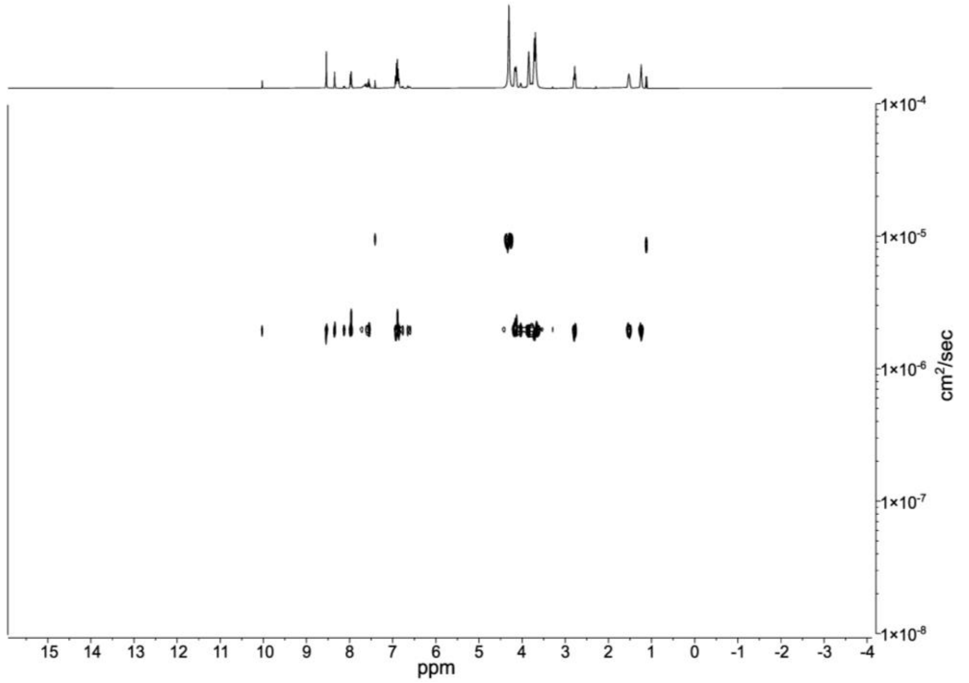

Figure S55. DOSY spectrum (Bayesian mode), recorded in $CDCl_3$ / CD_3OD 3:1 at RT, of a 150 mM CP **2**, 300 mM **4**, and 300 mM TFA solution.

DOSY spectrum (Peak fit mode) of a 150 mM CP 2, 300 mM 4, and 300 mM TFA solution

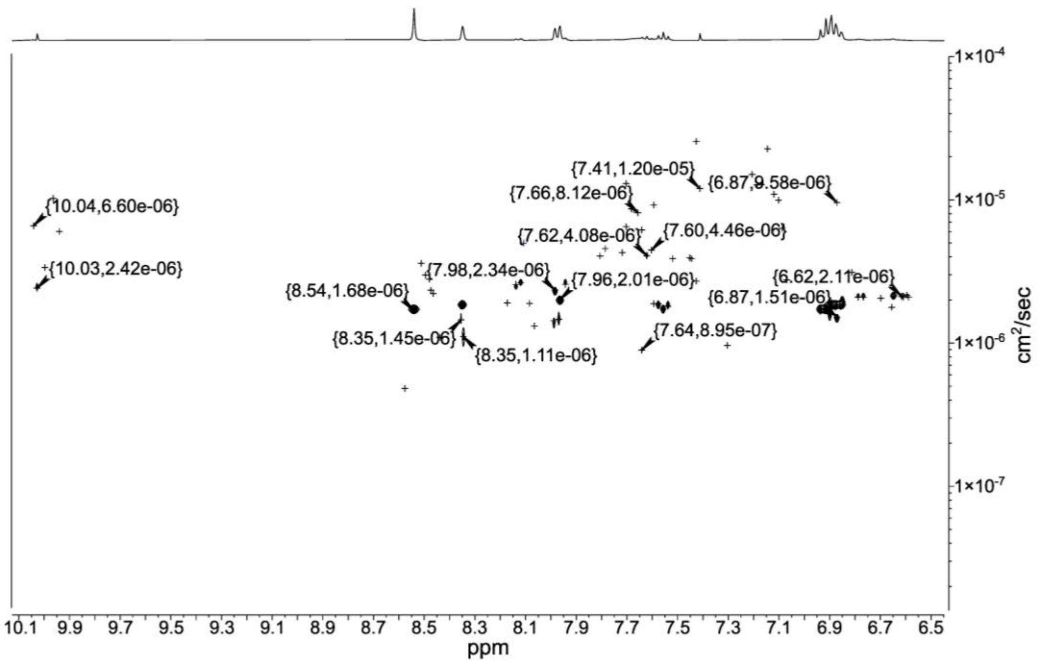

Figure S56. DOSY spectrum (Peak fit mode) of a 150 mM CP **2**, 300 mM **4**, and 300 mM TFA solution, recorded in $CDCl_3$ / CD_3OD 3:1 at RT.

From Peak fit mode the diffusion coefficient value $1.45 \cdot 10^{-6}$ cm^2/s was obtained for **7**.

Acid-induced transimination of 100 mM CP 2 and 200 mM 4, in a 200 mM TFA solution

In a 4 mL vial 300 µL of a 200 mM solution in $CDCl_3$ / CD_3OD 3:1 of CP **2** (obtained at 500 mM monomer concentration, 0.060 mmol) were added to 300 µL of a 400 mM solution of **4** (0.120 mmol) in $CDCl_3$ / CD_3OD 3:1. To this mixture 9.2 µL of TFA (0.120 mmol) were added, and the solution was studied by 1D and 2D-DOSY ^{1}H-NMR.

^{1}H-NMR spectrum of a 100 mM CP 2, 200 mM 4, and 200 mM TFA solution

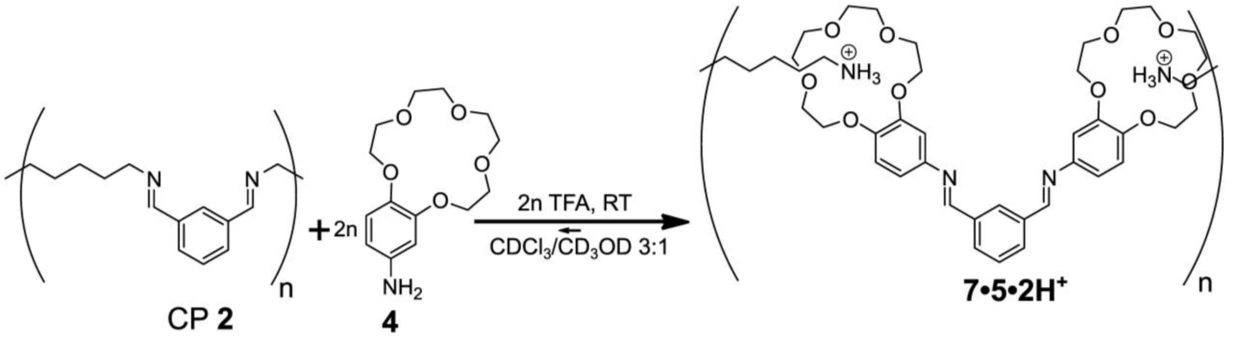

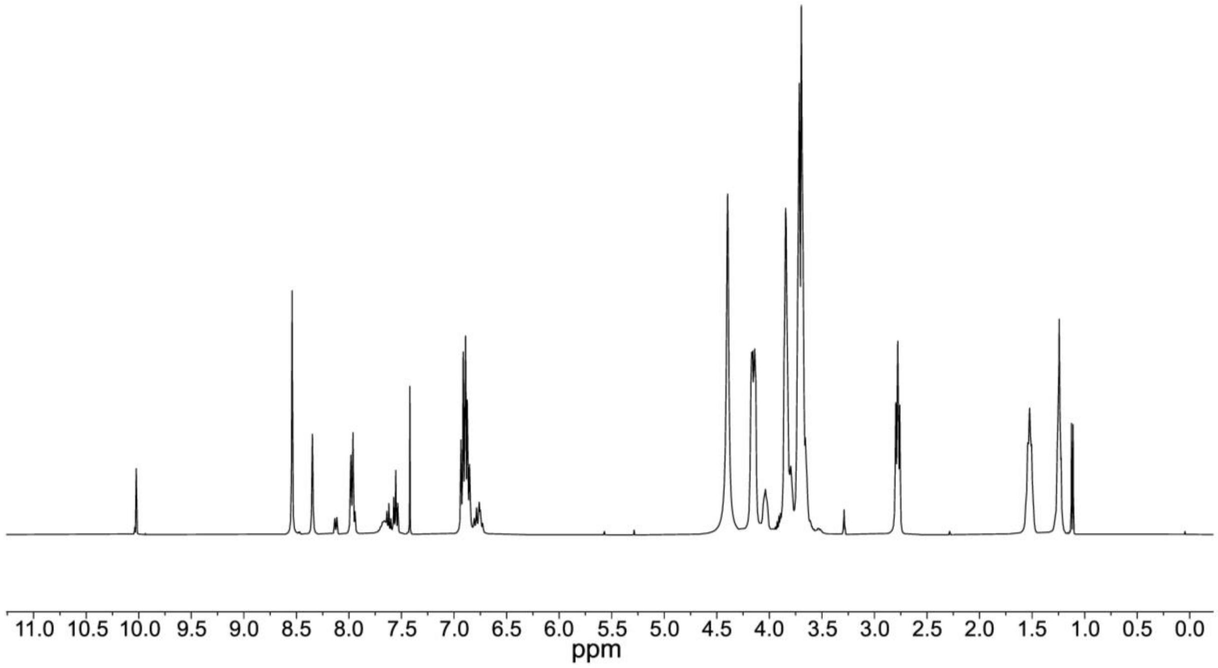

Figure S57. ^{1}H-NMR ($CDCl_3$ / CD_3OD 3:1) of a 100 mM CP **2**, 200 mM **4**, and 200 mM TFA solution.

DOSY spectrum (Bayesian mode) of a 100 mM CP 2, 200 mM 4, and 200 mM TFA solution

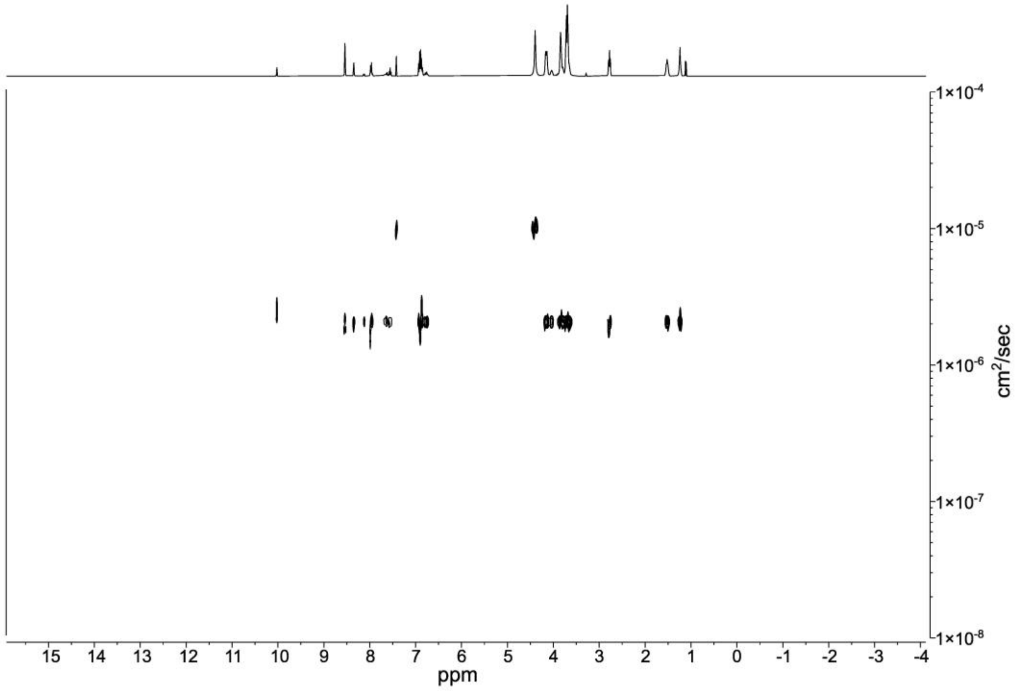

Figure S58. DOSY spectrum (Bayesian mode), recorded in $CDCl_3$ / CD_3OD 3:1 at RT, of a 100 mM CP **2**, 200 mM **4**, and 200 mM TFA solution.

DOSY spectrum (Peak fit mode) of a 100 mM CP 2, 200 mM 4, and 200 mM TFA solution

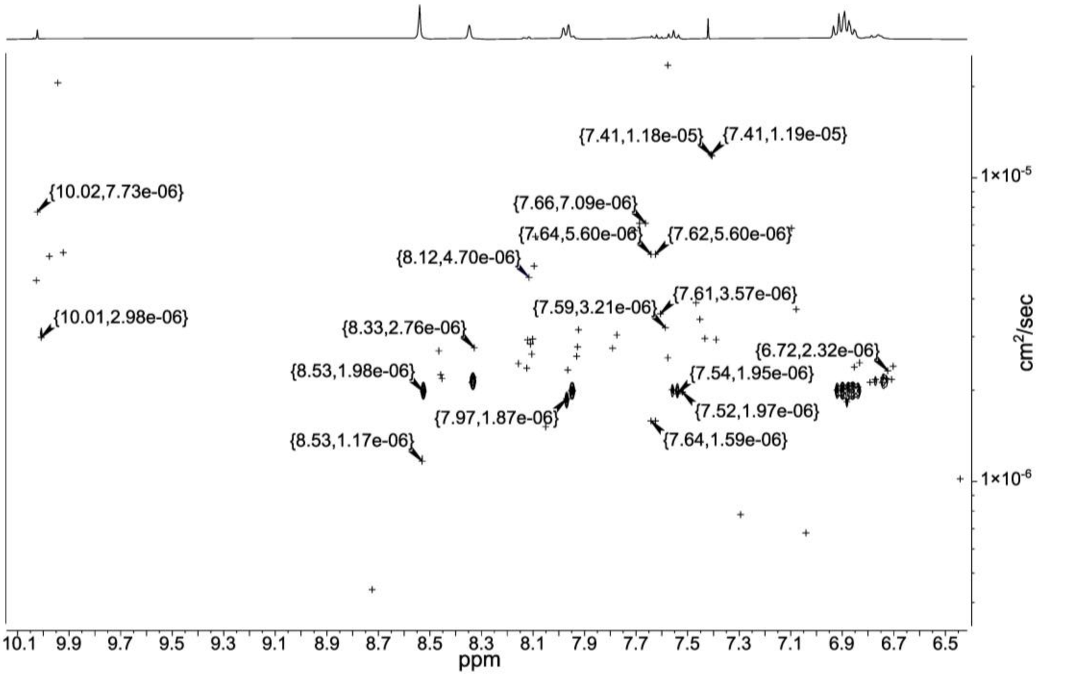

Figure S59. DOSY spectrum (Peak fit mode) of a 100 mM CP **2**, 200 mM **4**, and 200 mM TFA solution, recorded in $CDCl_3$ / CD_3OD 3:1 at RT.

From Peak fit mode the diffusion coefficient value $1.98 \cdot 10^{-6}$ cm^2/s was obtained for **7**.

Acid-induced transimination of 50 mM CP 2 and 100 mM 4, in a 100 mM TFA solution

In a NMR tube 300 µL of a 100 mM solution in $CDCl_3$ / CD_3OD 3:1 of CP **2** (obtained at 500 mM, 0.030 mmol) were added to 200 µL of a 300 mM solution of **4** (0.060 mmol) in $CDCl_3$ / CD_3OD 3:1. Then, 100 µL of a 600 mM TFA solution (0.060 mmol) in $CDCl_3$ / CD_3OD 3:1 were added, and the mixture was followed by 1D and 2D-DOSY ^{1}H-NMR.

^{1}H-NMR spectrum of a 50 mM CP 2, 100 mM 4, and 100 mM TFA solution

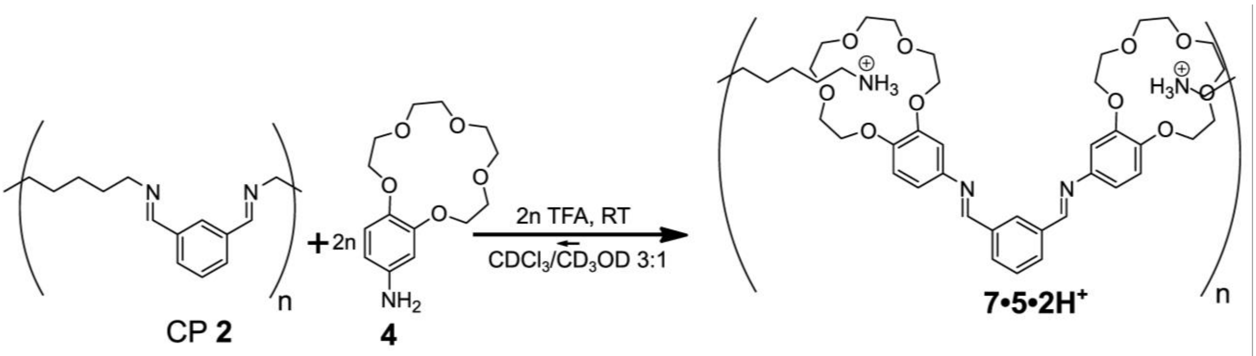

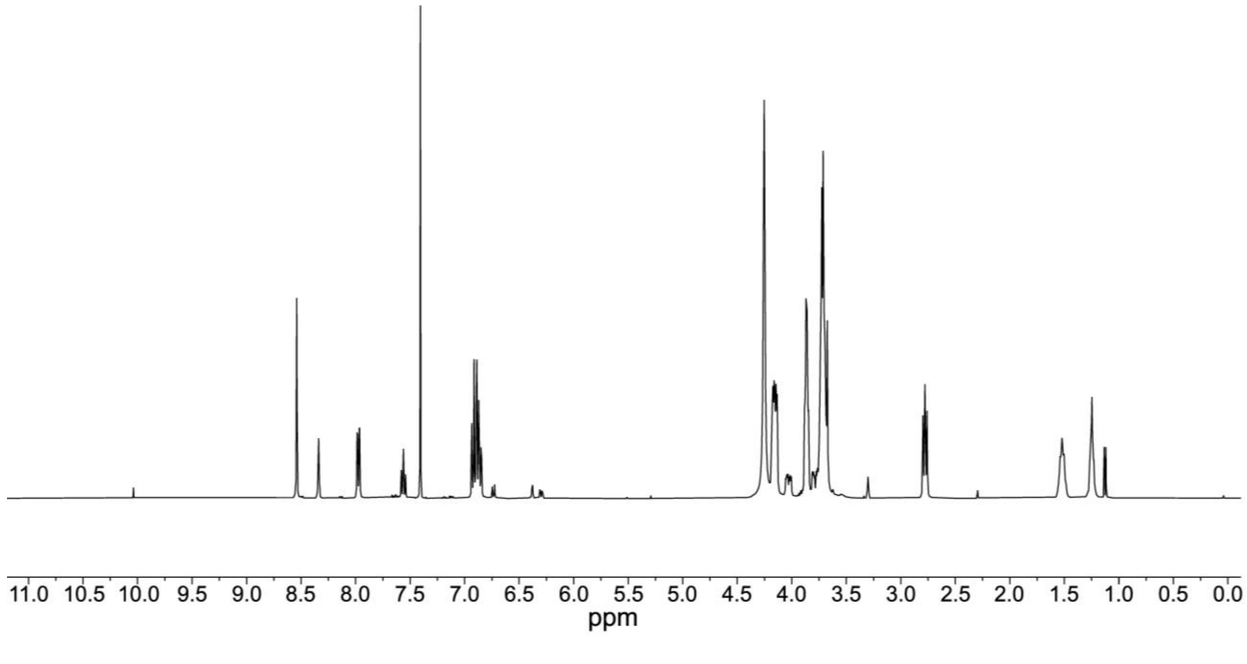

Figure S60. ^{1}H-NMR ($CDCl_3$ / CD_3OD 3:1) of a 50 mM CP **2**, 100 mM **4**, and 100 mM TFA solution.

DOSY spectrum (Bayesian mode) of a 50 mM CP 2, 100 mM 4, and 100 mM TFA solution

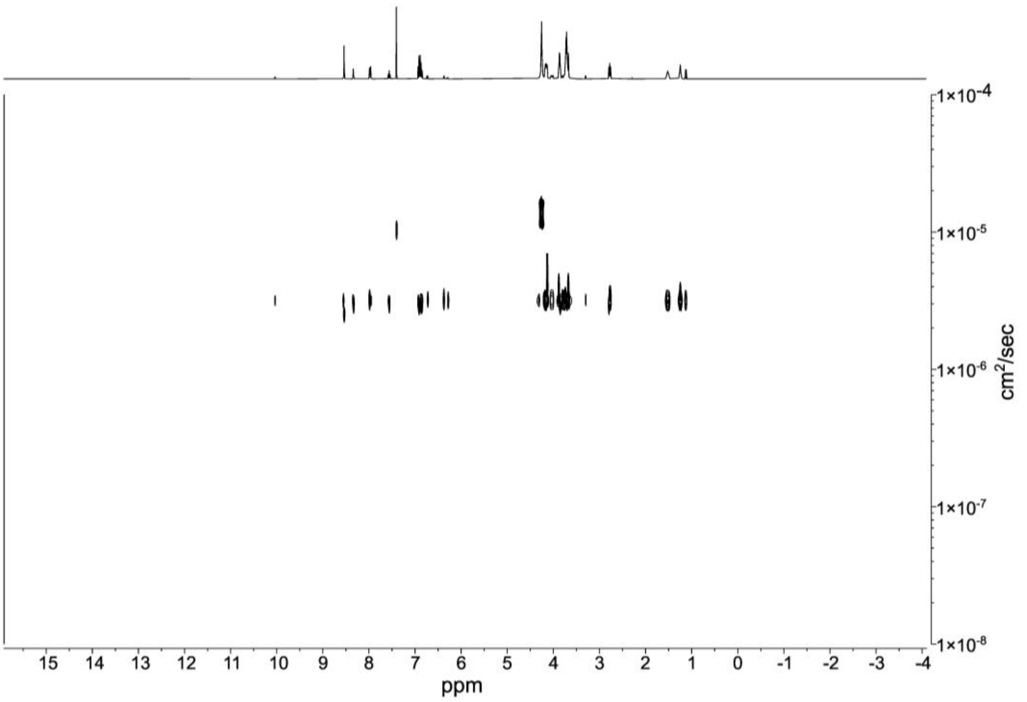

Figure S61. DOSY spectrum (Bayesian mode), recorded in $CDCl_3$ / CD_3OD 3:1 at RT, of a 50 mM CP **2**, 100 mM **4**, and 100 mM TFA solution

DOSY spectrum (Peak fit mode) of a 50 mM CP 2, 100 mM 4, and 100 mM TFA solution

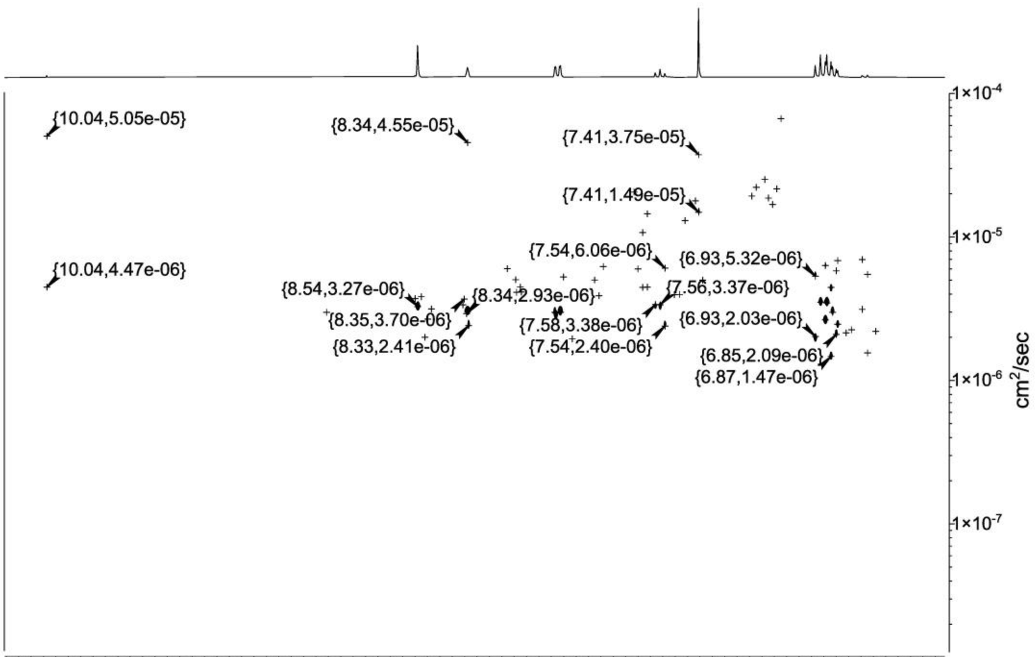

Figure S62. DOSY spectrum (Peak fit mode) of a 50 mM CP **2**, 100 mM **4**, and 100 mM TFA solution, recorded in $CDCl_3$ / CD_3OD 3:1 at RT.

From Peak fit mode the diffusion coefficient value $3.27 \cdot 10^{-6}$ cm^2/s was obtained for **7**.

Acid-induced transimination of 25 mM CP 2 and 50 mM 4, in a 50 mM TFA solution

In a NMR tube 300 µL of a 50 mM solution of CP **2** (obtained at 500 mM monomer concntration, 0.015 mmol) in $CDCl_3$ / CD_3OD 3:1, were added to 200 µL of a 150 mM solution of **4** (0.030 mmol) in $CDCl_3$ / CD_3OD 3:1. Then, 100 µL of a 300 mM TFA solution (0.030 mmol) in $CDCl_3$ / CD_3OD 3:1 were added, and the mixture was studied by 1D and 2D-DOSY ^{1}H-NMR.

^{1}H-NMR spectrum of a 25 mM CP 2, 50 mM 4, and 50 mM TFA solution

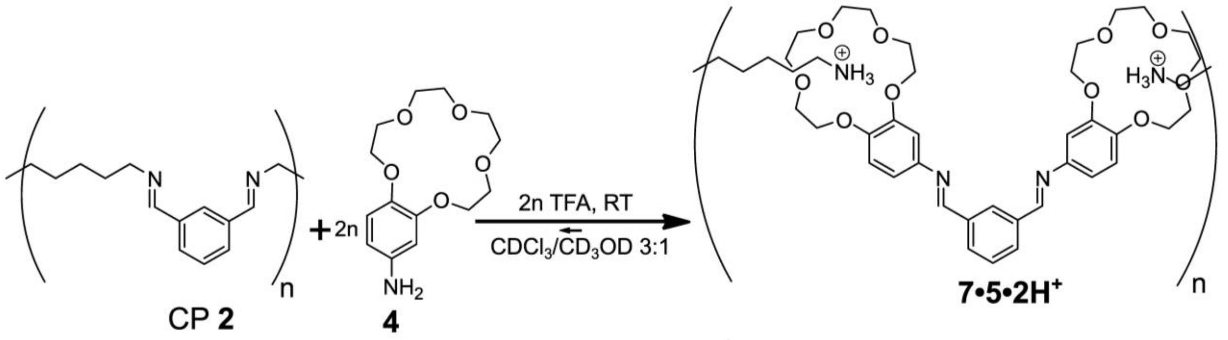

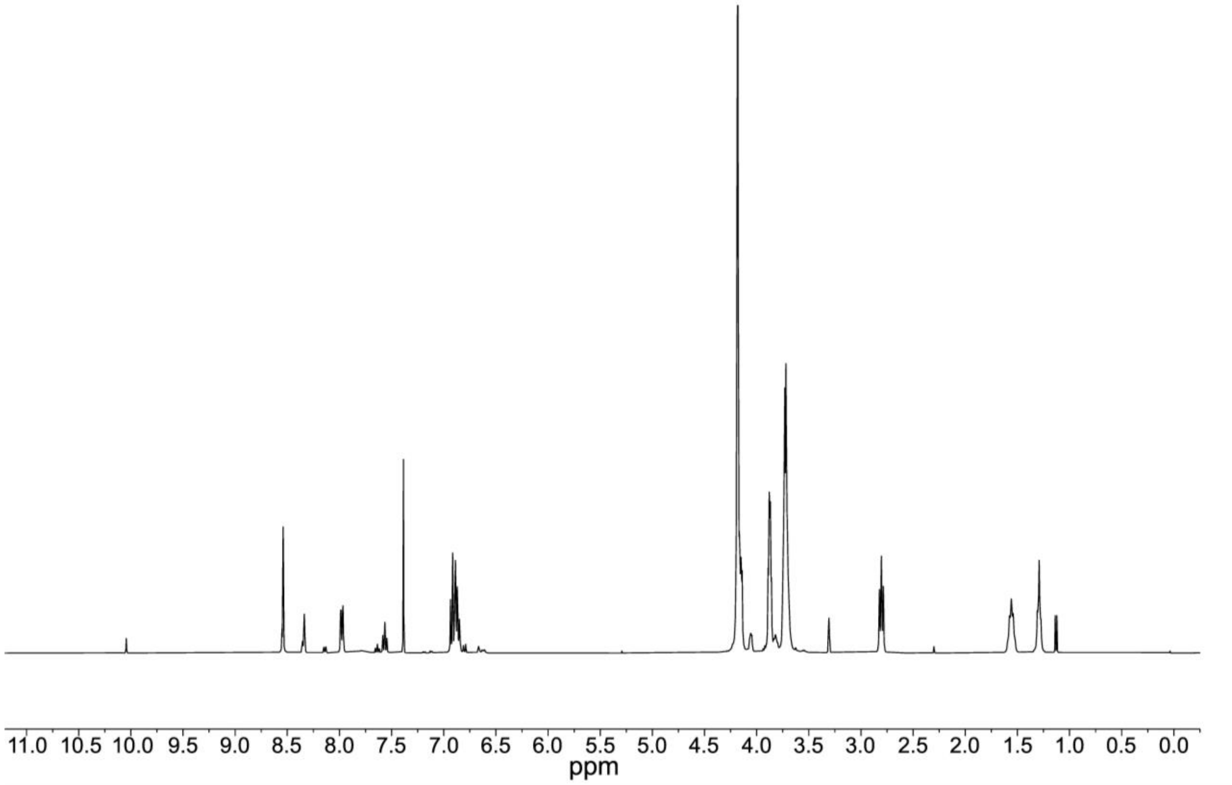

Figure S63. ^{1}H-NMR ($CDCl_3$ / CD_3OD 3:1) of a 25 mM CP **2**, 50 mM **4**, and 50 mM TFA solution.

DOSY spectrum (Bayesian mode) of a 25 mM CP 2, 50 mM 4, and 50 mM TFA solution

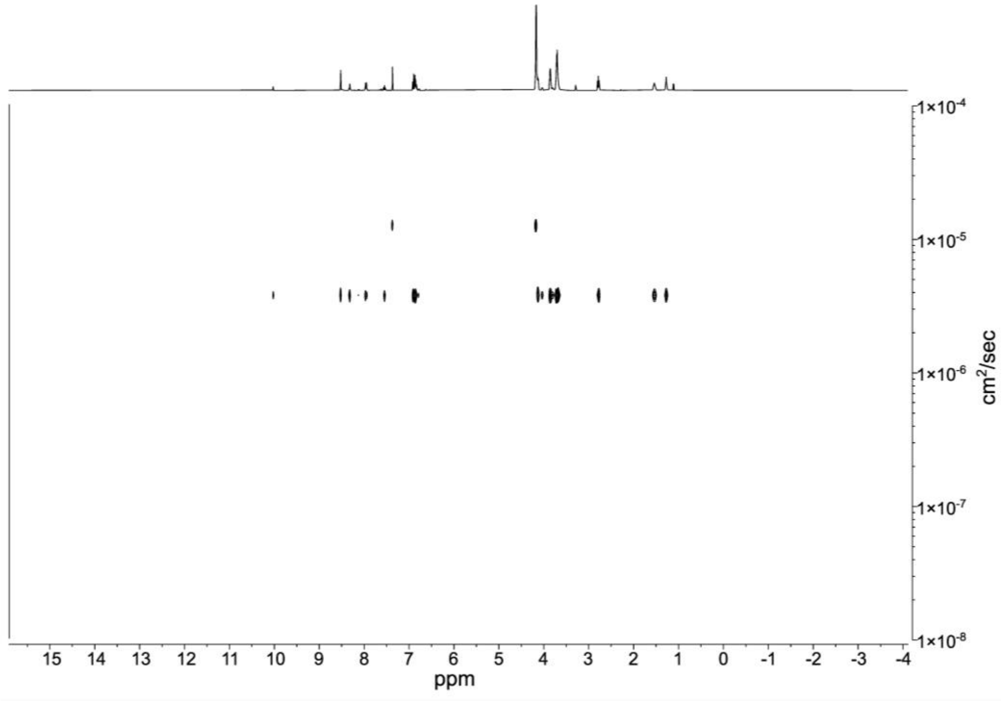

Figure S64. DOSY spectrum (Bayesian mode), recorded in $CDCl_3$ / CD_3OD 3:1 at RT, of a 25 mM CP **2**, 50 mM **4**, and 50 mM TFA solution.

DOSY spectrum (Peak fit mode) of a 25 mM CP 2, 50 mM 4, and 50 mM TFA solution

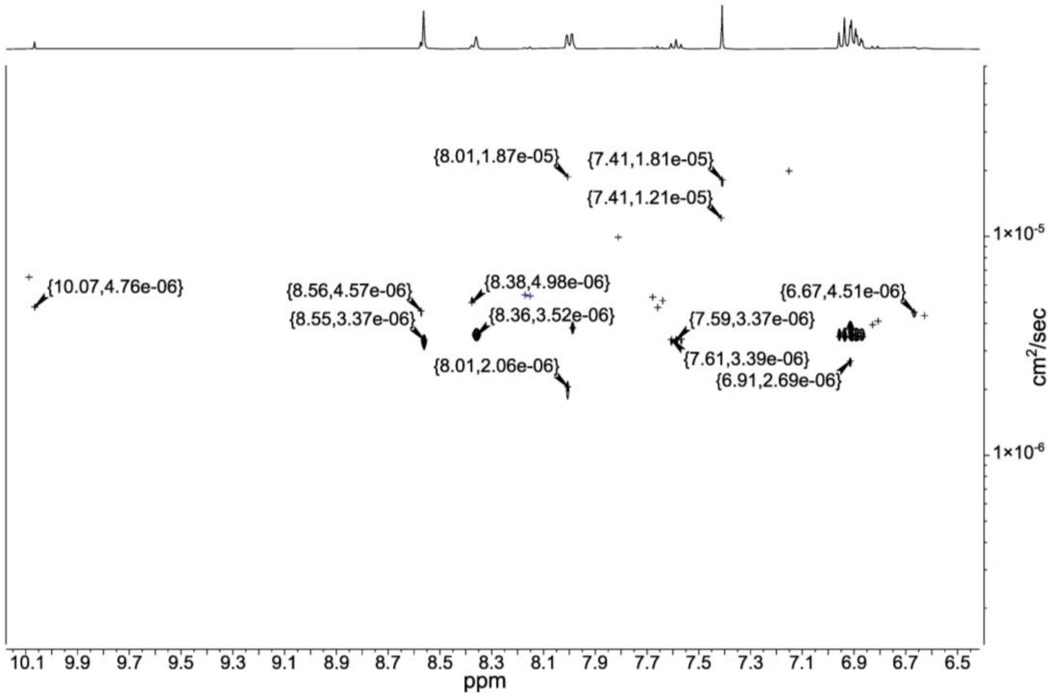

Figure S65. DOSY spectrum (Peak fit mode) of a 25 mM CP **2**, 50 mM **4**, and 50 mM TFA solution, recorded in $CDCl_3$ / CD_3OD 3:1 at RT.

From Peak fit mode the diffusion coefficient value $3.37 \cdot 10^{-6}$ cm^2/s was obtained for **7**.

Synthesis of monomer 7

In a 25 mL round bottom flask 33.5 mg (0.250 mmol) of isophthalaldehyde, were dissolved in 5 mL of toluene. Then, 141.7 mg (0.500 mmol) of **4** were added to the solution under stirring. The mixture was refluxed under stirring overnight. Once the mixture was cooled down, solvent was removed under vacuum and the mixture characterized by 1D and 2D-DOSY ^{1}H-NMR. ^{1}H-NMR analysis revealed a yield of 85% for **7** (Figure S66), with the presence of the monoaldehyde derivative of **7** as a side product (15%). ^{1}H NMR (400 MHz, CD_3OD) δ: 8.51 (s, 2H), 8.31 (q, J = 2.0 Hz, 1H), 7.96 (dd, J = 7.7, 1.7 Hz, 2H), 7.53 (t, J = 7.7 Hz, 1H), 6.93 – 6.73 (m, 6H), 4.17 – 4.08 (m, 8H), 3.87 (q, J = 4.9 Hz, 8H), 3.72 (d, J = 1.8 Hz, 16H).

^{1}H-NMR spectrum of monomer 7

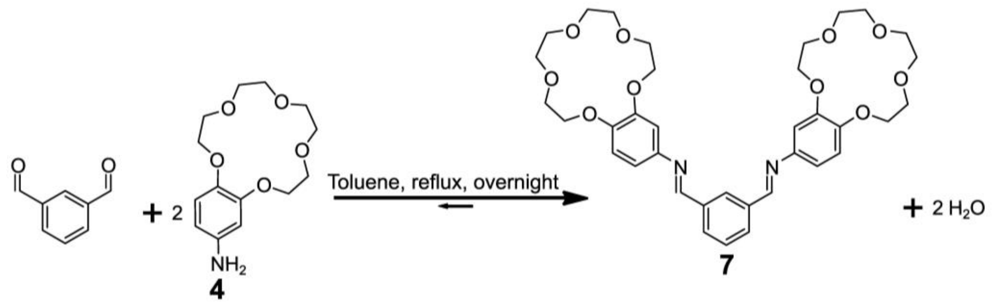

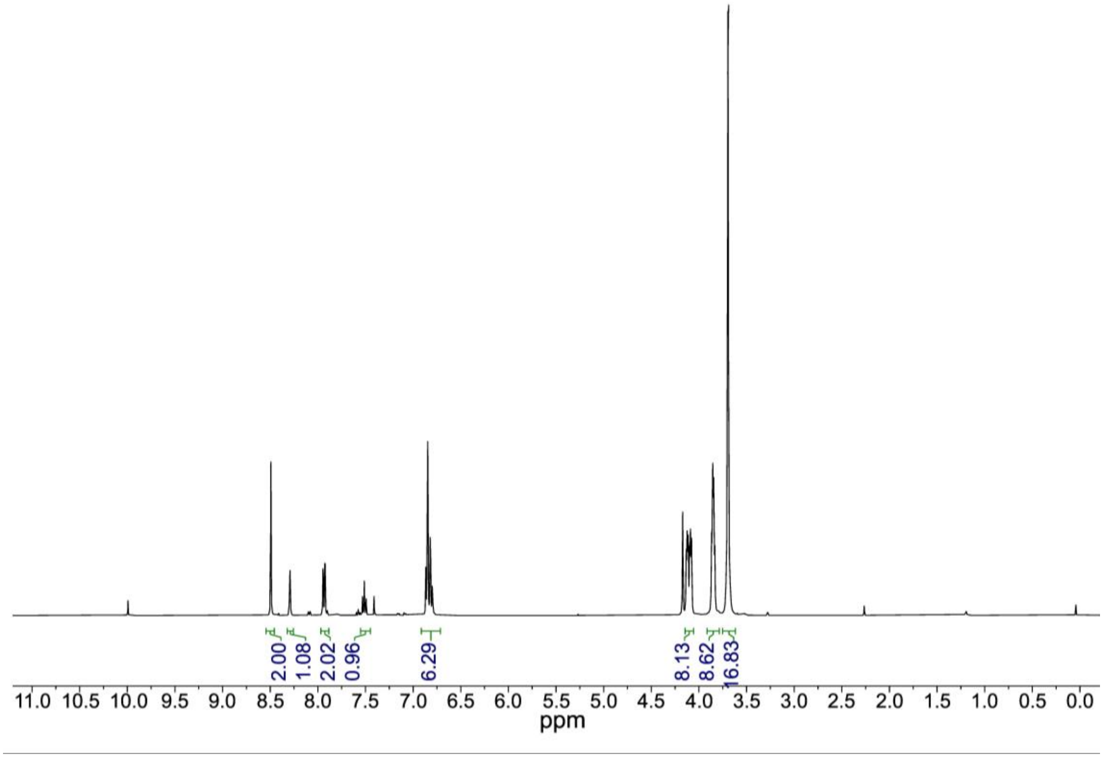

Figure S66. Preparation of monomer **7** from reaction of 50 mM isophthalaldehyde with 100 mM **4** in toluene at reflux, (^{1}H-NMR; $CDCl_3$ / CD_3OD 3:1).

DP determination of SP-β

To determine the DP for the assemblies of monomer **7,** obtained by treating different concentration mixtures of CP **2** / **4** 1:2 with a stoichiometric amount of TFA, we used Equation S2 (page S34). In order to measure the diffusion coefficient of monomer **7**, we equilibrated isophthalaldehyde with **4**, obtaining monomer **7** and its monoaldehydic counterpart. The diffusion coefficient of **7** was then determined by DOSY experiment, considering this imine a good approximation of the supramolecular monomeric unit [**7** + **5**•2H$^+$].

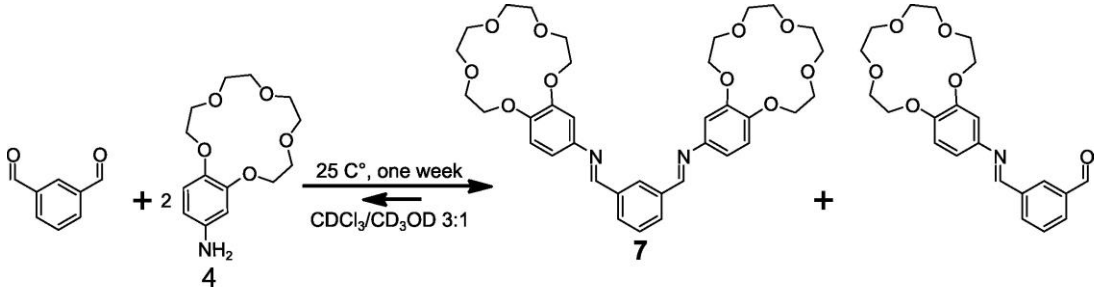

Figure S67. General scheme of the imine preparation by the condensation reaction of isophthalaldehyde with **4** at RT.

Sample preparation of monomer 7 DOSY experiment

In a NMR tube 300 μL of a 100 mM solution of isophthalaldehyde (0.030 mmol) in $CDCl_3$ / CD_3OD 3:1 were added to 300 μL of a 200 mM solution of **4** in $CDCl_3$ / CD_3OD 3:1. The mixture was let to equilibrate for one week and a DOSY experiment of the equilibrium mixture was recorded.

DOSY spectrum (Bayesian mode) of monomer 7

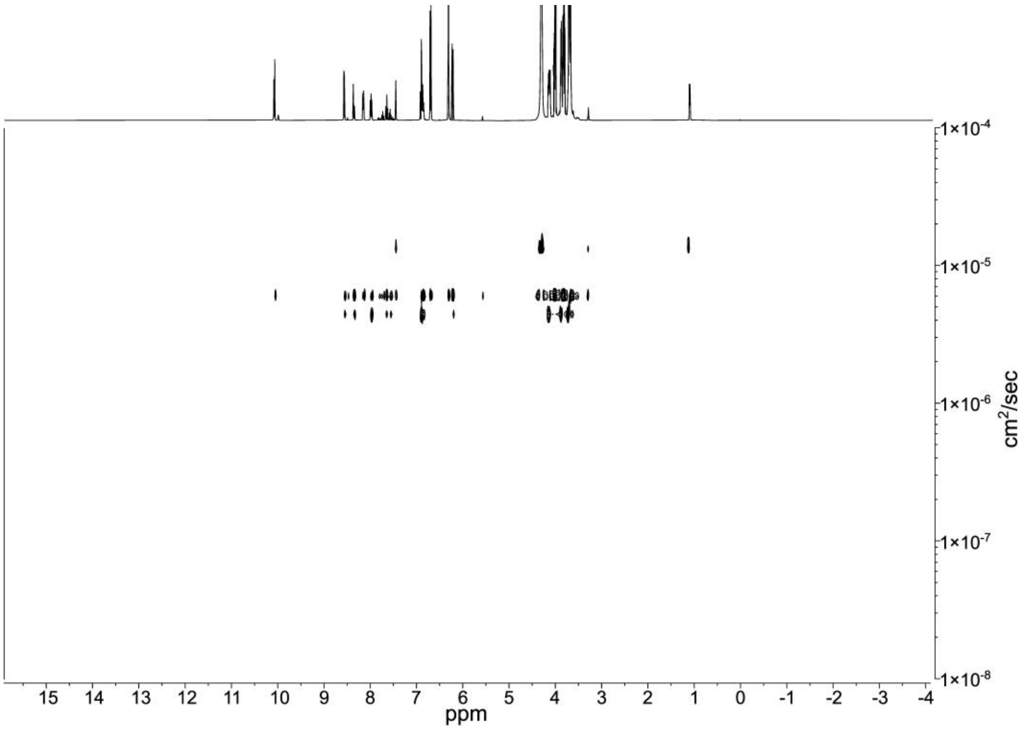

Figure S68. DOSY spectrum (Bayesian mode), of the equilibrium mixture of isophthalaldehyde and **4**, ($CDCl_3$ / CD_3OD 3:1; RT).

DOSY spectrum (Peak fit mode) of Monomer 7

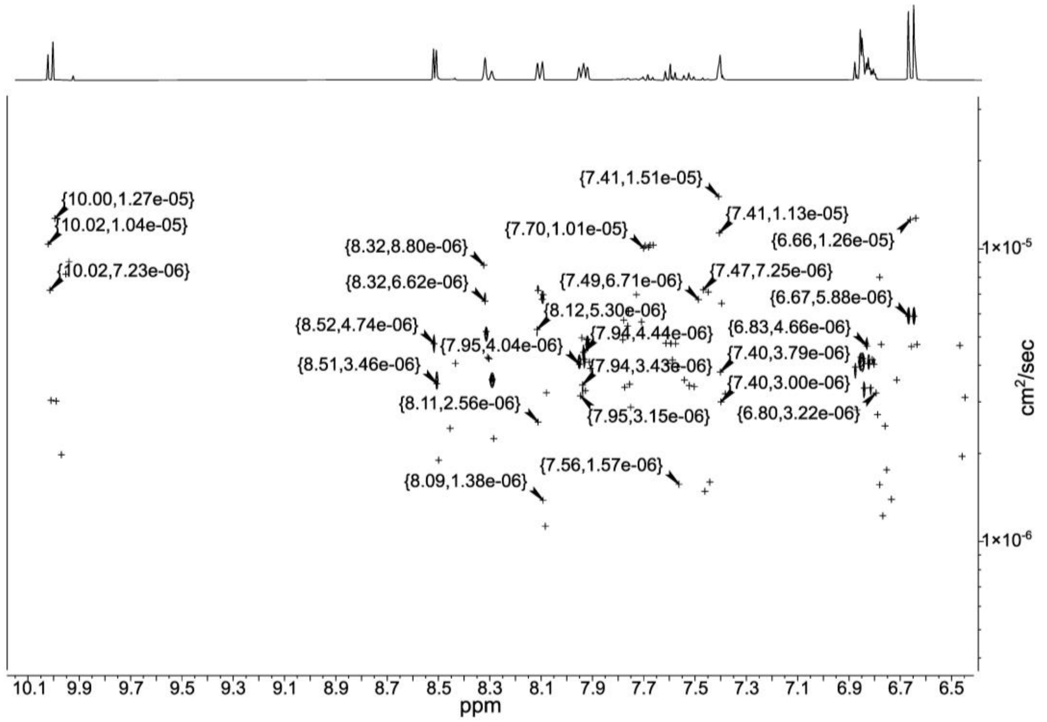

Figure S69. DOSY spectrum (Peak fit mode), of the equilibrium mixture of isophthalaldehyde and **4**, ($CDCl_3$ / CD_3OD 3:1; RT)

From Peak fit mode we obtained the univocal value of $3.46 \cdot 10^{-6}$ cm^2/s as the diffusion coefficient for **7** (from its diagnostic signal at 8.51 ppm, see Figure S69). This value was used as a reference for the monomer. Thus, from Equation S2 (page S34), and the previously reported experiments of acid-induced transimination of CP **2** and **4** at different concentrations, we obtained the DP values reported in Table S5.

Table S5. Diffusion coefficient values obtained from DOSY analysis of **7** and related DP of the assemblies obtained from acid-induced transimination of CP **2** and **4** carried out on solutions in which CP **2**, **4**, and TFA were mixed in a 1 : 2 : 2 ratio, and at different concentrations of CP **2**. In particular, the Peak fit mode was used to obtain accurate and homogeneous values of the diffusion coefficients.

Concentration of imines	Diffusion Coefficient	DP
25 mM	$3.37 \cdot 10^{-6}$ cm^2/s	1
50 mM	$3.27 \cdot 10^{-6}$ cm^2/s	1
100 mM	$1.98 \cdot 10^{-6}$ cm^2/s	5
150 mM	$1.68 \cdot 10^{-6}$ cm^2/s	9
175 mM	$1.46 \cdot 10^{-6}$ cm^2/s	13
200 mM	$1.10 \cdot 10^{-6}$ cm^2/s	31
250 mM	$9.00 \cdot 10^{-7}$ cm^2/s	57
300 mM	$7.45 \cdot 10^{-7}$ cm^2/s	100

^{1}H-NMR spectra of 5 (*a*), 5•2H$^+$ (*b*), 5•2H$^+$ with 4 (*c*), and a 1:2:2 mixture of CP 2, 4 and TFA (*d*)

a) In a 4 mL vial 3.49 mg of **5** (0.03 mmol) were dissolved in 600 µL of $CDCl_3$ / CD_3OD 3:1.
b) In a 4 mL vial 3.49 mg of **5** (0.03 mmol) were dissolved in 500 µL of $CDCl_3$ / CD_3OD 3:1. Then, 100 µL of a 600 mM TFA solution (0.06 mmol) in $CDCl_3$ / CD_3OD 3:1 were added.
c) In a 4 mL vial 3.49 mg of **5** (0.03 mmol) were dissolved in 400 µL of $CDCl_3$ / CD_3OD 3:1. Then, 100 µL of 600 mM TFA (0.06 mmol) and 100 µL of 600 mM **4** (0.06 mmol) solutions in $CDCl_3$ / CD_3OD 3:1 were added.

^{1}H-NMR spectra were recorded and compared with the spectrum (*d*) obtained at 100 mM CP **2**, 200 mM **4**, and 200 mM TFA, in $CDCl_3$ / CD_3OD 3:1.

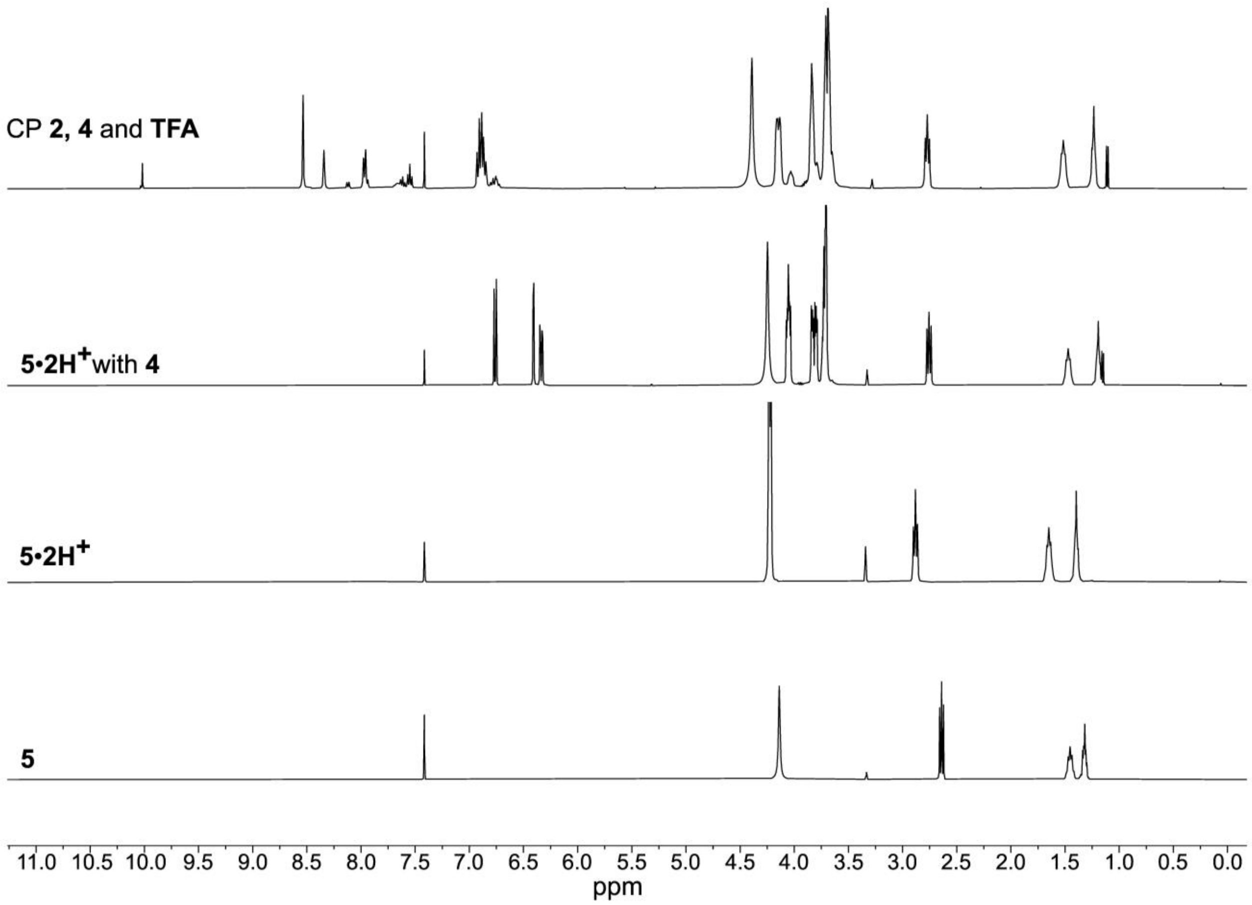

Figure S70. ^{1}H-NMR ($CDCl_3$ / CD_3OD 3:1). From bottom to top: *a*) 50 mM **5**; *b*) 50 mM **5**, and 100 mM TFA; *c*) 50 mM **5**, 100 mM **4,** and 100 mM TFA; *d*) 100 mM CP **2**, 200 mM **4**, and 200 mM TFA.

^{1}H-NMR monitoring of a CCM 1 / 4 mixture 1 : 2 before and after TBA addition

In a NMR tube 85.8 mg of CCM **1** (0.120 mmol) were dissolved in 500 µL of a 480 mM solution of **4** (0.240 mmol) in $CDCl_3$ / CD_3OD 3:1. The solution was heated at 50 °C for 5 days. To this mixture, 100 µL of a 2.4 M solution of TBA (0.240 mmol) in $CDCl_3$ / CD_3OD 3:1 were added at RT, and system evolution was monitored by ^{1}H-NMR (see Figure S69). After 4 h, the system composition did not change anymore on time (see traces *d* and *e* in Figure S69). For restoring the starting state, after 4 days at RT the mixture was heated up at 50 °C for 8 days, and reverting to the initial state was observed.

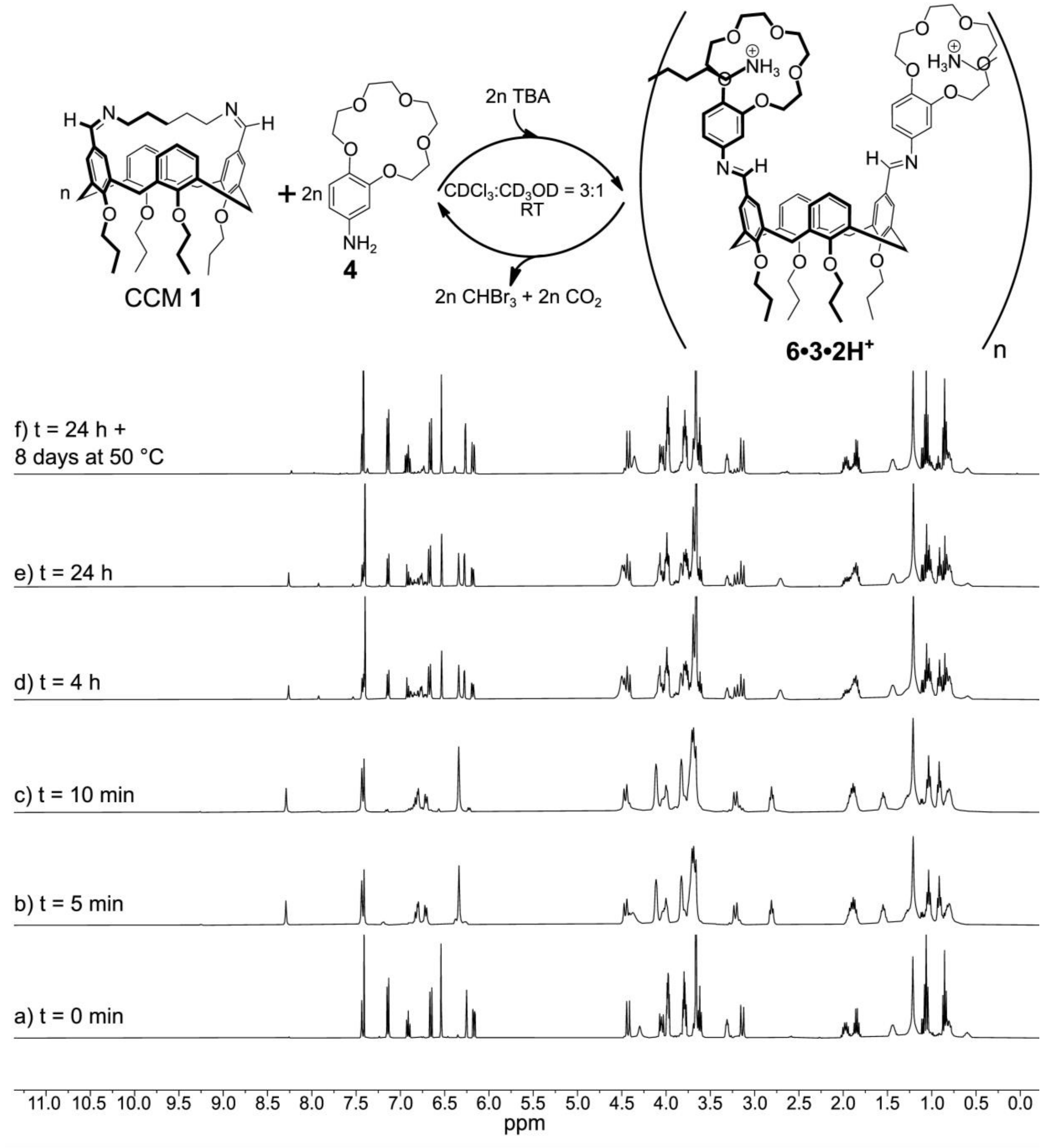

Figure S71. ^{1}H-NMR monitoring of a DL obtained after 5 days from mixing of 200 mM CCM **1** and 400 mM **4** at 50 °C, before (*a*) and after (*b-f*) the addition of 400 mM TBA at RT.

^{1}H-NMR monitoring of a CP 2 / 4 mixture 1 : 2 before and after TBA addition

In a NMR tube 25.7 mg of CP **2** (0.120 mmol) were dissolved in 500 µL of a 480 mM solution of **4** (0.240 mmol) in $CDCl_3$ / CD_3OD 3:1. The solution was heated up at 50 °C for 1 day. To this mixture, 100 µL of a 3.6 M solution of TBA (0.360 mmol, 50% excess) in $CDCl_3$ / CD_3OD 3:1 were added at RT and system evolution was monitored by ^{1}H-NMR (see Figure S70). After 4 h, the system composition did not change anymore on time (see traces *d* and *e* in Figure S70). For restoring the starting state, after 1 day at RT the mixture was heated up at 50 °C for 12 days, satisfactorily reverting to the initial state.

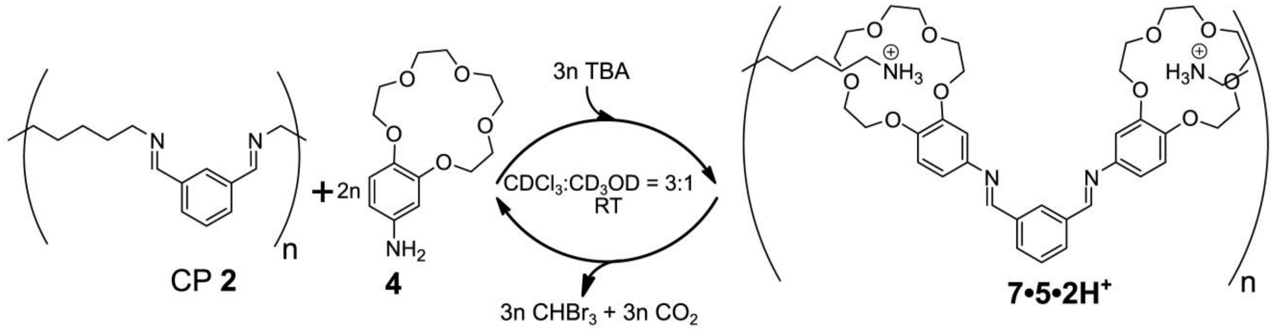

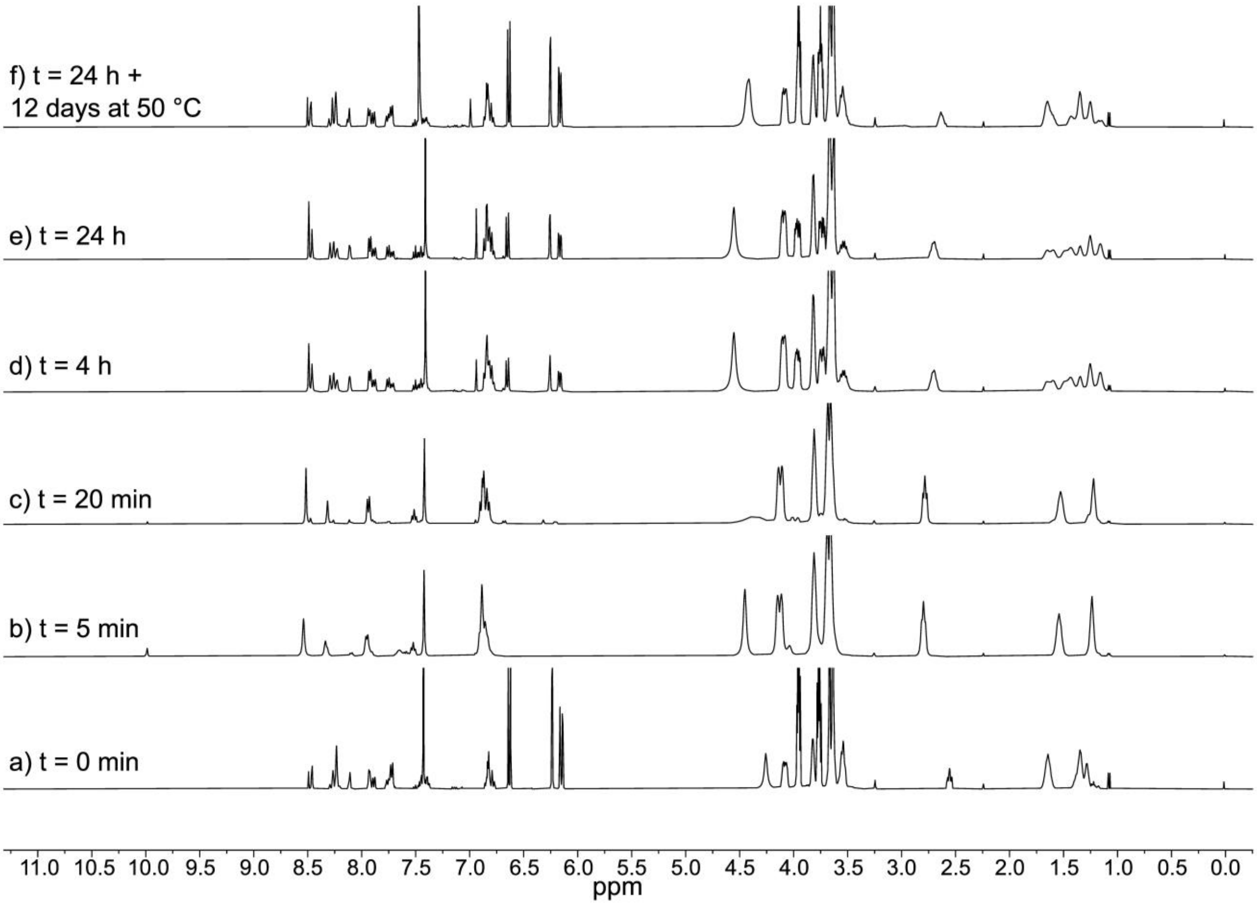

Figure S72. ^{1}H-NMR monitoring of a DL obtained after 1 day from mixing of 200 mM CP **2** and 400 mM **4** at 50 °C, before (*a*) and after (*b-f*) the addition of 600 mM TBA at RT.

Composition comparison for a CCM 1 / 4 mixture 1 : 2 before and after TBA addition

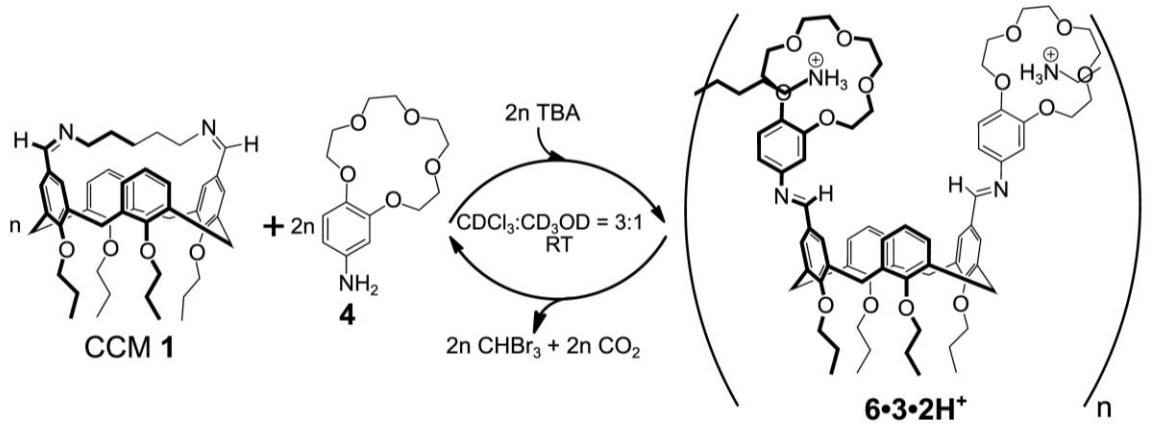

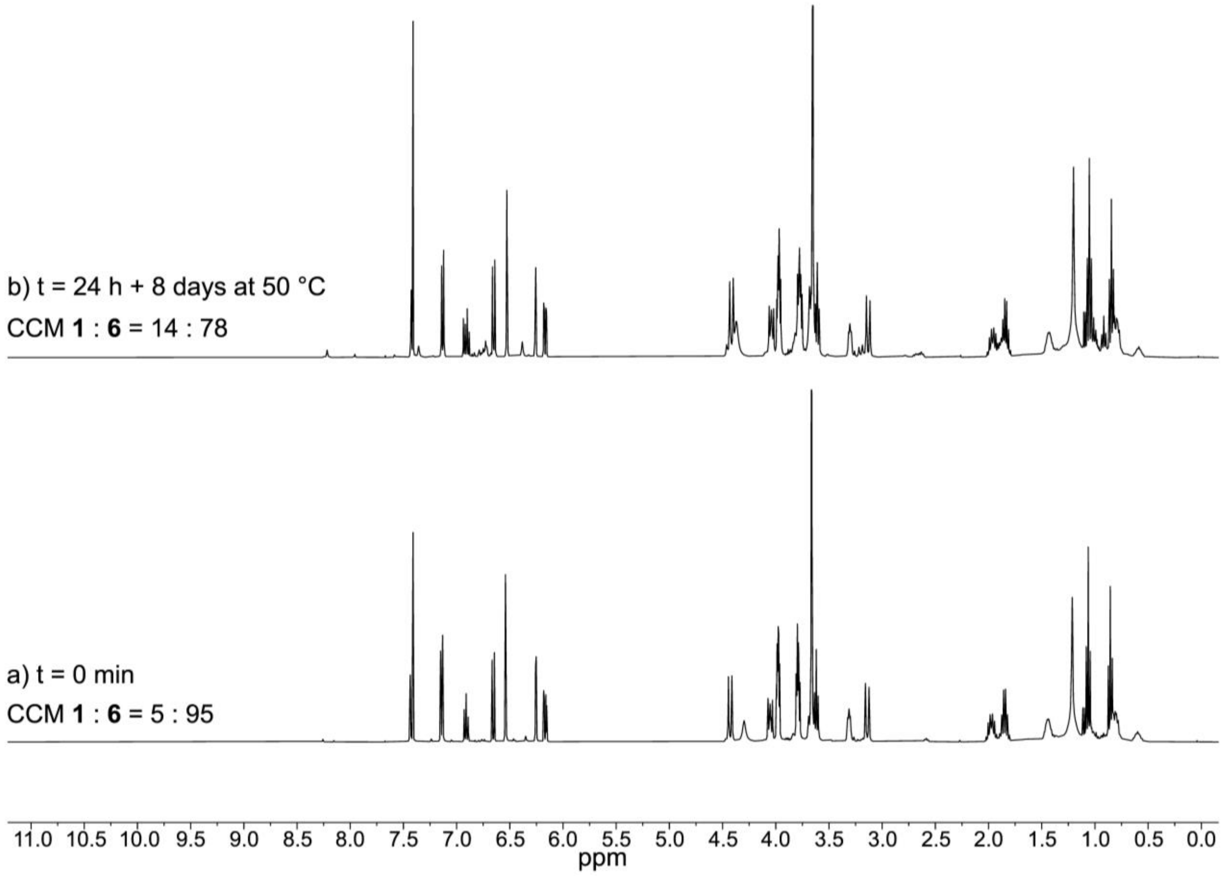

Figure S73. ^{1}H-NMR spectra of a DL obtained after 5 days from mixing of 200 mM CCM **1** and 400 mM **4** at 50 °C, before (*a*) and 8 days of heating at 50 °C after (*b*) the addition of 400 mM TBA at RT. The reported compositions, in terms of percentage, show the similarity between the starting and final state of the kinetic.

Composition comparison for a CP 2 / 4 mixture 1 : 2 before and after TBA addition

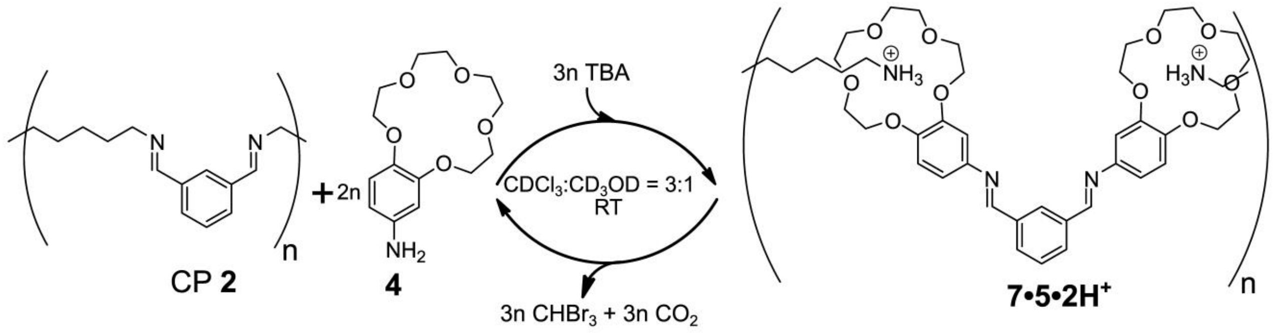

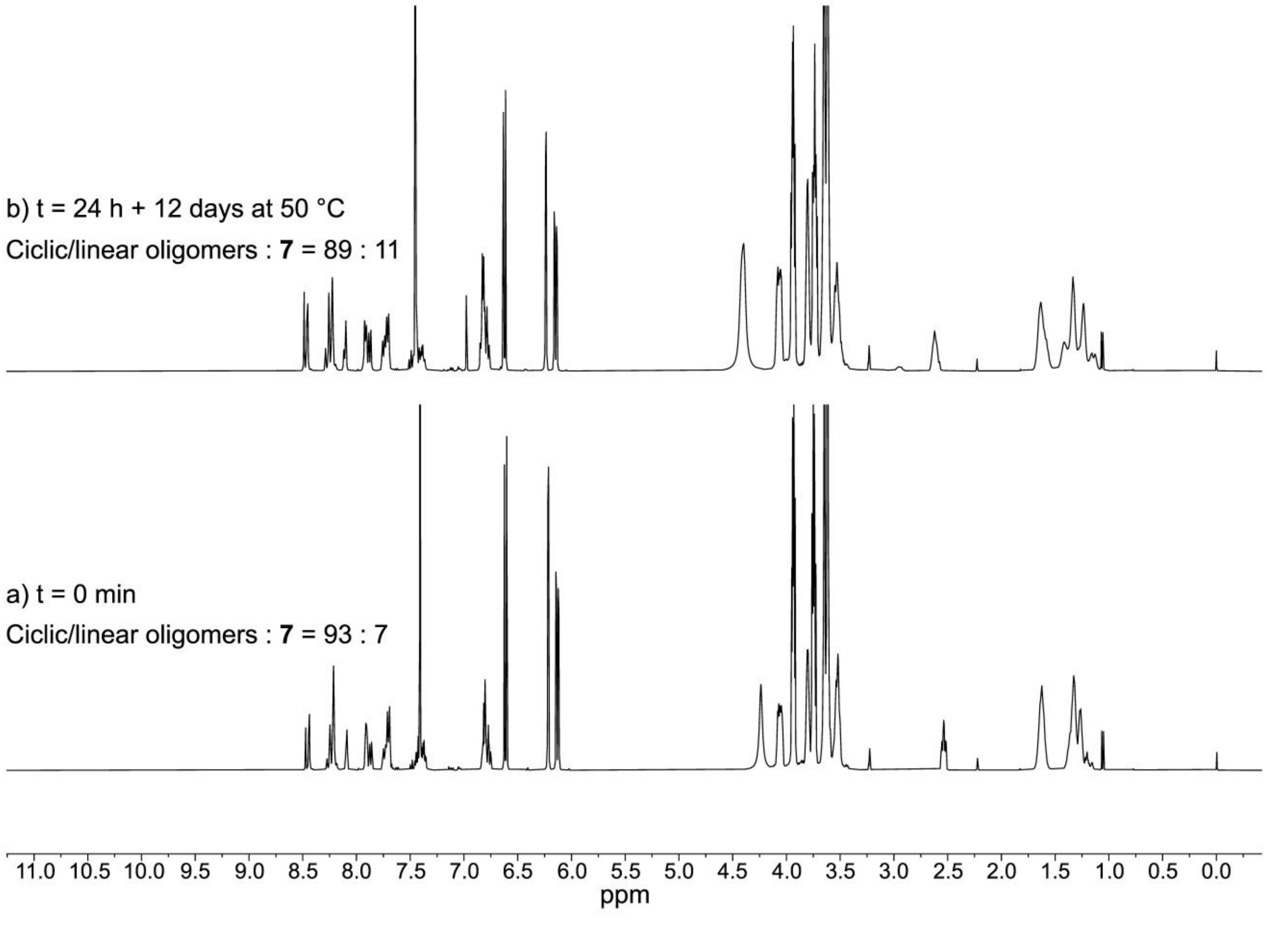

Figure S74. ^{1}H-NMR spectra of a DL obtained after 1 day from mixing of 200 mM CP **2** and 400 mM **4** at 50 °C, before (*a*) and 12 days of heating at 50 °C after (*b*) the addition of 600 mM TBA at RT. The reported compositions, in terms of percentage, show the similarity between the starting and final state of the kinetic.

^{1}H-NMR monitoring of a 1:2 mixture of CP 2 and 4 before and after refuelling experiment

To the final solution of the experiment described at page S57 further 100 µL of a 3.6 M solution of TBA have been added to promote a second refuelling.

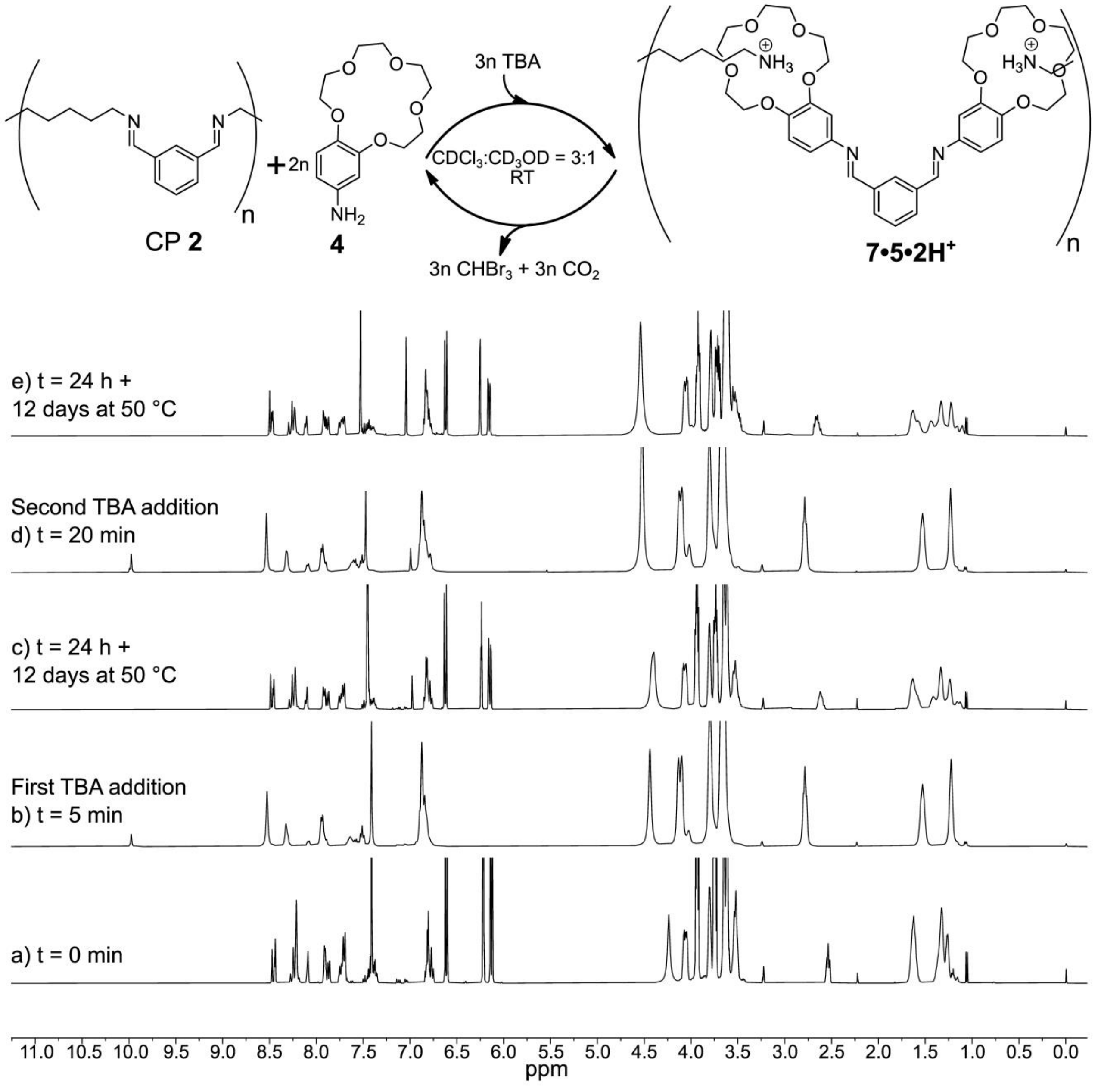

Figure S75. ^{1}H-NMR spectra of a DL obtained after 1 day from mixing of 200 mM CP **2** and 400 mM **4** (trace *a*), 5 min after the addition of 600 mM TBA (trace b), after 12 days at 50 °C (trace c), after a second addition of 600 mM TBA (trace d), and after 12 days at 50 °C (trace e).

Bibliography

[1] Sansone, F.; Barboso, S.; Casnati, A.; Fabbi, M.; Pochini, A.; Ugozzoli, F.; Ungaro, R. Synthesis and Structure of Chiral Cone Calix[4]arenes Functionalized at the Upper Rim with L-Alanine Units. *Eur. J. Org. Chem.* **1998**, 5, 897-905.

[2] Ciaccia, M.; Tosi, I.; Baldini, L.; Cacciapaglia, R.; Mandolini, L.; Di Stefano, S.; Hunter C. A. Applications of dynamic combinatorial chemistry for the determination of effective molarity. *Chem. Sci.* **2015**, *6*, 144–151.

[3] (a) Liu, X.; Xu, J.-F.; Wang, Z.; Zhang, X, *Polym. Chem.* **2016**, 7, 2333-2336. (b) E. M. Hilton, M. A. Jinks, A. D. Burnett, N. J. Warren, A. J. Wilson, *Chem. Eur. J.* **2024**, 20, e202304033.
